# Natural and Anthropogenic Drivers of Genomic Diversity in the California Towhee: Conservation Implications for Peripheral Populations

**DOI:** 10.1111/eva.70313

**Published:** 2026-08-19

**Authors:** Andrew N. Black, Jong Yoon Jeon, Chris McCreedy, Safia Janjua, Erangi J. Heenkenda, Samarth Mathur, Elise Ferree, Amy L. Fesnock, Alvaro Hernandez, J. Andrew DeWoody

**Affiliations:** ^1^ Department of Forestry & Natural Resources Purdue University West Lafayette Indiana USA; ^2^ Western Association of Fish and Wildlife Agencies Boise Idaho USA; ^3^ Point Blue Conservation Science Petaluma California USA; ^4^ Department of Evolution, Ecology and Organismal Biology The Ohio State University Columbus Ohio USA; ^5^ Department of Natural Sciences Scripps and Pitzer Colleges Claremont California USA; ^6^ Cosumnes River Preserve U.S. Bureau of Land Management Galt California USA; ^7^ Roy J. Carver Biotechnology Center University of Illinois at Urbana‐Champaign Urbana Illinois USA

**Keywords:** Endangered Species Act, genome assembly, landscape genomics, local adaptation, ornithology, population structure

## Abstract

Demographic, geographic, and environmental factors such as effective population size, isolation, and habitat availability interact to shape contemporary levels of population genomic diversity. Such factors must be carefully evaluated and contextualized at both the intraspecific and interspecific levels when assessing the conservation status of imperiled species. Here, we studied the population genomics of a perching songbird, the California towhee ( *Melozone crissalis* ), by generating a high‐quality annotated reference genome assembly (N50 = 22 Mb among 627 contigs, max contig size 89.1 Mb) and conducting whole‐genome resequencing on *n* = 81 individual birds sampled from multiple geographic sites across much of the species' range. California towhee sampled from Inyo County and from Southern Oregon, on the periphery of their geographic range, showed reduced heterozygosity and elevated inbreeding compared (a) to other sampled California towhee populations and (b) to related Threatened songbirds. Contemporary effective population sizes (*N*_*e*_) were low for these two peripheral populations, and ancestry patterns of the federally protected Inyo population reveal the remarkably clear genomic signature of a documented, human‐induced demographic bottleneck. Elevated genomic differentiation (*F*_ST_) in the Oregon and Inyo populations, ecological niche modeling, and spatial patterns of gene flow all provide strong support for genetic isolation, limited connectivity, and demographic independence. Both peripheral populations show evidence indicative of local adaptation, as gauged by landscape genetic approaches. Our data highlight the tension that exists between the idealistic goal of conserving all the diversity in a gene pool (e.g., isolated peripheral populations that may contain locally adapted variants) versus the less desirable but more practical goal of focusing expensive conservation efforts on the core gene pool.

## 1 Introduction

Population genomics can provide robust estimates of gene flow, effective population size, population structure, inbreeding, introgression, genetic load, and demographic history, all of which have relevance to conservation (Mathur et al. 2023). In contrast to small panels of putatively neutral markers such as microsatellites or partial mitochondrial DNA (mtDNA) sequences, data from whole‐genome sequences allow conservation managers to plan for the capacity of populations to evolve and adapt in response to future environmental changes (Barbosa et al. 2021; Hohenlohe et al. 2021). Whole‐genome resequencing in increasingly being utilized in cases involving the U.S. Endangered Species Act (ESA), which affords formal legal protection to endangered and threatened species and the habitat upon which they depend (Black et al. 2024; Forester et al. 2022; Waples et al. 2022). The high resolution conferred by genomics can provide insight into how mutation, selection, drift, and gene flow have shaped contemporary diversity levels, which is crucial for both the long‐term population sustainability in the face of environmental flux and for comprehensively evaluating the conservation status of imperiled populations.

**FIGURE 1:**
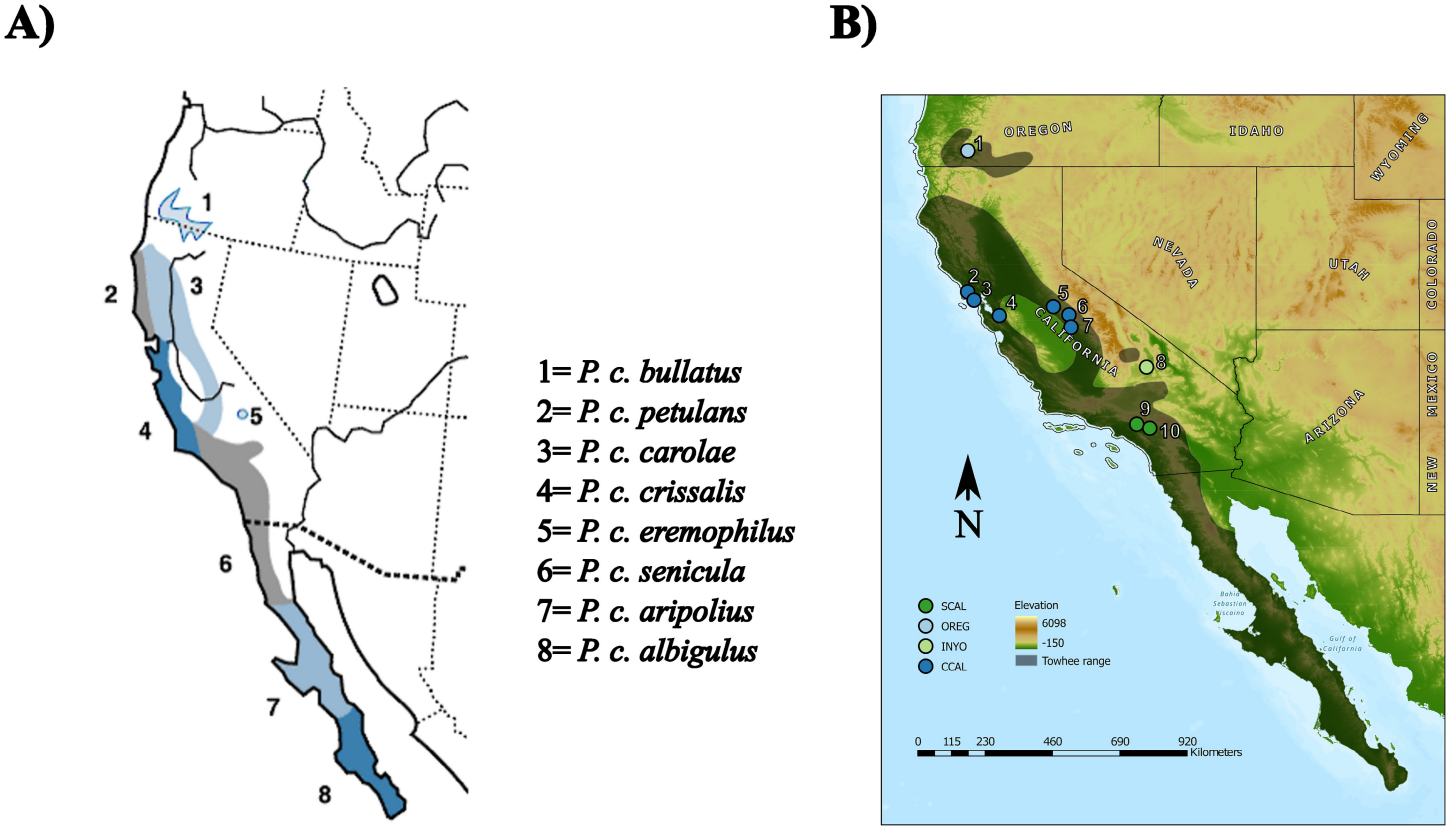
Sampling distribution and current range of California towhee. (A) Subspecies delineated by Kunzmann et al. (2002) [The Latin binomial *Pipilo crissalis* was updated to *Melozone crissalis* by Chesser et al. 2010, such that *Pipilo crissalis* in this figure corresponds to *Melozone crissalis* .] (B) Map of our ten sampling locations (1–10) across the entire range of the California towhee. The ten different sampling locales are categorized into four different regions, each represented by a distinct color: Oregon (OREG), Central California (CCAL), Inyo County, California (INYO), Southern California (SCAL). Basemap credits: Airbus, USGS, NGA, NASA, CGIAR, NCEAS, NLS, OS, NMA, Geodatastyrelsen, GSA, GSI, and the GIS User Community.

The California towhee (*Melozone crissalis*; Chesser et al. 2010) is a sedentary, year‐round resident of coastal scrub, chaparral, and oak woodland habitats distributed over ~695,000 km^2^ from Southern Oregon, USA to Baja, Mexico (Benedict et al. 2020). Davis (1951) nominally described eight subspecies of the California towhee (at that time considered in the genus *Pipilo*): *M. c. bullata, M. c. petulans, M. c. carolae, M. c. crissalis, M. c. eremophila, M. c. senicula, M. c. aripolia* and *M. c. albigula* (Figure 1A). The subspecies delineation of Davis (1951) was based upon geographic color variation in the mantle, crown, breast, and flanks. For the most part, these putative subspecies occur adjacent to one another (i.e., in parapatry) except for *M. c. bullata* (Oregon) and *M. c. eremophila* (Inyo County, California), which are small, isolated populations. However, Patten et al. (2003) indicated that “too many subspecies of the California towhee have been described north of the Mexican border”. Most recently, Pyle (2022) used phenotypic characteristics to subsume *M. c. senicula* and *M. c. aripolia* into *M. c. albigula* and all other subspecies (including the Inyo California towhee) into *M. c. crissalis*, thus reducing the number of subspecies from Davis' eight to only two. Because of this taxonomic flux, we use geographic names (e.g., Inyo or Oregon) to describe populations rather than Latin trinomials throughout most of our paper.

California towhee exhibit strong site fidelity, with individuals defending stable, year‐round territories averaging 0.83 ha and rarely dispersing beyond their natal or established home ranges (Benedict 2008, 2009; Purcell and Verner 1998). The global breeding population is estimated at approximately 7.5 million individuals and has remained stable since the 1960s (Benedict et al. 2020). However, population sizes vary considerably across the species' range, with isolated populations of particular conservation concern. Of particular interest is the Inyo California towhee, a geographically restricted federally protected population endemic to the Argus Mountains of Inyo County.

In 1984, the U.S. Fish and Wildlife Service, hereafter USFWS, proposed to designate the Inyo California towhee as Threatened with Critical Habitat under the ESA (USFWS 1984). The subsequent listing decision associated with the Inyo California towhee was made not because of clear genetic and ecological distinctiveness (which are increasingly important in defining conservation units; Funk et al. 2012). Instead, the listing decision was rendered “because the entire population of this [sic] species is confined to a very limited habitat that has already been altered to some extent and could be adversely impacted by future land use” (USFWS 1987). Due in large part to effective conservation and management efforts, Inyo County population estimates increased from < 175 individuals (Cord and Jehl 1979; USFWS 1984) to over 700 individuals by 2011 (LaBerteaux 2004, 2011).

In 2013, the USFWS proposed to remove the Inyo California towhee from the Federal list of endangered and Threatened wildlife due to recovery (USFWS 2013), but the ultimate decision was still pending as of June 2026. The USFWS summarized habitat improvements and detailed the demographic flux through 2011 but notes the lack of genetic or genomic data to support or undermine the proposed delisting (USFWS 2012). In light of a pending final determination, it is critical to fill this knowledge gap because genetic assessments can reveal continuities and discontinuities in gene pools (Ralls et al. 2018) and because genomic diversity is often associated with individual fitness and population productivity (DeWoody et al. 2021; Reed and Frankham 2003).

This study aims to characterize patterns of genomic diversity, population structure, and local adaptation of the California towhee. We conducted extensive field work to sample individuals across much of their geographic range, generated a contiguous annotated reference genome from a representative California towhee, then performed low‐coverage whole‐genome resequencing on individual birds from different collection sites to provide a comprehensive characterization of the gene pool by evaluating geographic patterns of diversity, population differentiation, natural selection, landscape genomics, and phylogenomic history. We compare our results to related passerine species to provide a contextualized evolutionary backdrop for ongoing conservation efforts. Our discussion largely focuses on the federally listed Inyo population and another peripheral population, which both exhibit reduced diversity due to both anthropogenic and natural demographic bottlenecks.

## 2 Materials and Methods

Here we summarize the methods, with comprehensive details provided in the Supporting Information. Briefly, similar sex ratios (Table S1) of California towhee were collected from Oregon (OREG: site 1), Central California (CCAL: sites 2–7), Southern California (SCAL: sites 9–10), and Inyo County in the Argus mountains (INYO: site 8; Figure 1B). Genomic DNA was extracted from *n* = 81 towhee and Illumina shotgun sequences were aligned to a de novo assembled and annotated reference genome using bwa (v.0.7.17; Li 2013). From these alignments, genotype likelihoods were generated to determine individual heterozygosity and runs of homozygosity with angsd (v.095; Korneliussen et al. 2014) and bcftools (Narasimhan et al. 2016), respectively. For comparative purposes, these two‐diversity metrics were quantified for various passerine species using the same approaches. California towhee population structure was evaluated using pcangsd (v.1.10, Meisner and Albrechtsen 2018), ngsadmix (v.0.95; Skotte et al. 2013), and angsd. Califronia towhee mitochondrial reconstructions were conducted using mitobim (v.1.8; Hahn et al. 2013) whereas nuclear and mitochondrial genomic trees were created using iqtree (v.2.1.2; Nguyen et al. 2015). Spatial patterns of gene flow were assessed using Omniscape (Landau et al. 2021) and ecological niche modeling was conducted using biomod2 (Guéguen et al. 2026) implemented in R (R Core Team 2021).

## 3 Results and Discussion

### 3.1 Reference Genome Sequencing, Assembly, and Annotation

The de novo size of our California towhee genome assembly was 1.41 Gb distributed among 627 contigs (Table S2), which corresponds well to independent measures of genome size. Related species in the genera *Melozone* or *Pipilio* have diploid numbers from 2*n* = 78–84 and cellular DNA content of 1.32–1.55 picograms per cell, which imputes a genome size of 1.29–1.52 Gb in California towhee (1 picogram = 978 Mb; Black et al. 2023; Degrandi et al. 2020; Gregory 2022). The scaffold N50 for our study (the value at which half the assembly is contained in larger contigs) is 22.3 Mb, indicating that many of our contigs likely represent intact chromosomes. For example, the largest contig in our assembly (89.1 Mb) is the size of a typical bird macrochromosome (Table S2). Repetitive regions accounted for about 30% of the genome assembly with a majority of these consisting of transposable elements, with the most abundant element (*CACTA*) comprising roughly 10% of the genome (Table S3).

**FIGURE 2:**
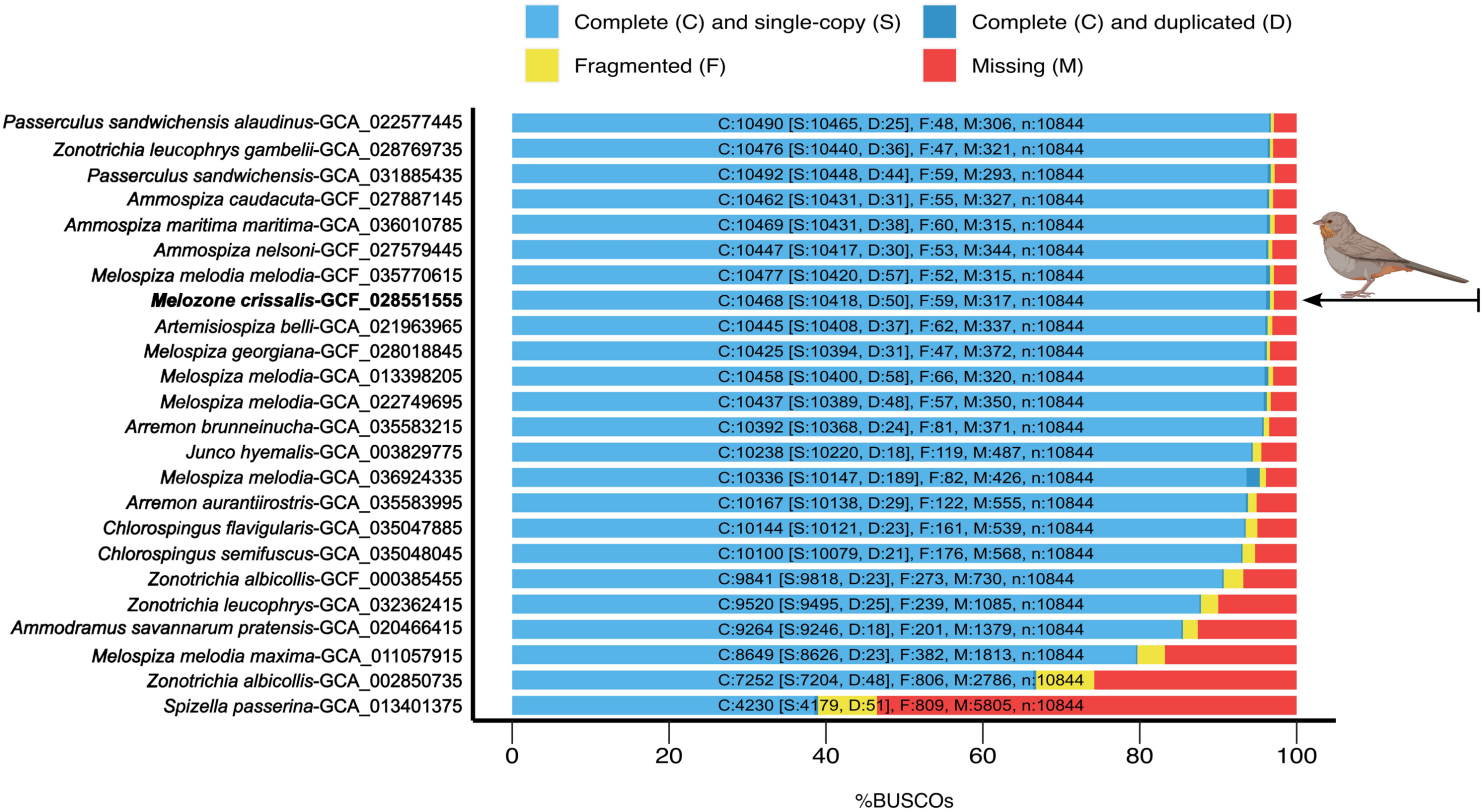
BUSCO plot of genome assemblies. Benchmarking Universal Single‐Copy Orthologs (BUSCO) as an index of genome completeness for California towhee ( *Melozone crissalis* ‐GCF_028551555, in bold). Twenty‐three other Passerellidae species are provided for context, ranging from contig‐level to chromosome‐level assemblies. For each assembly, the genus species ± subspecies + NCBI accession is printed on the *X*‐axis. The California towhee assembly is symbolized with an image of the California towhee created in BioRender (https://BioRender.com/f49d478).

Benchmarking Universal Single‐Copy Orthologs (BUSCO) was used to evaluate the quality and completeness of our genome assembly. Overall, BUSCO identified 10,418 (96%) complete single copy genes in the contiguous sequences, demonstrating high genome completeness relative to 23 publicly available Passerellidae reference genomes (Figure 2). The NCBI Eukaryotic Genome Annotation Pipeline (v10.1) identified 18,501 protein‐coding genes and 29,059 transcripts supported by both RNA‐seq and protein datasets from a variety of related species. Based on the high quality of this annotated assembly, we next leveraged this new resource to evaluate patterns of genomic diversity, natural selection, and gene flow in California towhee.

### 3.2 Genomic Diversity Patterns

Populations with limited or constrained genetic diversity may exhibit reduced adaptive potential and be more prone to the negative impacts of inbreeding (O'Grady et al. 2006). Therefore, we first quantified levels of individual heterozygosity and realized inbreeding. We did so by determining the proportion of the genome containing runs of homozygosity (fROH) among individually sampled California towhee. To provide taxonomic context for the conservation relevance of these towhee diversity metrics, individual heterozygosity and fROH were also calculated for related species of varying conservation concern. Specifically, publicly available passerine sequence data (see Table S4 for complete list) were analyzed and grouped according to the International Union for Conservation of Nature (IUCN) binary “Threatened” (Critically Endangered, Endangered, and Vulnerable) or “Non‐Threatened” (Near Threatened and Least Concern) conservation status. Because the IUCN considers many factors in these threat designations (e.g., extent of habitat, population trends, etc.), this binary grouping (i.e., Threatened vs. Non‐Threatened) effectively accounts for variation in ecology, geography, and demography among species, subspecies and populations by integrating these factors. Thus, our approach provides key biological context for the interpretation of genomic diversity levels across taxa.

**FIGURE 3:**
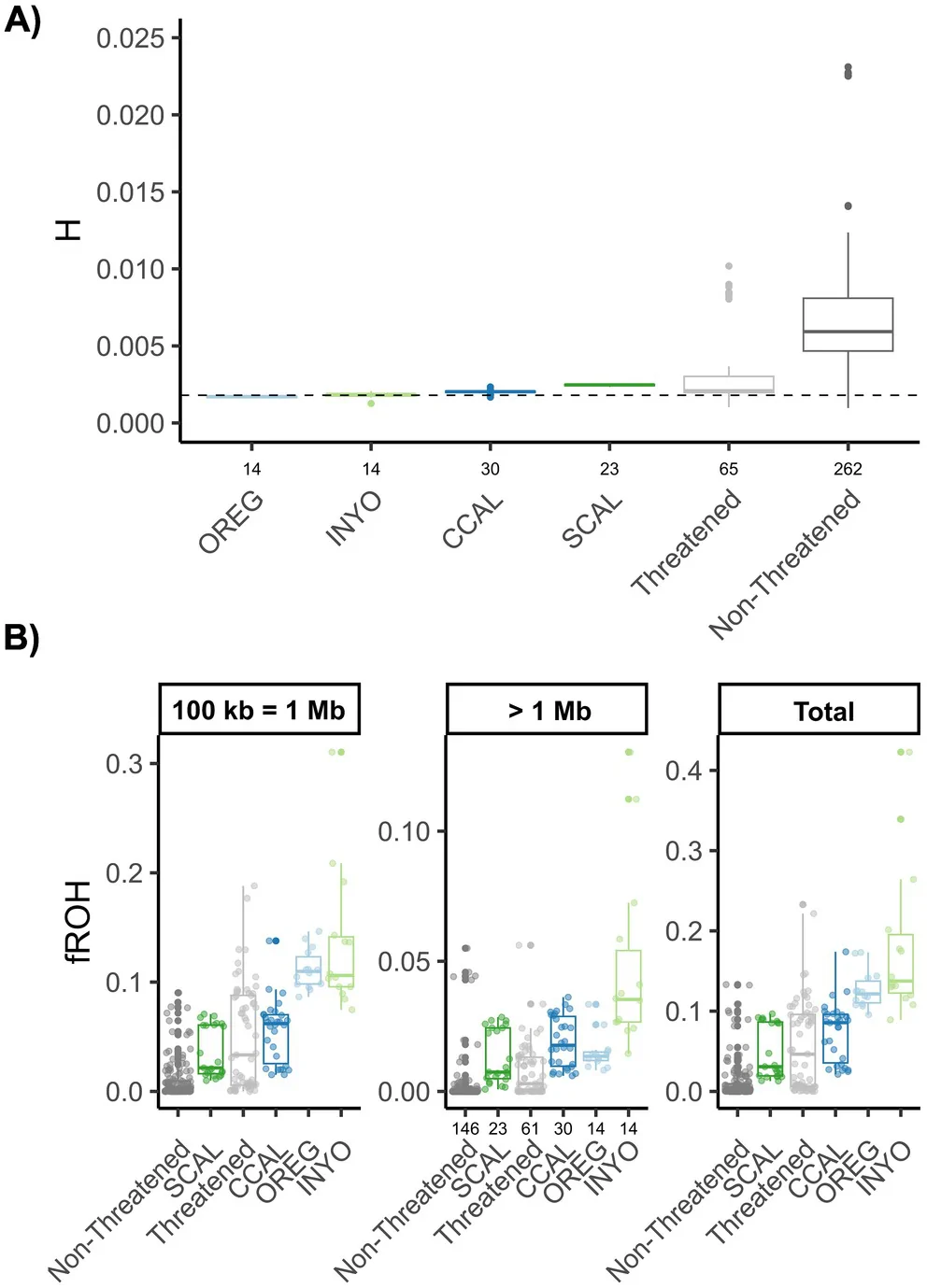
Contextualizing California towhee diversity. Box plots (median, first and third quartile) of (A) individual heterozygosity (H) estimates and (B) runs of homozygosity (fROH) estimates obtained from all sampled California towhee regions and compared to values obtained other passerine (sub)species, which were classified as “Threatened” or “Non‐Threatened” according to the International Union for Conservation of Nature. The horizontal dashed line in (A) represents mean heterozygosity among all sampled California towhee. Sample sizes for each group are printed below each subpanel's *x*‐axis, with the middle panel only printed in (B) as sample sizes for all fROH categories were identical.

Individual heterozygosity (*H*) was evaluated for *n* = 81 California towhees and *n* = 327 individual passerines comprised of 61 (sub)species. In Figure 3A, California towhee were grouped according to sampling region (i.e., OREG, CCAL, INYO, SCAL) and other passerines were grouped according to binary conservation status (i.e., Threatened, Non‐Threatened). The dashed horizontal line in Figure 3A indicates that mean *H* in California towhee (mean *H* = 0.00179) was lower than in related species regardless of their conservation status, signifying that all geographic regions of the California towhee gene pool are shallow (i.e., there is little genomic diversity relative to other passerines). Significant differences in *H* were identified among examined groups (Kruskal‐Wallis *H* = 193.28, df = 5, *p*‐value < 2.2*e*−16), with pairwise Wilcoxon sign rank tests demonstrating that the Non‐Threatened passerine group (mean *H* = 0.0066) had significantly higher mean *H* (compared to all other examined groups, *p*‐value < 3.9*e*−08; Table S5). Compared to the Threatened passerine group (mean *H* = 0.00309), there were no significant *H* differences with CCAL (mean *H* = 0.0020; *p*‐value = 0.465) or SCAL (mean *H* = 0.0025; *p*‐value = 0.740) as shown in Table S5. In contrast, both OREG (mean *H* = 0.00169) and INYO (mean *H* = 0.00179) populations harbored significantly lower *H* compared to the Threatened passerine group (Table S5). OREG and INYO are both peripheral, isolated populations (Figure 1B). Isolation and demographic independence would reduce levels of genetic diversity and increase levels of differentiation due to reduced gene flow and elevated drift (Vucetich and Waite 2003).

We next estimated inbreeding as a function of the ROH burden, where long ROHs (> 1 Mb) represent segments of the genome that are identical by recent descent. Over time (generations), long ROHs are broken down by both point mutations and recombination so shorter ROHs (100 kb −1 Mb) reflect more ancestral inbreeding. To provide additional taxonomic context, fROH values were estimated for *n* = 81 towhee as well as 207 individual passerines comprised of 29 (sub)species. California towhee were again grouped according to sampling region (i.e., OREG, CCAL, INYO, SCAL) and other passerines were grouped according to binary conservation status (i.e., Threatened, Non‐Threatened). The ROH burden was broken into three bins (100 kb −1 Mb, ≥ 1 Mb, Total) to provide temporal context for inbreeding history.

California towhee had higher levels of inbreeding across all three fROH categories when compared to both Threatened and Non‐Threatened passerines (Figure 3B). Significant differences among all fROH categories were identified among groups (Kruskal‐Wallis tests for 100 kb–1 Mb ROH: *H* = 147.28, df = 5, *p*‐value < 2.2*e*−16; > 1 Mb ROH: *H* = 159.93, df = 5, *p*‐value < 2.2*e*−16; total ROH: *H* = 147.31, df = 5, *p*‐value < 2.2*e*−16) with pairwise Wilcoxon sign rank tests shown in Tables S6–S8. Our data indicate that ancestral inbreeding, as measured by shorter 100 kb −1 Mb tracts, were significantly different (Table S6) among most pairwise comparisons but were most elevated in the OREG (mean fROH = 0.1117) and INYO (mean fROH = 0.1345) populations (Figure 3B, 100 kb–1 Mb). This means that elevated ancestral inbreeding (compared to both Threatened and Non‐Threatened passerines) is not new to these two geographically isolated regions. When examining more contiguous runs of homozygosity (ROH length > 1 Mb) that reflect much more recent inbreeding that may be anthropogenically driven, the INYO region was the most consanguineous (mean fROH = 0.04759) and had significantly (*p*‐value < 0.001) higher levels of inbreeding compared to all other groups (Figure 3, > 1 Mb). The recent inbreeding signature of the INYO population is consistent with the known reduction in census population size during the latter portion of the last century, and that signature illustrates the remarkable power of whole‐genome resequencing data to detect genomic patterns resulting from recent demographic processes. Interestingly, there was little evidence of historic inbreeding (ROH length > 1 Mb) in the OREG (mean fROH = 0.0149) population, which exhibited similar non‐significant (*p*‐value ~1) fROH values compared to both CCAL and SCAL (Table S7). However, all California towhee regions except SCAL showed significantly higher levels of recent inbreeding (fROH > 1 Mb) compared to both Non‐Threatened (mean fROH = 0.0023) and Threatened (mean fROH = 0.0077) passerines (Table S7). Elevated ROH burden is broadly consistent with prolonged small population size, which both limits the efficacy of selection and accelerates the accumulation of these genomic segments (Kardos et al. 2018; Ceballos et al. 2018). We therefore estimated contemporary effective population sizes (*N*_e_) to further characterize the demographic history of each California towhee population.

**FIGURE 4:**
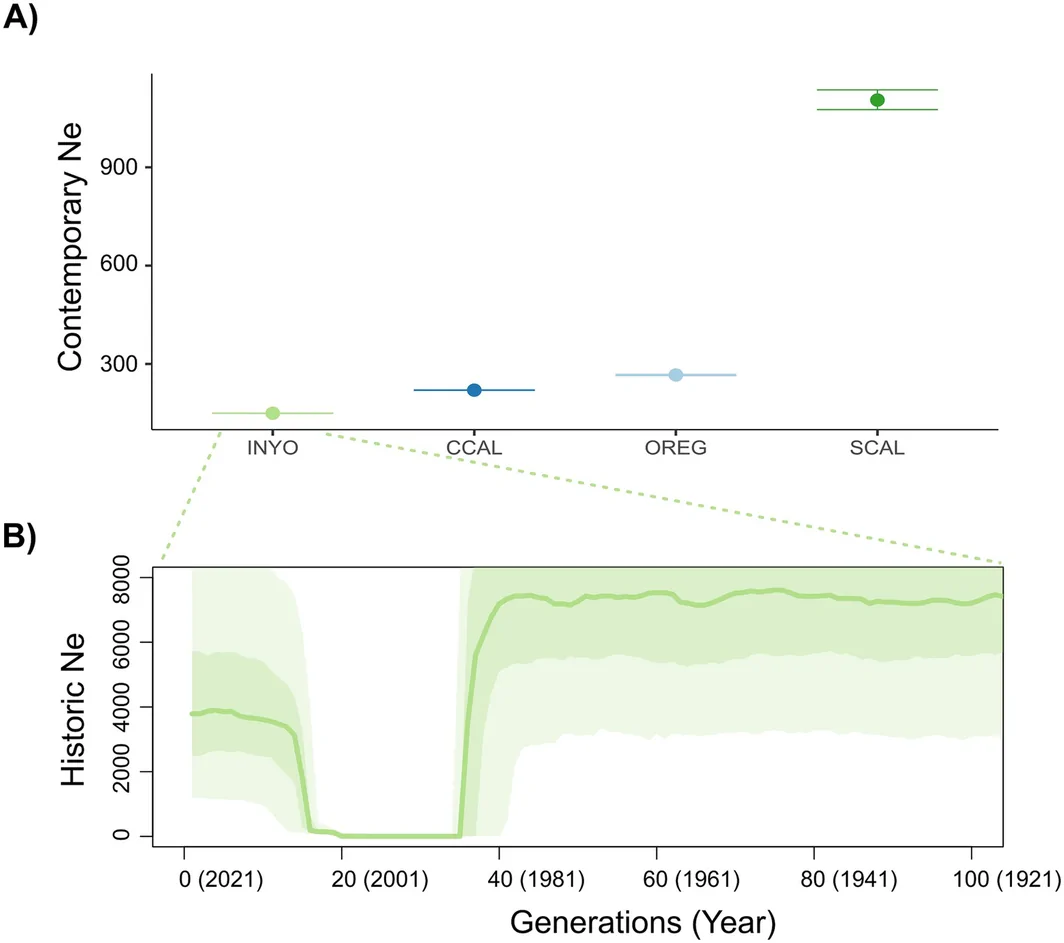
California towhee contemporary effective population size estimates. Linkage disequilibrium based contemporary *N*_e_ estimates for (A) all sampled regions and (B) historic Inyo California towhee *N*_e_ estimates with 95% (light shading) and 50% (dark shading) confidence intervals among 500 iterations for the previous 100 generations, with the estimated year based upon a 2021 field collection date and a generation time of 2 years.

Recent *N*_e_ point estimates (i.e., within the last several generations) based on linkage disequilibrium (LD) for sampled California towhee regions demonstrate that INYO harbored the lowest effective population size (mean *N*_e_ = 149.7) among the four regions and SCAL (mean *N*_e_ = 1105.2) showed the highest (Figure 4A). Focusing on the INYO region where good historical records are available, we note that fluctuations in INYO *N*_e_ over the past 100 generations (as gauged from the whole‐genome sequences) recapitulate the demography (Figure 4B). Assuming a generation time of two years, INYO *N*_e_ over the past 100 generations showed a substantial decline ~1977 which began to increase in the early 2000s (Figure 4B). These *N*_e_ results mirror the documented bottleneck of census population size in the early 1980s (LaBerteaux and Garlinger 1998) and subsequent population expansion in the late 1990s through 2011 (LaBerteaux and Garlinger 1998; LaBerteaux 2004, 2011). The OREG population also showed reduced and declining *N*_e_ over the previous 100 generations, whereas both CCAL and SCAL showed elevated *N*_e_ estimates with precipitous drops in the last several generations (Figure S1).

### 3.3 California Towhee Population Structure and Phylogeography

**FIGURE 5:**
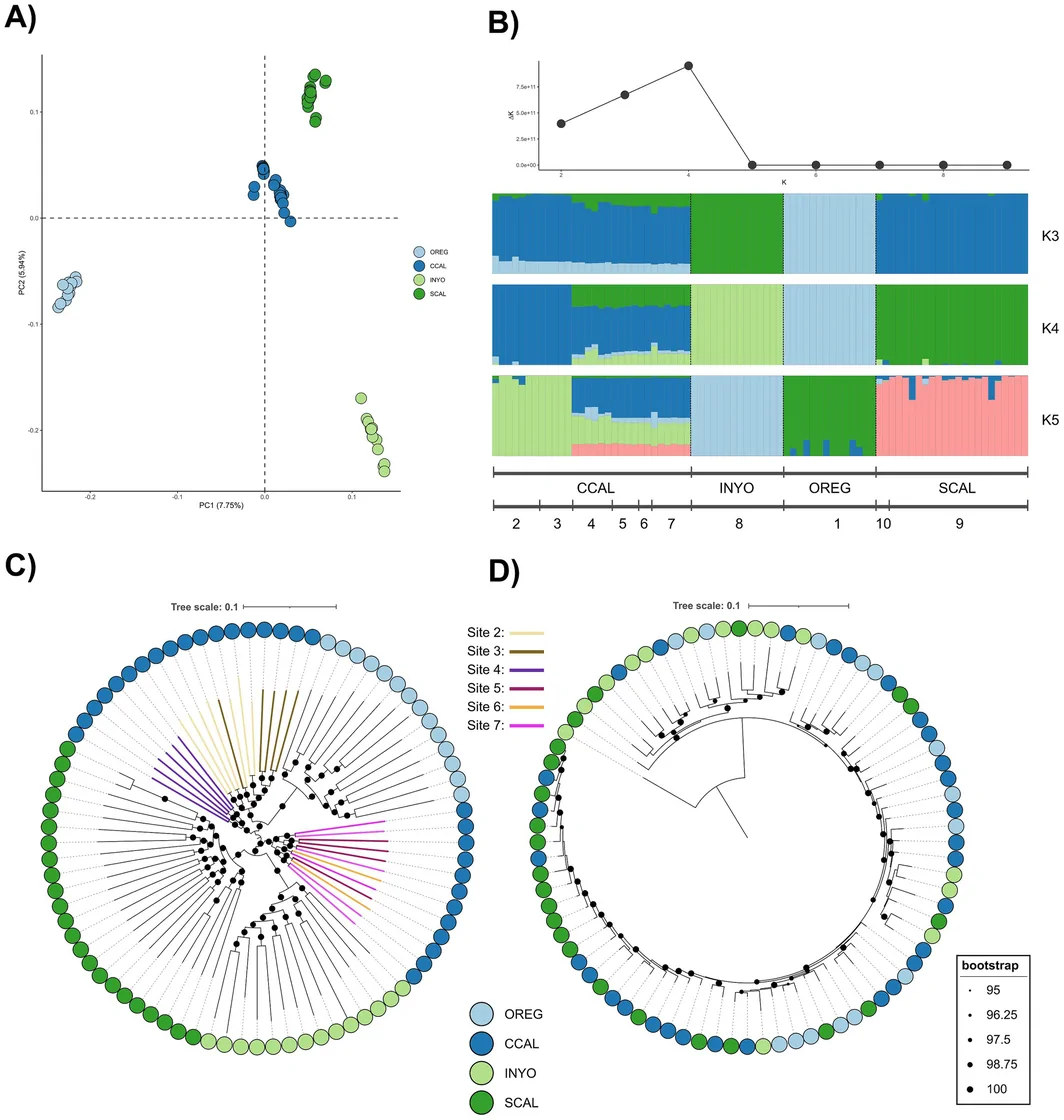
California towhee population structure and phylogenomics. (A) First two axes (PC1 and PC2) from a Principal Component Analysis of allele frequencies obtained from 81 whole‐genome resequencing samples. Individuals are colored by the region where they were collected: Oregon (OREG), Central California (CCAL), Inyo County, California (INYO), Southern California (SCAL). (B) Population structure at varying *K* values (3–5), with bars representing individual admixture proportions for each cluster. The optimal number of groups was identified as *K* = 4. Colors used for *K* = 3 & *K* = 5 vary, with only *K* = 4 colors corresponding to the regional color palette. The sampling locations (sites 1–10) are listed below the regional label. Maximum likelihood trees generated from (C) nuclear (*n* = 81) and (D) mtDNA (*n* = 80, one sample removed due to high levels of missing data) genomes. Each sample (branch) is symbolized by a color corresponding to the geographic region it was collected from. Collection site is also illustrated by colored branches for CCAL. Bootstrap support for nodes (≥ 95%) is symbolized by increasing black diameter circles.

Principal component analysis (PCA) and admixture analyses were performed to assess genomic similarity and ancestry among sampled geographic regions. The model‐free PCA of allele frequency data revealed four non‐overlapping groups, clearly clustering individuals according to sampling region (Figure 5A). Principal Component 1 (PC1) explained 7.8% of the variation and largely differentiated the OREG samples from the others. Principal Component 2 (PC2) explained 5.9% of the variation and discriminated all four regions relatively well. Using a Bayesian model‐based approach to identify population structure, ten independent admixture runs were performed, but only K3 to K5 are illustrated in Figure 5B. The ∆*K* method identified the optimal *K* = 4 (Figure 5B). These results suggest that the OREG and INYO regions represent discrete metapopulations whereas the CCAL region, and to a lesser degree the SCAL region, show substantial admixture.

The phylogeography based on nuclear variation revealed discrete structure (Figure 5C), in sharp contrast to the matrilineal phylogeography (Figure 5D). That is, millions of variable nucleotides across the entire nuclear genome clearly grouped individuals according to sampling region (and in CCAL, generally according to sample site as well) whereas the relatively few variable sites in the maternally‐inherited mitochondrial genome revealed very little structure (although some substructure can be observed in CCAL and SCAL samples). Specifically, the whole‐genome nuclear tree (Figure 5C) from all CCAL samples collected from sites 2 and 3 formed a monophyletic group; samples from site 4 comprised their own discrete lineage, and the branching patterns indicate close evolutionary relationships among genomes sampled at sites 5–7. We note that OREG and coastal sampling locations in CCAL were part of the same clade in the nuclear tree, suggesting that these two populations may have been historically connected (Figure 5C). Overall, the observed discordance in nuclear vs. mitochondrial trees could be due to a variety of factors, including female‐mediated gene flow, different effective population sizes, incomplete lineage sorting, and/or different substitution rates between nuclear and mitochondrial genomes.

We next calculated pairwise *F*_ST_ between all population pairs as a standardized measure of allele frequency divergence, providing a quantitative assessment of population structure. Global estimates of pairwise differentiation between geographic regions were moderate (range = 0.05–0.19) when compared to related songbirds (Provost et al. 2022). The lowest global pairwise *F*_ST_ value was between SCAL‐CCAL sites (*F*_ST_ = 0.05) and the highest *F*_ST_ was between INYO‐OREG (*F*_ST_ = 0.19). The separation between OREG and SCAL (*F*_ST_ = 0.13), as well as OREG and CCAL (*F*_ST_ = 0.11), is likely due in part to isolation by distance. Pairwise *F*_ST_ values are similar among all INYO comparisons, such as the distant SCAL (*F*_ST_ = 0.10) and even the nearby CCAL samples (*F*_ST_ = 0.09), likely due to the relative isolation of the INYO habitat and the demographic contraction that led to the original INYO listing decision.

The low levels of heterozygosity and high ancestral and historic inbreeding observed in the INYO population raise potential concerns about the population's ability to thrive in the face of future environmental changes (Calderón et al. 2024; Harrisson et al. 2019). Therefore, we next sought to query the genome for signatures of selection that might underlie local adaptations. A non‐overlapping sliding window analysis showed substantial peaks in pairwise‐*F*_ST_ comparisons involving INYO populations (Figure S2). Overall, 92 windows had *F*_ST_ values above the 99.9% threshold and those windows contained 28 known protein‐coding orthologs. Gene Ontology analysis showed significant enrichment (*p*‐value = 1.20*E*−06) of genes involved with metabolic processes, especially diacylglycerol (DAG) metabolism (Table S9). While DAG may play an important role in lipid metabolism, metabolic water production, or immune response (Singh and Kambayashi 2016) we could find no such published evidence in desert songbirds.

We next sought to see if there were links between genomic outliers and environmental factors by using redundancy analysis (RDA), a genotype‐environment association approach (Capblancq and Forester 2021). Among the 1430 *F*_ST_ outliers within the 92 windows, we found 40 outliers along RDA axis 1 (Figures S3 and S4) that were significantly associated with temperature seasonality (BIO4), mean temperature of driest quarter (BIO9), annual precipitation (BIO12), or precipitation of driest month (BIO14). These environment‐associated outliers revealed distinct profiles of local adaptation between the eastern and western sampling regions; notably, the region occupied by OREG was the most distinct (Figure S5).

### 3.4 California Towhee Spatial Connectivity and Habitat Availability

**FIGURE 6:**
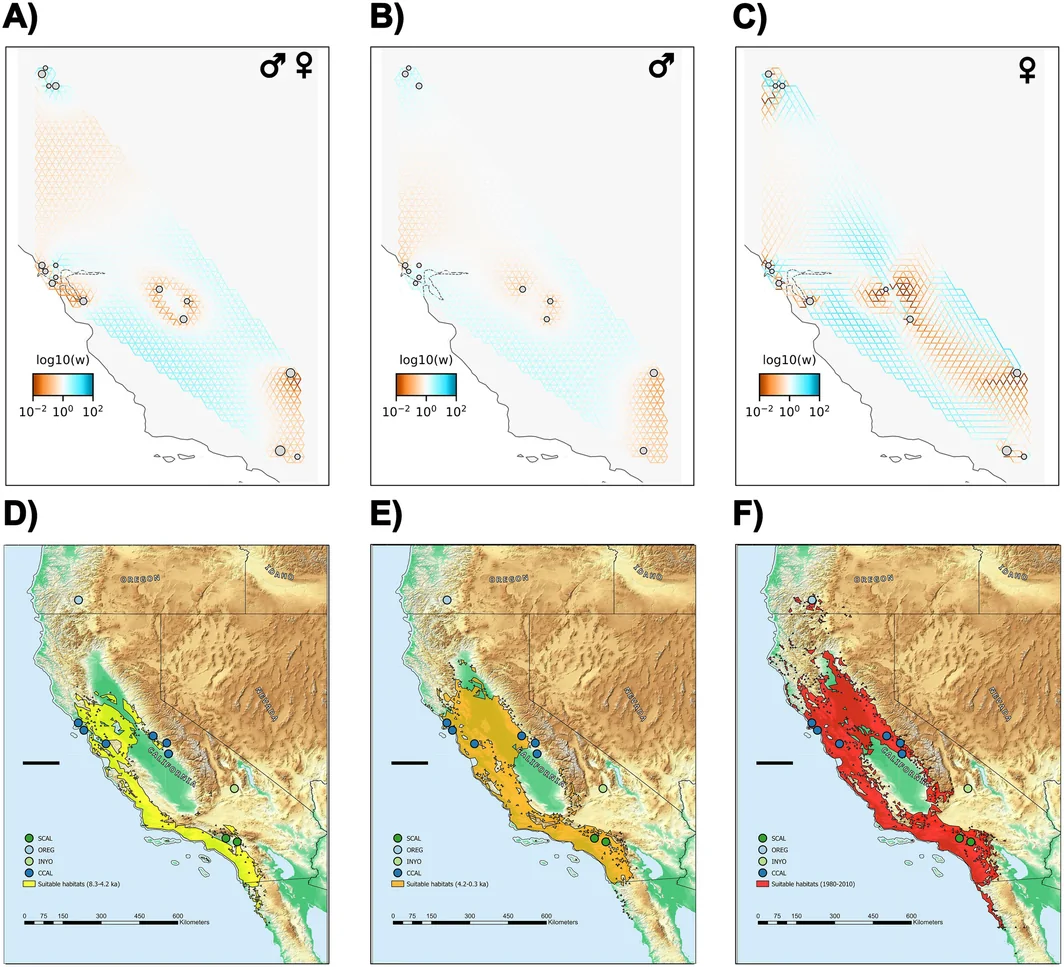
Ecological niche modeling and spatial gene flow. The top three panels show inferred contemporary gene flow patterns of all individuals (A), only males (B), and only females (C) collected among the four study regions. Highest flow values are shown in blue whereas lowest flow values are shown in orange. Gray circles indicate sampling sites. The bottom three panels (D, E, and F) shows modeled habitat availability over time for the California towhee from (A) 8.3 to 4.2 kya, (B) from 4.2 to 0.3 kya, and (C) from 1980 to 2010. Basemap credits: Airbus, USGS, NGA, NASA, CGIAR, NCEAS, NLS, OS, NMA, Geodatastyrelsen, GSA, GSI, and the GIS User Community.

Gene flow is paramount for a population's long‐term persistence due to metapopulation dynamics (Hanski 1998). Landscape‐level analyses can help identify migration corridors and barriers that may be critical to conservation efforts (McRae et al. 2012). Because the high vagility of many avian species often masks their population structure (Kozakiewicz et al. 2018), we utilized landscape genetic approaches to reconstruct gene flow among our study sites (Storfer et al. 2007). To this end, we inferred gene flow patterns on the study landscape to help identify the underlying biological processes (Guerrini et al. 2014). Out of 1000 bootstrap iterations in R package “ResistanceGA” (Peterman 2018) modeling, the geographic distance surface emerged as the best model (average AIC = −113.43), chosen 94.2% of the time (Data S2). The topography surface (aspect, slope) was second best (chosen 5.8% of the time; average AIC = −106.09) while none of the other surfaces (climate, habitat, landcover‐only, all‐variable) were ever chosen as the best model. Subsequent Fast Estimation of Effective Migration Surfaces (FEEMS; Marcus et al. 2021) results for all individuals, all males, and all females are shown in Figure 6A–C. The spatial isolation‐by‐distance (IBD) pattern of all individuals clearly distinguishes each of the OREG, CCAL, SCAL, and INYO regions. A similar pattern of discrimination is observed for all males, but less so for females, where gene flow is more pronounced. Cross‐validation results for *λ* value estimation can be found in Figure S6. Overall, patterns of gene flow suggest migration is facilitated among and around the central population (CCAL; site 2–7), whereas gene flow is more restricted between OREG and CCAL, and around INYO and SCAL.

To trace temporal shifts in habitat availability, we modeled the ecological niche of the California towhee over three ecologically‐relevant timeframes. We did so using a Random Forest algorithm implemented in the R package “Biomod2” (Guéguen et al. 2026) for: (a) the recent past (1980–2010); (b) the late Holocene (4.2–0.3 kya; Meghalayan), and (c) the mid‐Holocene (8.3–4.2 kya; Northgrippian). Projections were based on PaleoClim's bioclimatic variables (Brown et al. 2018; Karger et al. 2017). The final ensemble model, built from three repetitions, demonstrated “excellent” performance (True Skill Statistic (TSS) = 0.833, Area Under the Receiver Operating Characteristic Curve (AUC) = 0.97). Among the predictors, BIO18 (precipitation of the warmest quarter [mm/quarter], ensemble mean importance = 0.20) and BIO12 (annual precipitation [mm/year], ensemble mean importance = 0.13) were the most important factors in defining the most suitable ecological niche. The best habitat suitability was predicted at lower values of BIO18 (approximately 10 in a range of 0 to > 500) and BIOl2 (approximately 500 in a range of 0 to > 3000).

Binary presence–absence projections revealed a progressive increase in suitable habitat availability from 8.3 kya to the present (Figure 6D–F). Notably, suitable habitats in Oregon and the Sierra Nevada–Argus mountains region emerged only recently, forming new connections with central California. Assuming the CCAL and connected SCAL represent historically central populations, the OREG and INYO appear to have been founded more recently as peripheral populations. This spatiotemporal expansion pattern aligns with contemporary gene flow patterns—particularly the limited gene flow and available habitats between OREG and CCAL and between SCAL and INYO.

Davis (1951) speculated that California towhee followed expanding areas of Madro‐Tertiary flora northward from Mexico (later published in Axelrod 1958), casting current day INYO and OREG populations as remnants that currently occupy islands of vegetation that have been isolated by multiple cycles of glaciation, increasing aridity, and fluctuating temperatures. The Sierra Nevada crest impedes movement between the cosmopolitan population on the western slope and small populations in riparian areas on the Sierra Nevada's eastern alluvial fan, south of Owens Lake. Approximately 40 km of low hills, dry rocky outcrops, and Mojave Desert creosote scrub present a physical barrier between these alluvial fan populations and the INYO population. Approximately 65 km of high‐elevation coniferous forest habitat separates the OREG population of the Klamath Basin from Central Valley populations south of Shasta Lake. The OREG population appears to have become increasingly fragmented in recent decades due to development pressures on lower elevation chaparral habitats in the Rogue and Umpqua valleys, with some populations near Roseburg having disappeared (Hunter et al. 1998).

## 4 Caveats and Limitations

All studies have limitations, and ours is no exception. In this study, we generated a highly contiguous and annotated genome of California towhee which will no doubt serve as a genetic resource for future studies. However, analyses of the resequencing data revealed some admixed ancestry in the CCAL region where we collected the sample used for the reference genome (Figure 5B). However, there was no way to know this a priori, and fortunately it did not appear to have caused technical problems (e.g., low mapping rates) with downstream analyses; but it is less than ideal.

Another limitation of our study is that we did not sample birds from sites on the Baja Peninsula in Mexico, despite the fact that many taxa exhibit a phylogeographic break between the peninsula and mainland California (Schierenbeck 2014). We predict that genomic surveys of birds from Baja would reveal even more dramatic aspects to the genetic structure of the California towhee in Mexico. Our study, which encompassed much of the entire range of the species, was not designed to ascertain fine‐scale structure as much as it was to assess the genetic distinctiveness of the Argus mountains (INYO) population relative to other populations. While we were limited in the geographic scope for our spatial genetic analysis (i.e., we sampled 10 locations among four regions), spatial patterns of gene flow are still informative due to the high resolution that whole‐genome resequencing provides (Willing et al. 2012).

Many of the (sub)species used in our comparative dataset differ in ecology, life history, geographic range size, dispersal ability, mating system, and historical demography—all of which are known to influence genome‐wide levels of diversity and inbreeding (Frankham 1996; Leffler et al. 2012). For example, species with naturally small geographic ranges, low vagility, or high philopatry may harbor reduced heterozygosity and elevated fROH simply as a consequence of their biology rather than anthropogenic impacts (Romiguier et al. 2014). Similarly, temporal variance in *N*_e_, mutation rates, and recombination landscapes vary among taxa and no doubt contribute to the substantial variation in *H* and ROH distributions (Corbett‐Detig et al. 2015; Ravagni et al. 2025). Our binary grouping of passerines into “Threatened” and “Non‐Threatened” categories partly accounts for this variation by providing a coarse conservation context, but it cannot fully disentangle the myriad contributions of natural versus anthropogenic processes that shape contemporary genomic diversity. As such, our comparative analyses are best interpreted as providing a general taxonomic context for California towhee diversity metrics rather than serving as a direct yardstick.

A final caveat concerns our *N*_e_ estimates for the CCAL and SCAL regions. Linkage disequilibrium‐based *N*_e_ estimation assumes that the sampled individuals represent a single randomly mating population. However, when admixture is present, allele frequency correlations reflect the mixing of divergent gene pools rather than true demographic history, potentially biasing *N*_e_ upward (Novo et al. 2023). As admixture was detected in both CCAL and SCAL (Figure 4B), *N*_e_ estimates for these regions may be less accurate than those for the more genetically homogeneous INYO and OREG populations.

## 5 General Conclusions

Whole‐genome resequencing of California towhee revealed consistently low levels of genomic diversity relative to other passerine species. In particular, the peripheral California towhee populations (INYO and OREG) exhibited the lowest *H*, highest fROH, and smallest *N*_e_. The INYO population was particularly notable as the genome resequencing revealed significantly elevated recent inbreeding (fROH > 1 Mb) that is entirely consistent with the documented demographic bottleneck of the early 1980s. However, the elevated ancestral inbreeding in both INYO and OREG indicates that contemporary patterns of genomic diversity are not solely the product of recent anthropogenic disturbance. Population structure and phylogeographic analyses of genome resequencing data revealed four distinct geographic regions, with the INYO and OREG sites representing isolated, differentiated metapopulations with limited gene flow.

A reduction of genetic diversity has been observed in many California taxa, including the California gnatcatcher, southern sea otter, Santa Ana Mountains puma, and the Channel Island fox, because of some combination of restricted range, historical bottlenecks, habitat fragmentation, and prolonged small *N*_e_ (Larson et al. 2002; Ernest et al. 2014; Sesser et al. 2019; Robinson et al. 2016; Vandergast et al. 2022). The evolutionary processes underlying the low comparative diversity observed in the California towhee remain incompletely resolved, but likely reflect demographic processes (e.g., slow population growth and limited gene flow among isolated populations) associated with the pronounced environmental variation associated with California landscapes (Ravagni et al. 2025).

The abundant center model (Sagarin and Gaines 2002) posits that species abundance is generally expected to be highest at the geographic range center, with population sizes declining toward the range peripheral (Vucetich and Waite 2003; Lawton 1993). Accordingly, the central‐marginal hypothesis predicts a reduction in genetic diversity and an increase in genetic differentiation toward the margin of a species range (Antonovics 1976; Brussard 1984; Eckert et al. 2008). Thus, peripheral populations are naturally expected to have reduced levels of genetic diversity and higher levels of differentiation due to reduced gene flow and elevated drift (Vucetich and Waite 2003). We observed these patterns in the INYO and OREG populations. Furthermore, ecological niche modeling, phylogeographic, and landscape genomic analyses all collectively support the central‐marginal hypothesis as a likely driver of the biological processes that produced the patterns we observed in the California towhee. Suitable habitat in Oregon and the Argus Mountains emerged only within the last few thousand years, and both regions show restricted gene flow (Figure 6), high differentiation (Figure 5), and low diversity (Figure 3) relative to the historically continuous central California population (Figure 6). Signatures of local adaptation, particularly for genes associated with metabolic and climate‐related processes, further indicate that peripheral isolation may not have been entirely neutral. For the INYO and OREG populations, the absence of connectivity and small effective population sizes would seem to leave these populations with limited resilience to future environmental change, warranting continued conservation monitoring and management attention.

Overall, our study demonstrates how demographic drivers of genomic diversity leave lasting signatures across the genome that can be deciphered and partitioned into natural and anthropogenic components, providing invaluable context for conservation and management decisions. Looking forward, more complete spatial sampling across the California towhee range (particularly along the transitional zones between central and peripheral populations) would provide an explicit test of the central‐marginal hypothesis and further resolve the relative contributions of isolation, drift, and local adaptation to the genomic landscape of this species. Ultimately, we hope that the genomic infrastructure, analytical approaches, and ecological context provided here will serve as a foundation for future work aimed at sustaining healthy, genetically resilient animal populations in the wild.

## Funding

This work was supported by U.S. Bureau of Land Management (Grant L20AS00045), National Institute of Food and Agriculture, and United States Fullbright.

## Conflicts of Interest

The authors declare no conflicts of interest.

## Supporting information


**Figure S1:** Linkage disequilibrium based historic effective population size (*N*_e_) estimates for (A) Oregon (OREG), (B) Central California (CCAL), and (C) Southern California (SCAL) with 95% (light shading) and 50% (dark shading) confidence intervals among 500 iterations for the previous 100 generations. Note, the three sub‐panels have different *Y*‐axis scales.
**Figure S2:** Fst sliding window analysis. Non‐overlapping sliding window analysis (50 kb) to illustrate differentiation (pairwise‐*F*_ST_) between sampling regions (Oregon = OREG, INYO = Inyo County, CCAL = Central California, SCAL = Southern California). For the pairwise INYO comparisons, genes found within windows above the horizontal red dashed lines (> 99.9%) were used for Gene Ontology analysis.
**Figure S3:** Triplot of redundancy analysis. BIO9, BIO11, and BIO14 were the main drivers of environmental adaptation along RDA axis 1, while BIO12 was the primary driver along axis 2. Points are color‐coded to represent loci significantly associated with corresponding bioclimatic variables. All bioclimatic variables used are shown as arrows, with four predictors of the environment‐associated loci along the only statistically significant RDA axis (axis 1) highlighted in color. Axes 1 and 2 explained 49% and 18% of the variation, respectively, and the adjusted *R*‐squared value of the model was 0.113.
**Figure S4:** Manhattan plot of loci included in the redundancy analysis with their loading scores on the RDA axis 1. Chromosomes containing loci are depicted, and points are color‐coded according to their significance (below or above 2 SD from the mean).
**Figure S5:** Adaptive index distribution across the entire species range. The gradient colors represent spatial differentiation in local adaptation, where each pixel's score is computed as the sum of standardized bioclim variables weighted by their loadings on the corresponding RDA axis.
**Figure S6:** Leave‐one‐out cross‐validation results of (A) all individuals, (B) males only, (C) females over *λ* values in the range of (1*e*−6, 1*e*2). The plots show *λ* values on *x*‐axis and cross‐validation errors by L2 distance on *y*‐axis.
**Table S1:** List of towhee (*n* = 81) collected from ten different sampling sites. For phenotypic and genotypic sex assignment, U is unknown, M is male and F is female.
**Table S2:** Assembly metrics from the Inyo California genome assembly.
**Table S3:** Summary of the transposable element (TE) repertoire for the California towhee reference genome generated with edta (Ou et al. 2019).
**Table S4:** Passerine genomes and associated Sequence Read Archive (SRA) files used to contextualize California towhee diversity metrics. The species of the associated SRA file, the N50 of the downloaded (FTP) genome, and SRA ID are listed for each sample. For each sample, Individual Heterozygosity and the frequency of Runs Of Homozygosity are reported, in addition to alignment statistics (Breadth, Depth, and Mapping %). The Table contains the unfiltered list. Individuals rows were removed based upon minimum N50 (> 22 Mb) and species sample size (≥ 2) prior to calculating/retaining final fROH values.
**Table S5:** Bonferroni corrected *p*‐values obtained from pairwise Wilcoxon sign rank tests for differences in individual heterozygosity between California towhee regions. Bold cells are statistically significant (*p*‐value < 0.05).
**Table S6:** Bonferroni corrected *p*‐values obtained from pairwise Wilcoxon sign rank tests for differences in the proportion of Runs of Homozygosity (fROH) for lengths 100 kb–1 Mb. Bold cells are statistically significant (*p*‐value < 0.05).
**Table S7:** Bonferroni corrected *p*‐values obtained from pairwise Wilcoxon sign rank tests for differences in the proportion of Runs of Homozygosity (fROH) for lengths > 1 Mb. Bold cells are statistically significant (*p*‐value < 0.05).
**Table S8:** Bonferroni corrected *p*‐values obtained from pairwise Wilcoxon sign rank tests for differences in the proportion of Total Runs of Homozygosity (fROH). Bold cells are statistically significant (*p*‐value < 0.05).
**Table S9:** Fst outlier results. California towhee genes identified within outlier 50 kb windows. Note, only genes with known orthologs were used for gene ontology enrichment analysis (in bold).

## Data Availability

Unprocessed shotgun and HiFi sequence data have been deposited to NCBI Sequence Read Archive under Bioproject PRJNA915193. The assembled Refseq genome can be found under accession GCF_028551555.1 along with NCBI *Melozone crissalis* Annotation Release 100. All code and scripts have been uploaded to github (https://github.com/Andrew‐N‐Black/towhee).
