# Supplementary material for: Natural and Anthropogenic Drivers of Genomic Diversity in the California Towhee: Conservation Implications for Peripheral Populations
Source: Evol Appl. 2026 Aug 19;19(8):e70313. doi: 10.1111/eva.70313 (PMC13490859; doi:10.1111/eva.70313)
Supplement: Supplementary file 1 [file EVA-19-e70313-s001.docx]

**SUPPORTING INFORMATION**

**FOR:**

Natural and anthropogenic drivers of genomic diversity in the California towhee: conservation implications for peripheral populations

**Detailed Materials and Methods**

**Sampling, DNA extractions and molecular sexing***.* California towhee (n=81) were collected between 2020-2021 using mist nets or Potter traps from ten different sites across Oregon and California, spanning much of the species’ range. Blood was collected from n=14 samples from Oregon (site 1; OREG), n=30 from central California (both sides of the San Joaquin Valley; site 2-7; CCAL), n=14 from Inyo County California near the Argus mountains (site 8; INYO), and n=23 from southern California (site 9-10; SCAL) as illustrated in Figure 1B. From these blood samples, DNA was extracted from blood samples using a Qiagen DNeasy Blood & Tissue Kit following the manufacturer’s standard protocol. All California towhee were sexed phenotypically in the field and then confirmed genotypically in the lab using the assay of Griffiths *et al.* (1998), whereby males (ZZ) or females (ZW) produce either one or two amplicons, respectively, which can be discerned on an agarose gel.

**Reference genome sequencing, assembly, and annotation.** A genotypically sexed female (Internal Sample ID=I_8) collected from site 2 in CCAL (Figure 1B) was selected to create the California towhee reference genome. High molecular weight (HMW) DNA was extracted from blood for genome sequencing and assembly using a Monarch® HMW DNA kit. PacBio library construction and sequencing was performed at the Roy J. Carver Biotechnology Center (University of Illinois at Urbana-Champaign) after shearing HMW genomic DNA to an average size of 15 kb with a Megaruptor 3 (Diagenode). The PacBio libraries were constructed using the SMRTbell Express Template Prep kit version 2.0 (Pacific Biosciences, CA), quantified with a Qubit, and expected fragment size distribution confirmed using a Femto Pulse (Agilent, CA). These libraries were sequenced using 4 SMRT cells 8M trays on a PacBio Sequel IIe using a 30 hr movie time. Following sequencing, a circular consensus sequencing (CCS) analysis was conducted using default parameters in SMRT Link V10.1, which specifies at least 3 passes and a minimum accuracy of 99%. The four PacBio SMRT cells yielded 59 Gb worth of sequence data distributed among 3.8 million reads with a mean N50 polymerase read length of 15.5 kb. After filtering reads based upon sequence length, 98% of the data were retained (i.e., 57 Gb and 3.7 million reads producing >40X estimated coverage). After removing CCS reads that were < 5 kb or > 50 kb in length with fastp (v 0.23.2; Chen *et al.*, 2018), hifiasm (v.0.18.5; Cheng *et al.*, 2021) was used to assemble the PacBio reads. Following *de novo* genome assembly, purge_dups (Guan *et al.*, 2020) was used to identify and remove haplotigs (nearly identical sequences) from the assembly. To identify and remove potential contaminants, sequences were taxonomically classified using blobtoolkit2 (v.2.1; Challis *et al.*, 2020). After removal of non-target sequences, genome summary metrics were generated with quast (v.3.2; Gurevich *et al.*, 2013) and genome completeness was evaluated using the busco (v.5.2.2; Seppey *et al.*, 2019) Passeriformes (odb10) lineage. Following curation of the genome assembly, gene annotation was completed using the NCBI Eukaryotic Annotation Pipeline. Transposable element profiling was conducted using edta (Ou *et al.,* 2019).

**Whole-Genome Resequencing data.** California towhee genomic reads (n=81 samples) generated for the current study as well as all passerine data available (as of September 14^th^, 2024) from NCBIs Sequence Read Archive (SRA) were processed in parallel. Illumina shotgun sequencing of 81 towhee were performed using the Illumina NovaSeq 6000 platform at the University of Illinois (Roy J. Carver Biotechnology Center). The libraries were prepared using Illumina TruSeq reagents and sequenced on a S4 flowcell using 2x151-bp chemistry. SRA data were selected if they met the following criteria: 1) paired-end fastq data ≥4.2 Gb (i.e., at least 3x estimated coverage); 2) whole-genome data sequenced on an Illumina platform (excluding MiSeq); and 3) the species or sub-species had a reference genome assembly. We excluded documented hybrids (e.g., species A x species B), linked reads (e.g., 10X genomics & Hi-C libraries), and for computational tractability we picked a single SRA (i.e., highest bases sequenced) if the same BioSample was sequenced multiple times and capped the number of BioSamples for a (sub)species at n=30.

**Bioinformatic processing.** fastqc (v0.11.9; Andrews, 2010) was used for quality assessment of the whole-genome resequencing data (SRA & California towhee reads). For each (sub)species, paired-end Illumina reads were aligned to unambiguous non-repetitive regions in sequences ≥ 100 kb of each (sub)species respective downloaded and indexed reference genome using the bwa (v.0.7.17; Li, 2013) mem algorithm. For each reference genome, ambiguous regions were determined by calculating mappability (<1) with genmap (v.1.3.0; Pockrandt *et al.*, 2020), which was run using 100-mers and allowing up to two mismatches. Repetitive regions were determined by running repeatmasker (v.4.07; Smit *et al.*, 2015) and specifying the Melozone repeat library. Alignments were sorted based upon read coordinate and PCR duplicates were identified using picardTools (Broad Institute, 2019). Mapped reads were realigned around insertions/deletions using gatk3 (v.3.6.0; McKenna *et al.*, 2010). Following alignment, samtools (v.1.8; Li *et al.*, 2009) was used to remove (bitflags italicized) unmapped (*4*), secondary (*256*), QC failed (*512*), duplicate (*1024*), supplementary (*2048*) alignments, and those that were not properly paired (2). Data was further culled if samples had less than: 3x depth of coverage, 75% 1x genome breadth, or <75% mapping rate.

**Passerine and California towhee genomic diversity***.* The quality filtered binary alignment files were then used as input for angsd (v.0.929.55; Korneliussen *et al.*, 2014), specifying the samtools model (-GL 1). Individual heterozygosity (*H)* was estimated for all samples (SRA & California towhee) from the genotype likelihood data using the site frequency spectrum and was measured as the number of heterozygous sites divided by the total number of sites analyzed across each respective genome. Minimal filtering was utilized (-minQ 30), but a minimum read depth of 3 (-mininddepth 3) was required to retain each site. Runs of homozygosity (ROHs) were identified using bcftools (v.1.14; Narasimhan *et al.*, 2016) default parameters. The proportion of ROHs (fROH) was calculated to estimate individual inbreeding where fROH is the proportion of the total genome length that is homozygous for 100 kb-1 Mb regions (ancestral inbreeding), >1 Mb regions (recent inbreeding), and cumulatively (McQuillan *et al.*, 2008). Only SRA files associated with reference genomes similar to the California towhee reference contiguity (i.e., N50≥22Mb) were used to estimate fROH for (sub)species containing at least two samples.

**California towhee population structure, phylogeography, and differentiation.** A model-free Principal Component Analysis (PCA) was performed on a covariance matrix calculated from the genotype likelihoods with pcangsd (v.0.9757; Meisner & Albrechtsen, 2018) after filtering to remove minor alleles (-maf 0.05). To ensure sites were well represented among California towhee samples, only those present in at least 80% of the samples (>64) with a minor allele frequency of 0.05 and a p-value cutoff of 10^-6^ were used for analyses. We identified 18,107,254 variant sites in the California towhee samples, with 8,087,130 polymorphic sites remaining after quality filtering with the above approach. We used a model-based approach to estimate K by identifying the most likely number of genetic populations that minimize deviations from Hardy-Weinberg equilibrium while determining the admixture proportions of each sample. This was conducted by leveraging genotype likelihoods as input for ngsadmix (v.2; Skotte *et al.*, 2013) and evaluating K values from 1 to 10, with ten replicate runs for each K value (i.e., 100 total tests). The clumpak webserver (Kopelman *et al.*, 2015) was used to determine the most likely K according to the Evanno *et al.* (2005) method. Files were then imported into clumpp (v.1.12; Jakobsson & Rosenberg, 2007) for aggregation of individual runs, and the merged Q-matrices were illustrated using the R package pophelper (v.2.3.1; Francis, 2017).

To evaluate the phylogenomic and phylogeographic relationships among individual genomes, we utilized the bcftools (v.1.14 Danecek *et al.*, 2021) variant calling pipeline to produce a *vcf* file (--maf 0.05) and used it for the construction of a maximum likelihood tree with iqtree (v.2.2.2.2; Minh *et al.*, 2020). We used only variant sites in the nuclear tree generation and therefore used the ascertainment bias correction option (-m MFP+ASC) to correct the likelihood conditioned on variable sites. Branch support of the phylogenetic tree was evaluated by ultrafast bootstrap approximation with 1,000 bootstraps (-B 1000). The resulting *newick* tree was then midrooted and illustrated with the interactive tree of life (iTOL) webserver (Letunic & Bork, 2021). For comparison, the individual mitochondrial genomes from all samples were reconstructed to evaluate the matrilineal effect on geographic distribution. Briefly, coalqc (v.0.1; Patil *et al.*, 2020) was used to align California towhee reads to the *Zonotrichia albicollis* mitochondrial reference (accession number NC_053110.1) and the resulting mapped reads were interleaved and used as input for mitobim (v.1.8; Hahn *et al.*, 2013). Following mitochondrial genome reconstruction, multiple sequence alignment was performed using clustalx (v.2.0; Larkin *et al.*, 2007), and maximum likelihood tree was generated using iqtree (v.2.2.2.2). A chi-square goodness-of-fit test was used to determine if any samples had a higher number of observed gap/ambiguous sites in the multiple sequence alignment compared to that which is expected by chance; one sample (I_6) had a high percentage of gap/ambiguity sites and was removed. We bootstrapped the data 1,000 times (-B 1000) and HKY+F+I+R4 was chosen as the best-fit model according to the Bayesian Information Criterion (BIC). The resulting mitochondrial tree (n=80) was then midrooted and illustrated with the ITOL webserver.

Genetic differentiation (F_ST_) was calculated between each sampled region (OREG, CCAL, INYO, SCAL) using angsd. First, the site allele frequency likelihood was determined for each collection site (-doSaf 1). Secondly, the 2D site frequency spectrum for each pair of sampled regions was estimated (realSFS), from which the mean pairwise weighted F_ST_ values were obtained. Furthermore, non-overlapping sliding window analysis (50 kb, step = 25 kb) were performed for all geographic pairwise comparisons to identify outlier sequences (top 99.9%) potentially involved with local adaptation. As INYO was the primary focal population, genes within outlier windows involved in these pairwise comparisons were extracted with bcftools and subjected to gene ontology enrichment analysis (Mi *et al.*, 2019) with multiple testing correction, using *Gallus gallus* to determine potential biological function.

Further investigation of the outlier windows was performed using genotype-environment association test using redundancy analysis (RDA), following Capblancq & Forester (2021) and using R package “vegan” (Oksanen *et al*., 2025). We used the same environmental variables used for ecological niche modeling (see below) after centering and scaling. Population structure was controlled by incorporating geocoordinates in the RDA model since it corresponds with geography Model performance was evaluated with the adjusted R-squared value, and the significance of RDA axes was determined from 1000 permutation tests. Only significant RDA axis was used to identify environment-associated loci, whose loading scores are below or above 2 SD from mean loading on the axis.

**California towhee effective population size.** Patterns of linkage disequilibrium (LD) were used to estimate changes in *N_e_* over the past 100 generations using gone (Santiago *et al.,* 2020). Angsd was used to generate genotype likelihoods for each sampled geographic region, only retaining sites present in all individuals with ≥3x reads for each sample. Genotypes were then called from ~ 4M genotype likelihoods using beagle (Browning & Browning, 2016) and analysis was restricted to the twenty longest contigs (>20 Mb) with the following parameters: a constant recombination rate of 1.78 cM/Mb (Hagen *et al.,* 2020), a maximum number of 5K SNPs per contig over 400 bins, 2K number of generations for binning linkage data, and a recombination rate threshold (hc) of 0.05. The selected 5K SNPs from each contig were randomly picked for 500 repetitions. For each region, *N_e_* contemporary estimates (95% CI) were calculated using the LD method implemented in NeEstimator2 (Do *et al.,* 2014). For computational limitations, 50K SNPs were randomly selected from each geographic regions using the --thin command with plink (Purcell *et al.,* 2007). For interpreting historic *N_e_* plots, a generation time of 2 years was used (Brommer *et al.,* 2004; Nadachowska-Brzyska *et al.,* 2013).

**Ecological niche modeling.** California Towhee occurrence data were obtained from Global Biodiversity Information Facility (GBIF), an international data infrastructure that aggregates biodiversity occurrence records from worldwide institutions and museums (DOI: 10.15468/dl.pekmny). For the years 1980–2010, records classified as "Observation," "Human Observation," "Machine Observation," or "Occurrence" with coordinate uncertainty <200 meters (Benedict, 2009) were retained. Manual cleaning was conducted to remove points falling outside the known range, particularly north and east of Eugene, Oregon. Spatial thinning at 200 meters was applied to reduce sampling bias, resulting in 16,462 occurrence points. The study area was defined by creating a minimum convex hull around occurrences and buffering it by 200 km to capture the entire California. Six uncorrelated bioclimatic variables (BIO4, BIO9, BIO11, BIO12, BIO14, BIO18) were selected from PaleoClim (Brown *et al.,* 2018; Karger *et al.,* 2017) based on Pearson correlation analysis and clipped to the buffered extent. A Gaussian kernel density surface (0.25° smoothing) was generated to model sampling bias using SDMtoolbox (Brown *et al.,* 2017).

Modeling was conducted in R 4.2.2. First, an equal number of background points (n = 16,462) were randomly sampled (Barbet-Massin *et al.*, 2012) with bias weighting to further reduce sampling bias using R package “dismo” (Hijmans *et al*., 2024). Second, multiple algorithms (MaxEnt, CTA, XGBoost, MARS, GBM, GAM, ANN, RF) were compared using R package “Biomod2” (Guéguen *et al*., 2025), each evaluated with threefold random cross-validation (70% training, 30% testing) and ‘tuned’ optimization strategy. For MaxEnt, the optimal model was tuned using the “ENMeval” R package (Kass *et al.,* 2021), testing combinations of feature classes (L, LQ, LQH, LQHP, LQHPT, H), regularization multipliers (0.5–5.0 increasing by 0.5), and block cross-validation. Among all models tested, Random Forest achieved the highest predictive performance (TSS = 0.85, AUC = 0.98) and was used for ensemble modeling among the three iterations. Ensemble forecasts were produced by weighted averaging based on TSS scores. The final ensemble model was projected to mid-Holocene (Northgrippian; 8.326–4.2 ka), late-Holocene (Meghalayan; 4.2–0.3 ka), and the current period (1980–2010). Binary presence-absence maps were derived using TSS thresholds. Final outputs were visualized in ArcGIS Pro v3.4.

**Spatial patterns of gene flow.** To infer patterns of gene flow, we again used angsd to estimate genetic differentiation (pairwise F_ST_) but at the level of sampling sites (1–10; Figure 1B). For sampling locations containing ≤ 5 individuals, all samples (opposed to 80%) were required to process each polymorphic site for generation of the site frequency spectrum and calculation of mean weighted pairwise-F_ST_ values. These F_ST_ matrices were used with the landscape data in R package “ResistanceGA” (Peterman, 2018) to evaluate patterns of spatial gene flow influenced by landscape features.

Contemporary high resolution landscape raster data was collected from both USGS (30-m Digital Elevation Model (DEM); <https://www.usgs.gov/the-national-map-data-delivery>), National Land Cover Database 2021 (https://www.mrlc.gov/data), and AdaptWest (32 Bioclimatic variables with 1-km resolution; heetps://adaptwest.databasin.org). Topographic variables (elevation, aspect and slope) were derived from the DEM. We reclassified and reduced the original “Developed” landcover categories from four levels to two levels to be represented as categorial data in “ResistanceGA”. For matching spatial resolution among landscape data, a convex hull encompassing all the sampling sites was created and buffered by 20 km, the maximum natal dispersal distance of a related species (*Pipilo erythrophthalmus*, Eastern towhee; Tittler *et al.*, 2009). Annual heat moisture index (ahm), aspect, precipitation as snow (pas), summer precipitation (ppt_sm), mean annual relative humidity (rh), slope, and difference between mean temperature of the warmest month and mean temperature of the coldest month (td) were retained based on Pearson correlation analysis among landscape data. Detailed information about variable correlation can be found in Data S2. All landscape data preprocessing was conducted in ArcGIS Pro (v3.2; ESRI Inc.).

Landscape rasters from ArcGIS were further processed in R (R Core Team, 2021). Each raster was aggregated by a factor of 20 due to computational limitation using the R package “terra” (Hijmans, 2020), and NoData values were set as 10 * maximum value of the raster. We employed the R package “ResistanceGA” to optimize resistance surfaces for all individuals. The package also provides a Maximum Likelihood Population Effect (MLPE) coefficient that indicates the explanatory power of the optimized surface against genetic differentiation. Five different surface models were optimized: ‘habitat’ (slope, aspect, landcover), ‘climate’ (ahm, pas, ppt_sm, rh, td), ‘topography’ (slope, aspect), ‘landcover-only’, and ‘all-variable’ surfaces, considering different temporal scales of data from different sources (i.e., yearly basis for bioclimatic variables, decades for landcover, and geological time scale for topography). Optimized surfaces, including geographic distance as a null model, of all individuals were compared by average Akaike Information Criterion (AIC) from 1,000 iterations of bootstrapping.

Since the best surface model from all individuals was the distance model, implying stronger influence of geographic distance than landscape features tested, we employed Subsequent Fast Estimation of Effective Migration Surfaces (FEEMS; Marcus *et al.*, 2021) to estimate spatial gene flow patterns based on heterogeneity of isolation by distance. First, we generated plink format files among all individuals, all males, and all females to evaluate potential differences in gene flow due to sex. angsd was run as above (see section *Bioinformatic processing*) with the additional options applied for conservative genotype calling (-doPlink 2 -doGeno -4 -doPost 1 -doCounts 1 -postCutoff 0.99 -geno_minDepth 5). The three plink format files were separately input into the FEEMS for individual analyses. Outer coordinates of habitat ranges and the triangular grid file were generated in ArcGIS Pro. Sampling locations separated by more than 5 km were assigned to different grid cells. Following the developer’s workflow for overall FEEMS procedure (Marcus *et al.,* 2021; <https://github.com/NovembreLab/feems/blob/main/docsrc/notebooks/cross-validation.ipynb>, we tested 20 lambda values from 1e^-6^ to 1e^2^ (evenly spaced on a logarithmic scale) with the number of iterations equal to the number of observed nodes in the graph file generated by FEEMS. Lambda values were selected based on the lowest cross-validation error for each case.

**References**

Andrews, S. (2010). FASTQC: A quality control tool for high throughput sequence data. Retrieved from <http://www.bioinformatics.babraham.ac.uk/projects/fastqc/>

Barbet-Massin, M., Jiguet, F., Albert, C. H., & Thuiller, W. (2012). Selecting pseudo-absences for species distribution models: how, where and how many? Methods in Ecology and Evolution, 3, 327–338. <https://doi.org/10.1111/j.2041-210X.2011.00172.x>

Broad Institute. (2019). Picard-tools. Retrieved from <https://broadinstitute.github.io/picard/>

Brommer, J. E., Gustafsson, L., Pietiäinen, H., & Merilä, J. (2004). Single-generation estimates of individual fitness as proxies for long-term genetic contribution. American Naturalist, 163, 505–517.

Brown, J. L., Bennett, J. R., & French, C. M. (2017). SDMtoolbox 2.0: the next generation Python-based GIS toolkit for landscape genetic, biogeographic and species distribution model analyses. PeerJ, 5, e4095.

Browning, B. L., & Browning, S. R. (2016). Genotype imputation with millions of reference samples. American Journal of Human Genetics, 98, 116–126.

Challis, R., Richards, E., Rajan, J., Cochrane, G., & Blaxter, M. (2020). BlobToolKit — interactive quality assessment of genome assemblies. G3: Genes, Genomes, Genetics, 10, 1361–1374. https://doi.org/10.1534/g3.119.400908

Chen, S., Zhou, Y., Chen, Y., & Gu, J. (2018). fastp: an ultra-fast all-in-one FASTQ preprocessor. Bioinformatics, 34, i884–i890. https://doi.org/10.1093/bioinformatics/bty560

Cheng, H., Concepcion, G. T., Feng, X., Zhang, H., & Li, H. (2021). Haplotype-resolved de novo assembly using phased assembly graphs with hifiasm. Nature Methods, 18, 170–175. https://doi.org/10.1038/s41592-020-01056-5

Danecek, P., Bonfield, J. K., Liddle, J., Marshall, J., Ohan, V., Pollard, M. O., Whitwham, A., Keane, T., McCarthy, S. A., Davies, R. M., & Li, H. (2021). Twelve years of SAMtools and BCFtools. GigaScience, 10, giab008. https://doi.org/10.1093/gigascience/giab008

Do, C., Waples, R. S., Peel, D., Macbeth, G. M., Tillett, B. J., & Ovenden, J. R. (2014). NeEstimator v2: re-implementation of software for the estimation of contemporary effective population size (Ne) from genetic data. Molecular Ecology Resources, 14, 209–214.

Evanno, G., Regnaut, S., & Goudet, J. (2005). Detecting the number of clusters of individuals using the software STRUCTURE: a simulation study. Molecular Ecology, 14, 2611–2620. <https://doi.org/10.1111/j.1365-294X.2005.02553.x>

Francis, R. M. (2017). Pophelper: an R package and web app to analyse and visualize population structure. Molecular Ecology Resources, 17, 27–32. <https://doi.org/10.1111/1755-0998.12509>

Griffiths, R., Double, M. C., Orr, K., & Dawson, R. J. G. (1998). A DNA test to sex most birds. Molecular Ecology, 7, 1071–1075. https://doi.org/10.1046/j.1365-294x.1998.00389.x

Guan, D., McCarthy, S. A., Wood, J., Howe, K., Wang, Y., & Durbin, R. (2020). Identifying and removing haplotypic duplication in primary genome assemblies. Bioinformatics, 36, 2896–2898. https://doi.org/10.1093/bioinformatics/btaa025

Gurevich, A., Saveliev, V., Vyahhi, N., & Tesler, G. (2013). QUAST: quality assessment tool for genome assemblies. Bioinformatics, 29, 1072–1075. https://doi.org/10.1093/bioinformatics/btt086

Hagen, I. J., Lien, S., Billing, A. M., Elgvin, T. O., Trier, C., Niskanen, A. K., & Jensen, H. (2020). A genome-wide linkage map for the house sparrow (Passer domesticus) provides insights into the evolutionary history of the avian genome. Molecular Ecology Resources, 20, 544–559. https://doi.org/10.1111/1755-0998.13134

Hijmans, R. J. (2020). terra: Spatial data analysis. R package version 0.8-6. https://CRAN.R-project.org/package=terra

Hijmans, R., Phillips, S., Leathwick, J., & Elith, J. (2024). dismo: Species distribution modeling. R package version 1.3-16. https://CRAN.R-project.org/package=dismo

Jakobsson, M., & Rosenberg, N. A. (2007). CLUMPP: a cluster matching and permutation program for dealing with label switching and multimodality in analysis of population structure. Bioinformatics, 23, 1801–1806. https://doi.org/10.1093/bioinformatics/btm233

Kass, J., Muscarella, R., Galante, P., Bohl, C., Buitrago-Pinilla, G., Boria, R., Soley-Guardia, M., & Anderson, R. (2021). ENMeval 2.0: redesigned for customizable and reproducible modeling of species' niches and distributions. Methods in Ecology and Evolution, 12, 1602–1608.

Kopelman, N. M., Mayzel, J., Jakobsson, M., Rosenberg, N. A., & Mayrose, I. (2015). CLUMPAK: a program for identifying clustering modes and packaging population structure inferences across K. Molecular Ecology Resources, 15, 1179–1191. https://doi.org/10.1111/1755-0998.12387

Larkin, M. A., Blackshields, G., Brown, N. P., Chenna, R., McGettigan, P. A., McWilliam, H., Valentin, F., Wallace, I. M., Wilm, A., Lopez, R., Thompson, J. D., Gibson, T. J., & Higgins, D. G. (2007). Clustal W and Clustal X version 2.0. Bioinformatics, 23, 2947–2948. <https://doi.org/10.1093/bioinformatics/btm404>

Letunic, I., & Bork, P. (2021). Interactive Tree Of Life (iTOL) v5: an online tool for phylogenetic tree display and annotation. Nucleic Acids Research, 49, W293–W296. https://doi.org/10.1093/nar/gkab301

Li, H., Handsaker, B., Wysoker, A., Fennell, T., Ruan, J., Homer, N., Marth, G., Abecasis, G., & Durbin, R. (2009). The sequence alignment/map format and SAMtools. Bioinformatics, 25, 2078–2079. https://doi.org/10.1093/bioinformatics/btp352

McKenna, A., Hanna, M., Banks, E., Sivachenko, A., Cibulskis, K., Kernytsky, A., Garimella, K., Altshuler, D., Gabriel, S., Daly, M., & DePristo, M. A. (2010). The genome analysis toolkit: a MapReduce framework for analyzing next-generation DNA sequencing data. Genome Research, 20, 1297–1303. https://doi.org/10.1101/gr.107524.110

McQuillan, R., Leutenegger, A.-L., Abdel-Rahman, R., Franklin, C. S., Pericic, M., Barac-Lauc, L., Smolej-Narancic, N., Janicijevic, B., Polasek, O., Tenesa, A., MacLeod, A. K., Farrington, S. M., Rudan, P., Hayward, C., Vitart, V., Rudan, I., Wild, S. H., Dunlop, M. G., Wright, A. F., & Campbell, H. (2008). Runs of homozygosity in European populations. American Journal of Human Genetics, 83, 658. https://doi.org/10.1016/j.ajhg.2008.10.009

Mi, H., Muruganujan, A., Ebert, D., Huang, X., & Thomas, P. D. (2019). PANTHER version 14: more genomes, a new PANTHER GO-slim and improvements in enrichment analysis tools. Nucleic Acids Research, 47, D419–D426. https://doi.org/10.1093/nar/gky1038

Minh, B. Q., Schmidt, H. A., Chernomor, O., Schrempf, D., Woodhams, M. D., Von Haeseler, A., & Lanfear, R. (2020). IQ-TREE 2: new models and efficient methods for phylogenetic inference in the genomic era. Molecular Biology and Evolution, 37, 1530–1534. https://doi.org/10.1093/molbev/msaa015

Nadachowska-Brzyska, K., Burri, R., Olason, P. I., Kawakami, T., Smeds, L., & Ellegren, H. (2013). Demographic divergence history of pied flycatcher and collared flycatcher inferred from whole-genome resequencing data. PLoS Genetics, 9, e1003942.

Oksanen, J., Simpson, G. L., Blanchet, F. G., Kindt, R., Legendre, P., Minchin, P. R., O'Hara, P. R., Solymos, P., Stevens, M. H. H., Szoecs, E., Wagner, H., Barbour, M., Bedward, M., Bolker, B., Borcard, D., Borman, T., Carvalho, G., Chirico, M., De Caceres, M., Durand, S., Evangelista, H. B. A., FitzJohn, R., Friendly, M., Furneaux, B., Hannigan, G., Hill, M. O., Lahti, L., Martino, C., McGlinn, D., Ouellette, M.-H., Ribeiro Cunha, E., Smith, T., Stier, A., Ter Braak, C. J. F., & Weedon, J. (2025). vegan: community ecology package. R package version 2.8-0. <https://vegandevs.github.io/vegan/>

Ou, S., Su, W., Liao, Y., Chougule, K., Agda, J. R., Hellinga, A. J., & Hufford, M. B. (2019). Benchmarking transposable element annotation methods for creation of a streamlined, comprehensive pipeline. Genome Biology, 20, 1–18.

Patil, A. B., Shinde, S. S., Raghavendra, S., Satish, B. N., Kushalappa, C. G., & Vijay, N. (2020). CoalQC — quality control while inferring demographic histories from genomic data: application to forest tree genomes. bioRxiv [Preprint]. Available from: https://www.biorxiv.org/content/10.1101/2020.03.03.962365v1.full [Accessed 1 March 2022].

Pockrandt, C., Alzamel, M., Iliopoulos, C. S., & Reinert, K. (2020). GenMap: ultra-fast computation of genome mappability. Bioinformatics, 36, 3687–3692. https://doi.org/10.1093/bioinformatics/btaa222

Purcell, S., Neale, B., Todd-Brown, K., Thomas, L., Ferreira, M. A., Bender, D., & Sham, P. C. (2007). PLINK: a tool set for whole-genome association and population-based linkage analyses. American Journal of Human Genetics, 81, 559–575.

Santiago, E., Novo, I., Pardiñas, A. F., Saura, M., Wang, J., & Caballero, A. (2020). Recent demographic history inferred by high-resolution analysis of linkage disequilibrium. Molecular Biology and Evolution, 37, 3642–3653.

Seppey, M., Manni, M., & Zdobnov, E. M. (2019). BUSCO: assessing genome assembly and annotation completeness. In M. Kollmar (Ed.), Gene prediction: methods and protocols (pp. 227–245). Humana Press, Totowa, NJ.

Smit, A. F. A., Hubley, R. R., & Green, P. R. (2015). RepeatMasker Open-4.0. Retrieved from http://repeatmasker.org

Tittler, R., Villard, M.-A., & Fahrig, L. (2009). How far do songbirds disperse? Ecography, 32, 1051–1061. https://doi.org/10.1111/j.1600-0587.2009.05680.x

**Figure S1.** Linkage disequilibrium based historic effective population size (Ne) estimates for (A) Oregon (OREG), (B) Central California (CCAL), and (C) Southern California (SCAL) with 95% (light shading) and 50% (dark shading) confidence intervals among 500 iterations for the previous 100 generations. Note, the three sub-panels have different Y-axis scales.

**Figure S2.** *Fst sliding window analysis.* Non-overlapping sliding window analysis (50 kb) to illustrate differentiation (pairwise-F_ST_) between sampling regions (Oregon = OREG, INYO = Inyo County, CCAL = Central California, SCAL = Southern California). For the pairwise INYO comparisons, genes found within windows above the horizontal red dashed lines (> 99.9%) were used for Gene Ontology analysis.

**Figure S3.** Triplot of redundancy analysis. BIO9, BIO11, and BIO14 were the main drivers of environmental adaptation along RDA axis 1, while BIO12 was the primary driver along axis 2. Points are color-coded to represent loci significantly associated with corresponding bioclimatic variables. All bioclimatic variables used are shown as arrows, with four predictors of the environment-associated loci along the only statistically significant RDA axis (axis 1) highlighted in color. Axes 1 and 2 explained 49% and 18% of the variation, respectively, and the adjusted R-squared value of the model was 0.113.

**Figure S4.** Manhattan plot of loci included in the redundancy analysis with their loading scores on the RDA axis 1. Chromosomes containing loci are depicted, and points are color-coded according to their significance (below or above 2 SD from the mean).

**Figure S5.** Adaptive index distribution across the entire species range. The gradient colors represent spatial differentiation in local adaptation, where each pixel’s score is computed as the sum of standardized bioclim variables weighted by their loadings on the corresponding RDA axis.

**Figure S6.** Leave-One-Out Cross-validation results of (A) all individuals, (B) males only, (C) females over λ values in the range of (1e-6, 1e2). The plots show λ values on x-axis and cross-validation errors by L2 distance on y-axis.

**Table S1**. List of towhee (n=81) collected from ten different sampling sites. For phenotypic and genotypic sex assignment, U is unknown, M is male and F is female.

| **Sample ID** | **Species Code** | **Site** | **Population** | **Latitude** | **Longitude** | **Sex** | |
| --- | --- | --- | --- | --- | --- | --- | --- |
|  |  |  |  |  |  | Phenotypic | Genotypic |
| C_1 | CALT | 1 | OREG | 42.4843 | -122.892 | U | F |
| C_2 | CALT | 1 | OREG | 42.4843 | -122.892 | M | M |
| C_3 | CALT | 1 | OREG | 42.4843 | -122.892 | U | F |
| C_4 | CALT | 1 | OREG | 42.4843 | -122.892 | M | M |
| C_5 | CALT | 1 | OREG | 42.4843 | -122.892 | U | F |
| C_6 | CALT | 1 | OREG | 42.4843 | -122.892 | U | F |
| C_7 | CALT | 1 | OREG | 42.4843 | -122.892 | M | M |
| C_8 | CALT | 1 | OREG | 42.1375 | -122.616 | F | F |
| C_9 | CALT | 1 | OREG | 42.1375 | -122.616 | M | M |
| C_10 | CALT | 1 | OREG | 42.1375 | -122.616 | M | M |
| C_11 | CALT | 1 | OREG | 42.1375 | -122.616 | U | F |
| C_12 | CALT | 1 | OREG | 42.1375 | -122.616 | U | M |
| C_13 | CALT | 1 | OREG | 42.2097 | -122.735 | F | F |
| C_14 | CALT | 1 | OREG | 42.536 | -122.846 | U | M |
| C_15 | CALT | 10 | SCAL | 33.9855 | -117.313 | U | F |
| C_16 | CALT | 10 | SCAL | 33.9855 | -117.313 | U | F |
| C_17 | CALT | 9 | SCAL | 34.1092 | -117.712 | U | F |
| C_18 | CALT | 9 | SCAL | 34.1092 | -117.712 | U | F |
| C_19 | CALT | 9 | SCAL | 34.1092 | -117.712 | U | F |
| C_20 | CALT | 9 | SCAL | 34.1092 | -117.712 | U | F |
| C_21 | CALT | 9 | SCAL | 34.1092 | -117.712 | U | M |
| C_22 | CALT | 9 | SCAL | 34.1092 | -117.712 | U | F |
| C_23 | CALT | 9 | SCAL | 34.1092 | -117.712 | U | M |
| C_24 | CALT | 9 | SCAL | 34.1092 | -117.712 | U | F |
| C_25 | CALT | 9 | SCAL | 34.1092 | -117.712 | U | F |
| C_26 | CALT | 9 | SCAL | 34.1092 | -117.712 | U | F |
| C_27 | CALT | 9 | SCAL | 34.1092 | -117.712 | U | M |
| C_28 | CALT | 9 | SCAL | 34.1092 | -117.712 | U | F |
| C_29 | CALT | 9 | SCAL | 34.1092 | -117.712 | U | F |
| C_30 | CALT | 9 | SCAL | 34.1092 | -117.712 | U | M |
| C_31 | CALT | 9 | SCAL | 34.1092 | -117.712 | U | F |
| C_32 | CALT | 9 | SCAL | 34.1092 | -117.712 | U | F |
| C_33 | CALT | 9 | SCAL | 34.1092 | -117.712 | U | F |
| C_34 | CALT | 9 | SCAL | 34.1092 | -117.712 | U | F |
| C_35 | CALT | 9 | SCAL | 34.1092 | -117.712 | U | F |
| C_36 | CALT | 9 | SCAL | 34.1092 | -117.712 | U | F |
| C_37 | CALT | 9 | SCAL | 34.1092 | -117.712 | U | M |
| C_38 | CALT | 2 | CCAL | 38.1672 | -122.899 | U | M |
| C_39 | CALT | 2 | CCAL | 38.1672 | -122.899 | F | F |
| C_40 | CALT | 2 | CCAL | 38.1672 | -122.899 | U | M |
| C_41 | CALT | 2 | CCAL | 38.1672 | -122.899 | F | F |
| C-42 | CALT | 2 | CCAL | 38.2324 | -122.665 | M | M |
| C-43 | CALT | 2 | CCAL | 38.0999 | -122.864 | F | F |
| C-44 | CALT | 2 | CCAL | 38.0999 | -122.864 | M | M |
| C-45 | CALT | 3 | CCAL | 37.8975 | -122.71 | U | F |
| C-46 | CALT | 3 | CCAL | 37.8975 | -122.71 | U | F |
| C-47 | CALT | 3 | CCAL | 37.9077 | -122.703 | U | F |
| C-48 | CALT | 3 | CCAL | 37.9077 | -122.703 | U | M |
| C-49 | CALT | 3 | CCAL | 38.0145 | -122.67 | U | M |
| C-50 | CALT | 7 | CCAL | 37.0802 | -119.729 | M | M |
| C-51 | CALT | 7 | CCAL | 37.0802 | -119.729 | F | F |
| C-52 | CALT | 7 | CCAL | 37.0802 | -119.729 | U | F |
| C-53 | CALT | 7 | CCAL | 37.0802 | -119.729 | M | M |
| C-54 | CALT | 6 | CCAL | 37.457 | -119.792 | U | M |
| C-55 | CALT | 7 | CCAL | 37.0802 | -119.729 | F | F |
| C-56 | CALT | 7 | CCAL | 37.0802 | -119.729 | M | F |
| C-57 | CALT | 6 | CCAL | 37.457 | -119.792 | M | M |
| C-58 | CALT | 5 | CCAL | 37.7096 | -120.269 | M | M |
| C-59 | CALT | 5 | CCAL | 37.7096 | -120.269 | M | M |
| C-60 | CALT | 5 | CCAL | 37.7096 | -120.269 | F | F |
| C-61 | CALT | 5 | CCAL | 37.7096 | -120.269 | U | M |
| C_62 | CALT | 4 | CCAL | 37.4334 | -121.927 | U | F |
| C_63 | CALT | 4 | CCAL | 37.4373 | -121.927 | U | F |
| C_64 | CALT | 4 | CCAL | 37.4346 | -121.927 | U | F |
| C_65 | CALT | 4 | CCAL | 37.4336 | -121.929 | U | F |
| C_66 | CALT | 4 | CCAL | 37.4353 | -121.929 | U | F |
| C_67 | CALT | 4 | CCAL | 37.4374 | -121.928 | U | F |
| I_1 | ICTO | 8 | INYO | 35.8837 | -117.421 | M | M |
| I_2 | ICTO | 8 | INYO | 35.8837 | -117.421 | M | M |
| I_3 | ICTO | 8 | INYO | 35.8331 | -117.398 | F | F |
| I_4 | ICTO | 8 | INYO | 35.8331 | -117.398 | M | M |
| I_5 | ICTO | 8 | INYO | 35.8331 | -117.398 | F | F |
| I_6 | ICTO | 8 | INYO | 35.8837 | -117.421 | M | M |
| I_7 | ICTO | 8 | INYO | 35.8735 | -117.427 | F | F |
| I_8 | ICTO | 8 | INYO | 35.8735 | -117.427 | M | M |
| I_9 | ICTO | 8 | INYO | 35.8798 | -117.422 | M | M |
| I_10 | ICTO | 8 | INYO | 35.8798 | -117.422 | F | F |
| I_11 | ICTO | 8 | INYO | 35.8592 | -117.412 | M | F |
| I_12 | ICTO | 8 | INYO | 35.8592 | -117.412 | M | M |
| I_13 | ICTO | 8 | INYO | 35.8592 | -117.412 | U | F |
| I_14 | ICTO | 8 | INYO | 35.8592 | -117.412 | F | F |

**Table S2**. Assembly metrics from the Inyo California genome assembly.

| Scientific Name | *Melozone crissalis* |
| --- | --- |
| Common Name | California towhee |
| Accession | GCF_028551555.1 |
| Assembly level | Contig |
| Contigs (#) | 627 |
| N50 | 22,266,903 |
| L50 | 16 |
| Max Contig size | 89,094,009 |
| Total size (Gb) | 1.412 |
| GC content (%) | 44.2 |

**Table S3.** Summary of the transposable element (TE) repertoire for the California towhee reference genome generated with edta (Ou *et al.,* 2019).

| Class | Copies | bp | Percentage |
| --- | --- | --- | --- |
| *LTR* | | | |
| *Copia* | 1016 | 463930 | 0.03 |
| *Gypsy* | 50652 | 47320823 | 3.35 |
| *Unknown* | 105085 | 67470650 | 4.78 |
| *TIR* | | | |
| *CACTA* | 364416 | 143412293 | 10.16 |
| *Mutator* | 80849 | 31559747 | 2.23 |
| *PIF_Harbinger* | 5976 | 1934329 | 0.14 |
| *Tc1_Mariner* | 5903 | 2065691 | 0.15 |
| *hAT* | 7456 | 2223794 | 0.16 |
| *Non-LTR* | | | |
| *LINE_element* | 734 | 958162 | 0.07 |
| *Unknown* | 48 | 307189 | 0.02 |
| *Non-TIR* | | | |
| *Helitron* | 16584 | 5723223 | 0.41 |
| *Unspecified repeat region* | | | |
|  | 152041 | 108830139 | 7.71 |
| Total | **790760** | **412269970** | **29.19** |

**Table S4.** Passerine genomes and associated Sequence Read Archive (SRA) files used to contextualize California towhee diversity metrics. The species of the associated SRA file, the N50 of the downloaded (FTP) genome, and SRA ID are listed for each sample. For each sample, Individual Heterozygosity and the frequency of Runs Of Homozygosity are reported, in addition to alignment statistics (Breadth, Depth, and Mapping %). The Table contains the unfiltered list. Individuals rows were removed based upon minimum N50 (>22Mb) and species sample size (≥2) prior to calculating / retaining final fROH values.

| **Species** | **N50(MB)** | **FTP** | **SRA/SampleID** | **Heterozygosity** | **fROH(100KB-1MB)** | **fROH(>1MB)** | **fROH(total)** | **Breadth** | **Depth** | **Mapping(%)** |
| --- | --- | --- | --- | --- | --- | --- | --- | --- | --- | --- |
| *Agelaius phoeniceus* | 36.4 | <https://ftp.ncbi.nlm.nih.gov/genomes/all/GCF/020/745/825/GCF_020745825.1_Agelaius_phoeniceus_1.1/> | SRR9853697 | 0.00945106 | 0.0000000 | 0.000000 | 0.0000000 | 98.49 | 76.64 | 97.25 |
| *Ammospiza caudacuta* | 78.4 | <https://ftp.ncbi.nlm.nih.gov/genomes/all/GCF/027/887/145/GCF_027887145.1_bAmmCau1.pri/> | SRR15110330 | 0.00215789 | 0.0013683 | 0.000000 | 0.0013683 | 89.60 | 3.31 | 98.73 |
| *Ammospiza caudacuta* | 78.4 | https://ftp.ncbi.nlm.nih.gov/genomes/all/GCF/027/887/145/GCF_027887145.1_bAmmCau1.pri/ | SRR28359879 | 0.00187740 | 0.0333532 | 0.000891 | 0.0342445 | 92.49 | 4.11 | 98.84 |
| *Ammospiza caudacuta* | 78.4 | https://ftp.ncbi.nlm.nih.gov/genomes/all/GCF/027/887/145/GCF_027887145.1_bAmmCau1.pri/ | SRR15110329 | 0.00139776 | 0.0128730 | 0.002094 | 0.0149674 | 95.86 | 4.90 | 99.67 |
| *Ammospiza caudacuta* | 78.4 | https://ftp.ncbi.nlm.nih.gov/genomes/all/GCF/027/887/145/GCF_027887145.1_bAmmCau1.pri/ | SRR28359880 | 0.00101950 | 0.0113195 | 0.000000 | 0.0113195 | 96.28 | 5.69 | 99.62 |
| *Ammospiza caudacuta* | 78.4 | https://ftp.ncbi.nlm.nih.gov/genomes/all/GCF/027/887/145/GCF_027887145.1_bAmmCau1.pri/ | SRR15110327 | 0.00114369 | 0.0069473 | 0.000000 | 0.0069473 | 97.45 | 6.88 | 99.64 |
| *Ammospiza caudacuta* | 78.4 | https://ftp.ncbi.nlm.nih.gov/genomes/all/GCF/027/887/145/GCF_027887145.1_bAmmCau1.pri/ | SRR15110328 | 0.00259880 | 0.0053881 | 0.000884 | 0.0062717 | 85.99 | 2.61 | 98.92 |
| *Ammospiza caudacuta* | 78.4 | https://ftp.ncbi.nlm.nih.gov/genomes/all/GCF/027/887/145/GCF_027887145.1_bAmmCau1.pri/ | SRR15110332 | 0.00175423 | 0.0048152 | 0.000000 | 0.0048152 | 95.19 | 4.21 | 99.36 |
| *Ammospiza caudacuta* | 78.4 | https://ftp.ncbi.nlm.nih.gov/genomes/all/GCF/027/887/145/GCF_027887145.1_bAmmCau1.pri/ | SRR28359878 | 0.00173412 | 0.0306550 | 0.020653 | 0.0513081 | 93.39 | 3.75 | 99.19 |
| *Ammospiza caudacuta* | 78.4 | https://ftp.ncbi.nlm.nih.gov/genomes/all/GCF/027/887/145/GCF_027887145.1_bAmmCau1.pri/ | SRR15110333 | 0.00272589 | 0.0084296 | 0.000000 | 0.0084296 | 89.71 | 2.89 | 99.09 |
| *Ammospiza caudacuta* | 78.4 | https://ftp.ncbi.nlm.nih.gov/genomes/all/GCF/027/887/145/GCF_027887145.1_bAmmCau1.pri/ | SRR28359871 | 0.00246257 | 0.0144569 | 0.002941 | 0.0173979 | 94.10 | 3.83 | 99.32 |
| *Ammospiza caudacuta* | 78.4 | https://ftp.ncbi.nlm.nih.gov/genomes/all/GCF/027/887/145/GCF_027887145.1_bAmmCau1.pri/ | SRR28359883 | 0.00158823 | 0.0059933 | 0.000823 | 0.0059933 | 94.06 | 4.26 | 99.26 |
| *Ammospiza caudacuta* | 78.4 | https://ftp.ncbi.nlm.nih.gov/genomes/all/GCF/027/887/145/GCF_027887145.1_bAmmCau1.pri/ | SRR15110326 | 0.00112836 | 0.0071149 | 0.000853 | 0.0071149 | 96.78 | 6.04 | 99.65 |
| *Ammospiza caudacuta* | 78.4 | https://ftp.ncbi.nlm.nih.gov/genomes/all/GCF/027/887/145/GCF_027887145.1_bAmmCau1.pri/ | SRR28359884 | 0.00124601 | 0.0296664 | 0.002944 | 0.0326101 | 96.52 | 5.56 | 99.61 |
| *Ammospiza caudacuta* | 78.4 | https://ftp.ncbi.nlm.nih.gov/genomes/all/GCF/027/887/145/GCF_027887145.1_bAmmCau1.pri/ | SRR28359881 | 0.00367020 | 0.0175234 | 0.001923 | 0.0194464 | 90.15 | 3.06 | 99.22 |
| *Ammospiza caudacuta* | 78.4 | https://ftp.ncbi.nlm.nih.gov/genomes/all/GCF/027/887/145/GCF_027887145.1_bAmmCau1.pri/ | SRR28359870 | 0.00404688 | 0.0077714 | 0.000000 | 0.0077714 | 70.38 | 1.51 | 98.65 |
| *Ammospiza caudacuta* | 78.4 | https://ftp.ncbi.nlm.nih.gov/genomes/all/GCF/027/887/145/GCF_027887145.1_bAmmCau1.pri/ | SRR15110331 | 0.00176461 | 0.0064930 | 0.000000 | 0.0064930 | 93.26 | 3.66 | 99.31 |
| *Ammospiza caudacuta* | 78.4 | https://ftp.ncbi.nlm.nih.gov/genomes/all/GCF/027/887/145/GCF_027887145.1_bAmmCau1.pri/ | SRR28359882 | 0.00208281 | 0.0084674 | 0.000000 | 0.0084674 | 92.47 | 3.42 | 99.34 |
| *Ammospiza maritima* | 67.8 | <https://ftp.ncbi.nlm.nih.gov/genomes/all/GCA/036/010/785/GCA_036010785.1_bAmmMar1.pri/> | SRR15110306 | 0.00214482 | 0.0079755 | 0.000000 | 0.0079755 | 92.66 | 3.67 | 99.40 |
| *Ammospiza maritima* | 67.8 | https://ftp.ncbi.nlm.nih.gov/genomes/all/GCA/036/010/785/GCA_036010785.1_bAmmMar1.pri/ | SRR15110301 | 0.00223549 | 0.0076819 | 0.001000 | 0.0086819 | 94.45 | 4.15 | 99.40 |
| *Ammospiza maritima* | 67.8 | https://ftp.ncbi.nlm.nih.gov/genomes/all/GCA/036/010/785/GCA_036010785.1_bAmmMar1.pri/ | SRR15110305 | 0.00215769 | 0.0080678 | 0.000000 | 0.0080678 | 89.92 | 2.99 | 99.20 |
| *Ammospiza maritima* | 67.8 | https://ftp.ncbi.nlm.nih.gov/genomes/all/GCA/036/010/785/GCA_036010785.1_bAmmMar1.pri/ | SRR15110300 | 0.00219012 | 0.0102878 | 0.000818 | 0.0111062 | 90.37 | 3.03 | 99.40 |
| *Ammospiza maritima* | 67.8 | https://ftp.ncbi.nlm.nih.gov/genomes/all/GCA/036/010/785/GCA_036010785.1_bAmmMar1.pri/ | SRR15110307 | 0.00210647 | 0.0050973 | 0.000000 | 0.0050973 | 93.39 | 3.81 | 99.50 |
| *Ammospiza maritima* | 67.8 | https://ftp.ncbi.nlm.nih.gov/genomes/all/GCA/036/010/785/GCA_036010785.1_bAmmMar1.pri/ | SRR15110304 | 0.00210437 | 0.0067480 | 0.000000 | 0.0067480 | 97.19 | 6.51 | 99.00 |
| *Ammospiza maritima* | 67.8 | https://ftp.ncbi.nlm.nih.gov/genomes/all/GCA/036/010/785/GCA_036010785.1_bAmmMar1.pri/ | SRR15110302 | 0.00226454 | 0.0064480 | 0.000000 | 0.0064480 | 92.32 | 3.48 | 99.30 |
| *Ammospiza maritima* | 67.8 | https://ftp.ncbi.nlm.nih.gov/genomes/all/GCA/036/010/785/GCA_036010785.1_bAmmMar1.pri/ | SRR15110309 | 0.00226812 | 0.0137317 | 0.004209 | 0.0179404 | 79.39 | 1.89 | 99.10 |
| *Ammospiza maritima* | 67.8 | https://ftp.ncbi.nlm.nih.gov/genomes/all/GCA/036/010/785/GCA_036010785.1_bAmmMar1.pri/ | SRR15110310 | 0.00225921 | 0.0071551 | 0.000000 | 0.0071551 | 89.09 | 2.89 | 99.30 |
| *Ammospiza maritima* | 67.8 | https://ftp.ncbi.nlm.nih.gov/genomes/all/GCA/036/010/785/GCA_036010785.1_bAmmMar1.pri/ | SRR15110308 | 0.00237202 | 0.0064547 | 0.000000 | 0.0064547 | 86.11 | 2.46 | 99.20 |
| *Ammospiza nelsoni* | 74.7 | <https://ftp.ncbi.nlm.nih.gov/genomes/all/GCF/027/579/445/GCF_027579445.1_bAmmNel1.pri/> | SRR28359877 | 0.00153280 | 0.0333437 | 0.006198 | 0.0395420 | 95.42 | 4.96 | 99.11 |
| *Ammospiza nelsoni* | 74.7 | <https://ftp.ncbi.nlm.nih.gov/genomes/all/GCF/027/579/445/GCF_027579445.1_bAmmNel1.pri/> | SRR28359872 | 0.00149057 | 0.0902524 | 0.042853 | 0.1331051 | 89.32 | 2.92 | 98.52 |
| *Ammospiza nelsoni* | 74.7 | <https://ftp.ncbi.nlm.nih.gov/genomes/all/GCF/027/579/445/GCF_027579445.1_bAmmNel1.pri/> | SRR28359885 | 0.00418838 | 0.0125626 | 0.000000 | 0.0125626 | 84.25 | 2.48 | 98.44 |
| *Ammospiza nelsoni* | 74.7 | <https://ftp.ncbi.nlm.nih.gov/genomes/all/GCF/027/579/445/GCF_027579445.1_bAmmNel1.pri/> | SRR28359876 | 0.00285549 | 0.0295371 | 0.000000 | 0.0295371 | 92.02 | 3.55 | 98.66 |
| *Ammospiza nelsoni* | 74.7 | <https://ftp.ncbi.nlm.nih.gov/genomes/all/GCF/027/579/445/GCF_027579445.1_bAmmNel1.pri/> | SRR28359875 | 0.00217414 | 0.0209916 | 0.002793 | 0.0237843 | 92.76 | 3.79 | 98.45 |
| *Ammospiza nelsoni* | 74.7 | <https://ftp.ncbi.nlm.nih.gov/genomes/all/GCF/027/579/445/GCF_027579445.1_bAmmNel1.pri/> | SRR28359886 | 0.00194053 | 0.0197974 | 0.000000 | 0.0197974 | 96.56 | 6.02 | 98.26 |
| *Ammospiza nelsoni* | 74.7 | <https://ftp.ncbi.nlm.nih.gov/genomes/all/GCF/027/579/445/GCF_027579445.1_bAmmNel1.pri/> | SRR28359873 | 0.00229217 | 0.0119089 | 0.001166 | 0.0130748 | 93.22 | 3.95 | 98.43 |
| *Ammospiza nelsoni* | 74.7 | <https://ftp.ncbi.nlm.nih.gov/genomes/all/GCF/027/579/445/GCF_027579445.1_bAmmNel1.pri/> | SRR28359874 | 0.00318827 | 0.0190284 | 0.003135 | 0.0221633 | 87.34 | 2.95 | 98.55 |
| [*Ammospiza nelsoni nelsoni*](https://www.ncbi.nlm.nih.gov/Taxonomy/Browser/wwwtax.cgi?mode=Info&id=2857395) | 74.7 | <https://ftp.ncbi.nlm.nih.gov/genomes/all/GCF/027/579/445/GCF_027579445.1_bAmmNel1.pri/> | SRR15110341 | 0.00224155 | 0.0010583 | 0.000000 | 0.0010583 | 94.40 | 4.34 | 99.00 |
| [*Ammospiza nelsoni nelsoni*](https://www.ncbi.nlm.nih.gov/Taxonomy/Browser/wwwtax.cgi?mode=Info&id=2857395) | 74.7 | <https://ftp.ncbi.nlm.nih.gov/genomes/all/GCF/027/579/445/GCF_027579445.1_bAmmNel1.pri/> | SRR15110344 | 0.00197843 | 0.0007622 | 0.000000 | 0.0007622 | 89.75 | 3.13 | 99.08 |
| [*Ammospiza nelsoni nelsoni*](https://www.ncbi.nlm.nih.gov/Taxonomy/Browser/wwwtax.cgi?mode=Info&id=2857395) | 74.7 | <https://ftp.ncbi.nlm.nih.gov/genomes/all/GCF/027/579/445/GCF_027579445.1_bAmmNel1.pri/> | SRR15110342 | 0.00354375 | 0.0008343 | 0.000000 | 0.0008343 | 95.23 | 5.00 | 98.61 |
| [*Ammospiza nelsoni nelsoni*](https://www.ncbi.nlm.nih.gov/Taxonomy/Browser/wwwtax.cgi?mode=Info&id=2857395) | 74.7 | <https://ftp.ncbi.nlm.nih.gov/genomes/all/GCF/027/579/445/GCF_027579445.1_bAmmNel1.pri/> | SRR15110296 | 0.00475090 | 0.0006266 | 0.000000 | 0.0006266 | 93.63 | 4.18 | 98.87 |
| [*Ammospiza nelsoni nelsoni*](https://www.ncbi.nlm.nih.gov/Taxonomy/Browser/wwwtax.cgi?mode=Info&id=2857395) | 74.7 | <https://ftp.ncbi.nlm.nih.gov/genomes/all/GCF/027/579/445/GCF_027579445.1_bAmmNel1.pri/> | SRR15110343 | 0.00574293 | 0.0006185 | 0.000000 | 0.0006185 | 91.16 | 3.35 | 98.23 |
| [*Ammospiza nelsoni nelsoni*](https://www.ncbi.nlm.nih.gov/Taxonomy/Browser/wwwtax.cgi?mode=Info&id=2857395) | 74.7 | <https://ftp.ncbi.nlm.nih.gov/genomes/all/GCF/027/579/445/GCF_027579445.1_bAmmNel1.pri/> | SRR15110345 | 0.00562034 | 0.0000000 | 0.000000 | 0.0000000 | 94.52 | 4.53 | 98.45 |
| [*Ammospiza nelsoni nelsoni*](https://www.ncbi.nlm.nih.gov/Taxonomy/Browser/wwwtax.cgi?mode=Info&id=2857395) | 74.7 | <https://ftp.ncbi.nlm.nih.gov/genomes/all/GCF/027/579/445/GCF_027579445.1_bAmmNel1.pri/> | SRR15110346 | 0.00305342 | 0.0004801 | 0.000000 | 0.0004801 | 91.85 | 3.53 | 98.97 |
| [*Ammospiza nelsoni nelsoni*](https://www.ncbi.nlm.nih.gov/Taxonomy/Browser/wwwtax.cgi?mode=Info&id=2857395) | 74.7 | <https://ftp.ncbi.nlm.nih.gov/genomes/all/GCF/027/579/445/GCF_027579445.1_bAmmNel1.pri/> | SRR15110297 | 0.00271729 | 0.0003747 | 0.000000 | 0.0003747 | 91.33 | 3.38 | 98.77 |
| *Aphelocoma coerulescens* | 68 | <https://ftp.ncbi.nlm.nih.gov/genomes/all/GCA/041/296/385/GCA_041296385.1_UR_Acoe_1.0/> | SRR9853756 | 0.00202309 | 0.1047618 | 0.004942 | 0.1097042 | 98.05 | 9.56 | 99.78 |
| *Aphelocoma coerulescens* | 68 | https://ftp.ncbi.nlm.nih.gov/genomes/all/GCA/041/296/385/GCA_041296385.1_UR_Acoe_1.0/ | SRR28640760 | 0.00197249 | 0.0860997 | 0.009811 | 0.0959104 | 95.41 | 9.11 | 99.69 |
| *Aphelocoma coerulescens* | 68 | https://ftp.ncbi.nlm.nih.gov/genomes/all/GCA/041/296/385/GCA_041296385.1_UR_Acoe_1.0/ | SRR28640759 | 0.00197108 | 0.1295530 | 0.017428 | 0.1469805 | 98.31 | 10.34 | 99.79 |
| *Aphelocoma coerulescens* | 68 | https://ftp.ncbi.nlm.nih.gov/genomes/all/GCA/041/296/385/GCA_041296385.1_UR_Acoe_1.0/ | SRR28640748 | 0.00210251 | 0.0713830 | 0.013445 | 0.0848280 | 95.70 | 10.60 | 99.67 |
| *Aphelocoma coerulescens* | 68 | https://ftp.ncbi.nlm.nih.gov/genomes/all/GCA/041/296/385/GCA_041296385.1_UR_Acoe_1.0/ | SRR28640737 | 0.00204602 | 0.1098488 | 0.009848 | 0.1196969 | 98.66 | 11.27 | 99.70 |
| *Aphelocoma coerulescens* | 68 | https://ftp.ncbi.nlm.nih.gov/genomes/all/GCA/041/296/385/GCA_041296385.1_UR_Acoe_1.0/ | SRR28640726 | 0.00208533 | 0.0898789 | 0.016145 | 0.1060235 | 98.14 | 8.81 | 99.77 |
| *Aphelocoma coerulescens* | 68 | https://ftp.ncbi.nlm.nih.gov/genomes/all/GCA/041/296/385/GCA_041296385.1_UR_Acoe_1.0/ | SRR28640715 | 0.00208153 | 0.0961578 | 0.011358 | 0.1075154 | 98.89 | 12.34 | 99.70 |
| *Aphelocoma coerulescens* | 68 | https://ftp.ncbi.nlm.nih.gov/genomes/all/GCA/041/296/385/GCA_041296385.1_UR_Acoe_1.0/ | SRR28640704 | 0.00222358 | 0.0510788 | 0.000780 | 0.0518589 | 97.76 | 8.96 | 99.78 |
| *Aphelocoma coerulescens* | 68 | https://ftp.ncbi.nlm.nih.gov/genomes/all/GCA/041/296/385/GCA_041296385.1_UR_Acoe_1.0/ | SRR28640693 | 0.00207554 | 0.0672783 | 0.024276 | 0.0915542 | 95.44 | 9.71 | 99.66 |
| *Aphelocoma coerulescens* | 68 | https://ftp.ncbi.nlm.nih.gov/genomes/all/GCA/041/296/385/GCA_041296385.1_UR_Acoe_1.0/ | SRR28640682 | 0.00198826 | 0.0715694 | 0.014138 | 0.0857069 | 95.25 | 9.05 | 99.67 |
| *Aphelocoma coerulescens* | 68 | https://ftp.ncbi.nlm.nih.gov/genomes/all/GCA/041/296/385/GCA_041296385.1_UR_Acoe_1.0/ | SRR28640674 | 0.00190969 | 0.1074840 | 0.017714 | 0.1251984 | 95.27 | 9.62 | 99.67 |
| *Aphelocoma coerulescens* | 68 | https://ftp.ncbi.nlm.nih.gov/genomes/all/GCA/041/296/385/GCA_041296385.1_UR_Acoe_1.0/ | SRR28640758 | 0.00201450 | 0.0873937 | 0.010556 | 0.0979493 | 97.67 | 9.09 | 99.78 |
| *Aphelocoma coerulescens* | 68 | https://ftp.ncbi.nlm.nih.gov/genomes/all/GCA/041/296/385/GCA_041296385.1_UR_Acoe_1.0/ | SRR28640757 | 0.00195379 | 0.0934180 | 0.022454 | 0.1158721 | 95.17 | 9.30 | 99.68 |
| *Aphelocoma coerulescens* | 68 | https://ftp.ncbi.nlm.nih.gov/genomes/all/GCA/041/296/385/GCA_041296385.1_UR_Acoe_1.0/ | SRR28640756 | 0.00204648 | 0.0649834 | 0.017134 | 0.0821173 | 95.23 | 9.06 | 99.66 |
| *Aphelocoma coerulescens* | 68 | https://ftp.ncbi.nlm.nih.gov/genomes/all/GCA/041/296/385/GCA_041296385.1_UR_Acoe_1.0/ | SRR28640755 | 0.00210129 | 0.0878496 | 0.014257 | 0.1021065 | 98.38 | 11.90 | 99.70 |
| *Aphelocoma coerulescens* | 68 | https://ftp.ncbi.nlm.nih.gov/genomes/all/GCA/041/296/385/GCA_041296385.1_UR_Acoe_1.0/ | SRR28640754 | 0.00200882 | 0.0895318 | 0.005978 | 0.0955096 | 97.63 | 9.24 | 99.76 |
| *Aphelocoma coerulescens* | 68 | https://ftp.ncbi.nlm.nih.gov/genomes/all/GCA/041/296/385/GCA_041296385.1_UR_Acoe_1.0/ | SRR28640753 | 0.00203842 | 0.0919340 | 0.013750 | 0.1056838 | 98.17 | 9.26 | 99.78 |
| *Aphelocoma coerulescens* | 68 | https://ftp.ncbi.nlm.nih.gov/genomes/all/GCA/041/296/385/GCA_041296385.1_UR_Acoe_1.0/ | SRR28640752 | 0.00215763 | 0.0348713 | 0.011582 | 0.0464535 | 95.42 | 10.01 | 99.68 |
| *Aphelocoma coerulescens* | 68 | https://ftp.ncbi.nlm.nih.gov/genomes/all/GCA/041/296/385/GCA_041296385.1_UR_Acoe_1.0/ | SRR28640751 | 0.00211284 | 0.0802227 | 0.003881 | 0.0841034 | 98.06 | 9.95 | 99.78 |
| *Aphelocoma coerulescens* | 68 | https://ftp.ncbi.nlm.nih.gov/genomes/all/GCA/041/296/385/GCA_041296385.1_UR_Acoe_1.0/ | SRR28640750 | 0.00195266 | 0.0810874 | 0.011562 | 0.0926497 | 95.39 | 8.77 | 99.68 |
| *Aphelocoma coerulescens* | 68 | https://ftp.ncbi.nlm.nih.gov/genomes/all/GCA/041/296/385/GCA_041296385.1_UR_Acoe_1.0/ | SRR28640749 | 0.00207153 | 0.0598471 | 0.011526 | 0.0713733 | 95.37 | 9.02 | 99.68 |
| *Aphelocoma coerulescens* | 68 | https://ftp.ncbi.nlm.nih.gov/genomes/all/GCA/041/296/385/GCA_041296385.1_UR_Acoe_1.0/ | SRR28640747 | 0.00200226 | 0.0768295 | 0.010205 | 0.0870343 | 95.20 | 9.26 | 99.65 |
| *Aphelocoma coerulescens* | 68 | https://ftp.ncbi.nlm.nih.gov/genomes/all/GCA/041/296/385/GCA_041296385.1_UR_Acoe_1.0/ | SRR28640746 | 0.00202552 | 0.0907695 | 0.005000 | 0.0957693 | 97.70 | 9.29 | 99.77 |
| *Aphelocoma coerulescens* | 68 | https://ftp.ncbi.nlm.nih.gov/genomes/all/GCA/041/296/385/GCA_041296385.1_UR_Acoe_1.0/ | SRR28640745 | 0.00194578 | 0.1173706 | 0.006475 | 0.1238451 | 97.83 | 9.17 | 99.78 |
| *Aphelocoma coerulescens* | 68 | https://ftp.ncbi.nlm.nih.gov/genomes/all/GCA/041/296/385/GCA_041296385.1_UR_Acoe_1.0/ | SRR28640744 | 0.00209064 | 0.0931464 | 0.023930 | 0.1170766 | 98.05 | 9.26 | 99.76 |
| *Aphelocoma coerulescens* | 68 | https://ftp.ncbi.nlm.nih.gov/genomes/all/GCA/041/296/385/GCA_041296385.1_UR_Acoe_1.0/ | SRR28640743 | 0.00193009 | 0.1297490 | 0.015436 | 0.1451854 | 97.83 | 9.24 | 99.79 |
| *Aphelocoma coerulescens* | 68 | https://ftp.ncbi.nlm.nih.gov/genomes/all/GCA/041/296/385/GCA_041296385.1_UR_Acoe_1.0/ | SRR28640742 | 0.00208468 | 0.0764815 | 0.012550 | 0.0890310 | 97.92 | 9.24 | 99.77 |
| *Aphelocoma coerulescens* | 68 | https://ftp.ncbi.nlm.nih.gov/genomes/all/GCA/041/296/385/GCA_041296385.1_UR_Acoe_1.0/ | SRR28640741 | 0.00203761 | 0.0753313 | 0.013126 | 0.0884568 | 97.95 | 9.42 | 99.79 |
| *Aphelocoma coerulescens* | 68 | https://ftp.ncbi.nlm.nih.gov/genomes/all/GCA/041/296/385/GCA_041296385.1_UR_Acoe_1.0/ | SRR28640740 | 0.00173331 | 0.1880309 | 0.033588 | 0.2216187 | 98.26 | 10.09 | 99.78 |
| *Aphelocoma coerulescens* | 68 | https://ftp.ncbi.nlm.nih.gov/genomes/all/GCA/041/296/385/GCA_041296385.1_UR_Acoe_1.0/ | SRR28640739 | 0.00212280 | 0.1767557 | 0.056155 | 0.2329108 | 65.84 | 35.08 | 99.20 |
| *Bombycilla garrulus* | 0.6151 | <https://ftp.ncbi.nlm.nih.gov/genomes/all/GCA/013/400/315/GCA_013400315.1_ASM1340031v1/> | SRR9947014 | 0.00608944 | 0.0000000 | 0.000000 | 0.0000000 | 97.87 | 9.07 | 94.88 |
| *Calcarius ornatus* | 1.2 | <https://ftp.ncbi.nlm.nih.gov/genomes/all/GCA/013/397/715/GCA_013397715.1_ASM1339771v1/> | SRR9946933 | 0.01018341 | 0.0000000 | 0.000000 | 0.0000000 | 93.71 | 7.78 | 89.14 |
| *Cardinalis cardinalis* | 3.6 | <https://ftp.ncbi.nlm.nih.gov/genomes/all/GCA/014/549/065/GCA_014549065.1_CCar_1.0/> | SRR10019929 | 0.00548128 | 0.0000000 | 0.000000 | 0.0000000 | 96.74 | 10.58 | 96.31 |
| *Catharus bicknelli* | 0.982 | <https://ftp.ncbi.nlm.nih.gov/genomes/all/GCA/021/437/205/GCA_021437205.1_HU_Cbicknelli_1.0/> | SRR15809671 | 0.00844155 | 0.0002190 | 0.000000 | 0.0002190 | 93.17 | 10.81 | 95.91 |
| *Catharus bicknelli* | 0.982 | <https://ftp.ncbi.nlm.nih.gov/genomes/all/GCA/021/437/205/GCA_021437205.1_HU_Cbicknelli_1.0/> | SRR15809674 | 0.03591105 | 0.0000000 | 0.000000 | 0.0000000 | 93.74 | 43.36 | 68.90 |
| *Catharus bicknelli* | 0.982 | <https://ftp.ncbi.nlm.nih.gov/genomes/all/GCA/021/437/205/GCA_021437205.1_HU_Cbicknelli_1.0/> | SRR15809693 | 0.00803985 | 0.0024510 | 0.000000 | 0.0024510 | 93.55 | 16.47 | 95.30 |
| *Catharus bicknelli* | 0.982 | <https://ftp.ncbi.nlm.nih.gov/genomes/all/GCA/021/437/205/GCA_021437205.1_HU_Cbicknelli_1.0/> | SRR15809673 | 0.00899061 | 0.0000000 | 0.000000 | 0.0000000 | 93.49 | 18.66 | 97.50 |
| *Catharus bicknelli* | 0.982 | <https://ftp.ncbi.nlm.nih.gov/genomes/all/GCA/021/437/205/GCA_021437205.1_HU_Cbicknelli_1.0/> | SRR15809675 | 0.00847739 | 0.0012020 | 0.000000 | 0.0012020 | 93.51 | 16.66 | 96.60 |
| *Catharus bicknelli* | 0.982 | <https://ftp.ncbi.nlm.nih.gov/genomes/all/GCA/021/437/205/GCA_021437205.1_HU_Cbicknelli_1.0/> | SRR15809676 | 0.00882442 | 9.7543850 | 0.000000 | 9.7543850 | 93.47 | 15.88 | 97.00 |
| *Catharus bicknelli* | 0.982 | <https://ftp.ncbi.nlm.nih.gov/genomes/all/GCA/021/437/205/GCA_021437205.1_HU_Cbicknelli_1.0/> | SRR15809677 | 0.00843686 | 0.0015500 | 0.000000 | 0.0015500 | 93.42 | 16.17 | 96.40 |
| *Catharus bicknelli* | 0.982 | <https://ftp.ncbi.nlm.nih.gov/genomes/all/GCA/021/437/205/GCA_021437205.1_HU_Cbicknelli_1.0/> | SRR15809682 | 0.00825022 | 0.0036620 | 0.000000 | 0.0036620 | 93.37 | 12.50 | 96.01 |
| *Catharus bicknelli* | 0.982 | <https://ftp.ncbi.nlm.nih.gov/genomes/all/GCA/021/437/205/GCA_021437205.1_HU_Cbicknelli_1.0/> | SRR15809670 | 0.00834190 | 0.0059210 | 0.000000 | 0.0059210 | 93.51 | 17.22 | 96.30 |
| *Catharus fuscescens* | 0.276 | <https://ftp.ncbi.nlm.nih.gov/genomes/all/GCA/013/398/975/GCA_013398975.1_ASM1339897v1/> | SRR9946618 | 0.01038975 | 0.0000000 | 0.000000 | 0.0000000 | 98.60 | 12.66 | 95.36 |
| *Catharus minimus* | 1.6 | <https://ftp.ncbi.nlm.nih.gov/genomes/all/GCA/021/437/185/GCA_021437185.1_HU_Cminimus_1.0/> | SRR15809697 | 0.00949559 | 0.0029070 | 0.000000 | 0.0029070 | 94.37 | 13.40 | 96.20 |
| *Catharus minimus* | 1.6 | https://ftp.ncbi.nlm.nih.gov/genomes/all/GCA/021/437/185/GCA_021437185.1_HU_Cminimus_1.0/ | SRR15809696 | 0.00962253 | 0.0063790 | 0.000000 | 0.0063790 | 94.35 | 13.30 | 94.80 |
| *Catharus minimus* | 1.6 | https://ftp.ncbi.nlm.nih.gov/genomes/all/GCA/021/437/185/GCA_021437185.1_HU_Cminimus_1.0/ | SRR15809691 | 0.00981764 | 0.0001000 | 0.000000 | 0.0001000 | 94.49 | 14.13 | 96.80 |
| *Catharus minimus* | 1.6 | https://ftp.ncbi.nlm.nih.gov/genomes/all/GCA/021/437/185/GCA_021437185.1_HU_Cminimus_1.0/ | SRR15809688 | 0.00966219 | 0.0001000 | 0.000000 | 0.0001000 | 94.48 | 13.88 | 96.70 |
| *Catharus minimus* | 1.6 | https://ftp.ncbi.nlm.nih.gov/genomes/all/GCA/021/437/185/GCA_021437185.1_HU_Cminimus_1.0/ | SRR15809690 | 0.00959340 | 0.0005040 | 0.000000 | 0.0005040 | 94.43 | 15.37 | 97.00 |
| *Catharus minimus* | 1.6 | https://ftp.ncbi.nlm.nih.gov/genomes/all/GCA/021/437/185/GCA_021437185.1_HU_Cminimus_1.0/ | SRR15809686 | 0.00967854 | 0.0036330 | 0.000000 | 0.0036330 | 94.39 | 13.64 | 97.10 |
| *Catharus minimus* | 1.6 | https://ftp.ncbi.nlm.nih.gov/genomes/all/GCA/021/437/185/GCA_021437185.1_HU_Cminimus_1.0/ | SRR15809692 | 0.00986726 | 0.0000000 | 0.000000 | 0.0000000 | 94.51 | 15.49 | 96.90 |
| *Catharus minimus* | 1.6 | https://ftp.ncbi.nlm.nih.gov/genomes/all/GCA/021/437/185/GCA_021437185.1_HU_Cminimus_1.0/ | SRR15809684 | 0.00961547 | 0.0002030 | 0.000000 | 0.0002030 | 94.33 | 12.31 | 96.96 |
| *Catharus minimus* | 1.6 | https://ftp.ncbi.nlm.nih.gov/genomes/all/GCA/021/437/185/GCA_021437185.1_HU_Cminimus_1.0/ | SRR15809685 | 0.00947683 | 0.0011710 | 0.000000 | 0.0011710 | 94.54 | 15.46 | 97.00 |
| *Catharus minimus* | 1.6 | https://ftp.ncbi.nlm.nih.gov/genomes/all/GCA/021/437/185/GCA_021437185.1_HU_Cminimus_1.0/ | SRR15809694 | 0.00952498 | 0.0004200 | 0.000000 | 0.0004200 | 94.50 | 15.91 | 96.70 |
| *Catharus minimus* | 1.6 | https://ftp.ncbi.nlm.nih.gov/genomes/all/GCA/021/437/185/GCA_021437185.1_HU_Cminimus_1.0/ | SRR15809683 | 0.00950447 | 0.0001300 | 0.000000 | 0.0001300 | 94.49 | 14.29 | 97.00 |
| *Catharus minimus* | 1.6 | https://ftp.ncbi.nlm.nih.gov/genomes/all/GCA/021/437/185/GCA_021437185.1_HU_Cminimus_1.0/ | SRR15809687 | 0.00941011 | 0.0013240 | 0.000000 | 0.0013240 | 94.47 | 13.49 | 97.20 |
| *Catharus minimus* | 1.6 | https://ftp.ncbi.nlm.nih.gov/genomes/all/GCA/021/437/185/GCA_021437185.1_HU_Cminimus_1.0/ | SRR15809681 | 0.00903171 | 0.0051280 | 0.000000 | 0.0051280 | 94.14 | 10.10 | 97.13 |
| *Catharus minimus* | 1.6 | https://ftp.ncbi.nlm.nih.gov/genomes/all/GCA/021/437/185/GCA_021437185.1_HU_Cminimus_1.0/ | SRR15809689 | 0.00916410 | 0.0091440 | 0.000000 | 0.0091440 | 94.50 | 17.16 | 97.00 |
| *Catharus minimus* | 1.6 | https://ftp.ncbi.nlm.nih.gov/genomes/all/GCA/021/437/185/GCA_021437185.1_HU_Cminimus_1.0/ | SRR15809695 | 0.00909026 | 0.0000000 | 0.000000 | 0.0000000 | 94.70 | 57.10 | 95.60 |
| *Catharus ustulatus* | 1.6 | <https://ftp.ncbi.nlm.nih.gov/genomes/all/GCF/009/819/885/GCF_009819885.2_bCatUst1.pri.v2/> | SRR15809679 | 0.01085146 | 0.0002373 | 0.000000 | 0.0002373 | 95.57 | 12.73 | 98.30 |
| *Catharus ustulatus* | 1.6 | <https://ftp.ncbi.nlm.nih.gov/genomes/all/GCF/009/819/885/GCF_009819885.2_bCatUst1.pri.v2/> | SRR30550896 | 0.00970136 | 0.0024808 | 0.000000 | 0.0024808 | 32.44 | 1.18 | 99.42 |
| *Catharus ustulatus* | 1.6 | <https://ftp.ncbi.nlm.nih.gov/genomes/all/GCF/009/819/885/GCF_009819885.2_bCatUst1.pri.v2/> | SRR30550894 | 0.01088375 | 0.0001335 | 0.000000 | 0.0001335 | 38.69 | 1.83 | 99.74 |
| *Certhia americana* | 64.4 | <https://ftp.ncbi.nlm.nih.gov/genomes/all/GCA/018/697/195/GCA_018697195.1_ASM1869719v1/> | SRR27724194 | 0.00311872 | 0.0029710 | 0.000000 | 0.0029710 | 94.43 | 30.42 | 97.54 |
| *Certhia americana* | 64.4 | <https://ftp.ncbi.nlm.nih.gov/genomes/all/GCA/018/697/195/GCA_018697195.1_ASM1869719v1/> | SRR27724198 | 0.00420834 | 0.0000000 | 0.000000 | 0.0000000 | 94.43 | 39.06 | 99.50 |
| *Certhia americana* | 64.4 | <https://ftp.ncbi.nlm.nih.gov/genomes/all/GCA/018/697/195/GCA_018697195.1_ASM1869719v1/> | SRR27724196 | 0.00409018 | 0.0000971 | 0.000000 | 0.0000971 | 94.43 | 36.62 | 99.04 |
| *Certhia americana* | 64.4 | <https://ftp.ncbi.nlm.nih.gov/genomes/all/GCA/018/697/195/GCA_018697195.1_ASM1869719v1/> | SRR27724197 | 0.00275448 | 0.0032216 | 0.000000 | 0.0032216 | 94.42 | 33.32 | 98.73 |
| *Certhia americana* | 64.4 | <https://ftp.ncbi.nlm.nih.gov/genomes/all/GCA/018/697/195/GCA_018697195.1_ASM1869719v1/> | SRR27724186 | 0.00264605 | 0.0210995 | 0.000000 | 0.0210995 | 94.41 | 36.73 | 98.88 |
| *Chloebia gouldiae* | 17.1 | <https://ftp.ncbi.nlm.nih.gov/genomes/all/GCA/003/676/055/GCA_003676055.1_GouldianFinch/> | SRR8444791 | 0.00701808 | 0.1102165 | 0.004392 | 0.1146082 | 94.21 | 5.14 | 98.64 |
| *Chloebia gouldiae* | 17.1 | <https://ftp.ncbi.nlm.nih.gov/genomes/all/GCA/003/676/055/GCA_003676055.1_GouldianFinch/> | SRR8444798 | 0.00686496 | 0.1063307 | 0.037279 | 0.1436094 | 94.10 | 5.06 | 98.63 |
| *Chloebia gouldiae* | 17.1 | <https://ftp.ncbi.nlm.nih.gov/genomes/all/GCA/003/676/055/GCA_003676055.1_GouldianFinch/> | SRR8444790 | 0.00681900 | 0.0601514 | 0.005211 | 0.0653624 | 95.32 | 5.69 | 98.84 |
| *Chloebia gouldiae* | 17.1 | <https://ftp.ncbi.nlm.nih.gov/genomes/all/GCA/003/676/055/GCA_003676055.1_GouldianFinch/> | SRR8444793 | 0.00730263 | 0.1031438 | 0.005840 | 0.1089841 | 87.93 | 2.98 | 98.85 |
| *Chloebia gouldiae* | 17.1 | <https://ftp.ncbi.nlm.nih.gov/genomes/all/GCA/003/676/055/GCA_003676055.1_GouldianFinch/> | SRR8444785 | 0.00531785 | 0.1753783 | 0.042243 | 0.2176208 | 93.43 | 5.27 | 98.67 |
| *Chloebia gouldiae* | 17.1 | <https://ftp.ncbi.nlm.nih.gov/genomes/all/GCA/003/676/055/GCA_003676055.1_GouldianFinch/> | SRR8444794 | 0.00621089 | 0.1157102 | 0.037454 | 0.1531643 | 93.13 | 4.83 | 97.43 |
| *Chloebia gouldiae* | 17.1 | <https://ftp.ncbi.nlm.nih.gov/genomes/all/GCA/003/676/055/GCA_003676055.1_GouldianFinch/> | SRR8444787 | 0.00555626 | 0.1975850 | 0.031253 | 0.2288381 | 91.32 | 3.74 | 96.64 |
| *Chloebia gouldiae* | 17.1 | <https://ftp.ncbi.nlm.nih.gov/genomes/all/GCA/003/676/055/GCA_003676055.1_GouldianFinch/> | SRR8444799 | 0.00565849 | 0.1804060 | 0.061942 | 0.2423480 | 94.08 | 5.33 | 97.76 |
| *Chloebia gouldiae* | 17.1 | <https://ftp.ncbi.nlm.nih.gov/genomes/all/GCA/003/676/055/GCA_003676055.1_GouldianFinch/> | SRR8444783 | 0.00629357 | 0.0414117 | 0.005643 | 0.0470542 | 96.27 | 6.97 | 98.12 |
| *Chloebia gouldiae* | 17.1 | <https://ftp.ncbi.nlm.nih.gov/genomes/all/GCA/003/676/055/GCA_003676055.1_GouldianFinch/> | SRR8444784 | 0.00466888 | 0.2757133 | 0.016318 | 0.2920310 | 92.77 | 4.47 | 97.09 |
| *Chloebia gouldiae* | 17.1 | <https://ftp.ncbi.nlm.nih.gov/genomes/all/GCA/003/676/055/GCA_003676055.1_GouldianFinch/> | SRR8444800 | 0.00646335 | 0.0344961 | 0.000000 | 0.0344961 | 95.20 | 5.60 | 98.75 |
| *Chloebia gouldiae* | 17.1 | <https://ftp.ncbi.nlm.nih.gov/genomes/all/GCA/003/676/055/GCA_003676055.1_GouldianFinch/> | SRR8444788 | 0.00675447 | 0.0661564 | 0.012650 | 0.0788067 | 95.23 | 5.63 | 98.87 |
| *Chloebia gouldiae* | 17.1 | <https://ftp.ncbi.nlm.nih.gov/genomes/all/GCA/003/676/055/GCA_003676055.1_GouldianFinch/> | SRR8444802 | 0.00649366 | 0.1061769 | 0.037374 | 0.1435510 | 93.29 | 4.60 | 98.76 |
| *Chloebia gouldiae* | 17.1 | <https://ftp.ncbi.nlm.nih.gov/genomes/all/GCA/003/676/055/GCA_003676055.1_GouldianFinch/> | SRR8444797 | 0.00655660 | 0.1176273 | 0.048516 | 0.1661431 | 92.24 | 4.17 | 98.71 |
| *Chloebia gouldiae* | 17.1 | <https://ftp.ncbi.nlm.nih.gov/genomes/all/GCA/003/676/055/GCA_003676055.1_GouldianFinch/> | SRR8444805 | 0.00692464 | 0.1474488 | 0.019801 | 0.1672497 | 93.34 | 4.64 | 98.73 |
| *Chloebia gouldiae* | 17.1 | <https://ftp.ncbi.nlm.nih.gov/genomes/all/GCA/003/676/055/GCA_003676055.1_GouldianFinch/> | SRR8444792 | 0.00622691 | 0.1566179 | 0.011821 | 0.1684393 | 95.01 | 5.78 | 98.75 |
| *Chloebia gouldiae* | 17.1 | <https://ftp.ncbi.nlm.nih.gov/genomes/all/GCA/003/676/055/GCA_003676055.1_GouldianFinch/> | SRR8444803 | 0.00709189 | 0.0355237 | 0.004346 | 0.0398700 | 94.93 | 5.28 | 98.82 |
| *Chloebia gouldiae* | 17.1 | <https://ftp.ncbi.nlm.nih.gov/genomes/all/GCA/003/676/055/GCA_003676055.1_GouldianFinch/> | SRR8444795 | 0.00620286 | 0.0527145 | 0.003153 | 0.0558678 | 95.06 | 5.07 | 97.38 |
| *Chloebia gouldiae* | 17.1 | <https://ftp.ncbi.nlm.nih.gov/genomes/all/GCA/003/676/055/GCA_003676055.1_GouldianFinch/> | SRR8444806 | 0.00723828 | 0.0867895 | 0.029232 | 0.1160218 | 88.45 | 3.19 | 98.72 |
| *Chloebia gouldiae* | 17.1 | <https://ftp.ncbi.nlm.nih.gov/genomes/all/GCA/003/676/055/GCA_003676055.1_GouldianFinch/> | SRR8444796 | 0.00698859 | 0.0970633 | 0.032225 | 0.1292887 | 93.20 | 4.56 | 98.70 |
| *Chloebia gouldiae* | 17.1 | <https://ftp.ncbi.nlm.nih.gov/genomes/all/GCA/003/676/055/GCA_003676055.1_GouldianFinch/> | SRR8444804 | 0.00524726 | 0.2894210 | 0.100683 | 0.3901041 | 92.96 | 4.51 | 98.58 |
| *Chloebia gouldiae* | 17.1 | <https://ftp.ncbi.nlm.nih.gov/genomes/all/GCA/003/676/055/GCA_003676055.1_GouldianFinch/> | SRR8444789 | 0.00690007 | 0.1448084 | 0.042694 | 0.1875023 | 92.75 | 4.12 | 98.93 |
| *Chloebia gouldiae* | 17.1 | <https://ftp.ncbi.nlm.nih.gov/genomes/all/GCA/003/676/055/GCA_003676055.1_GouldianFinch/> | SRR8444801 | 0.00551283 | 0.2753995 | 0.033622 | 0.3090220 | 93.92 | 4.68 | 98.90 |
| *Chloebia gouldiae* | 17.1 | <https://ftp.ncbi.nlm.nih.gov/genomes/all/GCA/003/676/055/GCA_003676055.1_GouldianFinch/> | SRR8444786 | 0.00795830 | 0.0474574 | 0.005981 | 0.0534381 | 93.37 | 4.34 | 98.80 |
| *Chlorodrepanis viren* | 69.8 | <https://ftp.ncbi.nlm.nih.gov/genomes/all/GCA/003/286/495/GCA_003286495.1_ASM328649v1/> | SRR1718440 | 0.00444763 | 0.0000000 | 0.000000 | 0.0000000 | 95.48 | 26.53 | 99.42 |
| *Cinclus mexicanus* | 69.8 | <https://ftp.ncbi.nlm.nih.gov/genomes/all/GCA/003/286/495/GCA_003286495.1_ASM328649v1/> | SRR10019918 | 0.00146483 | 0.0000000 | 0.000000 | 0.0000000 | 97.90 | 13.84 | 97.12 |
| *Corvus brachyrhynchos* | 7 | <https://ftp.ncbi.nlm.nih.gov/genomes/all/GCF/000/691/975/GCF_000691975.1_ASM69197v1/> | SRR1286115 | 0.00186515 | 0.0556591 | 0.000000 | 0.0556591 | 95.69 | 11.01 | 98.45 |
| *Corvus brachyrhynchos* | 7 | <https://ftp.ncbi.nlm.nih.gov/genomes/all/GCF/000/691/975/GCF_000691975.1_ASM69197v1/> | SRR1286123 | 0.00175972 | 0.0171158 | 0.000000 | 0.0171158 | 91.40 | 3.82 | 98.19 |
| *Corvus brachyrhynchos* | 7 | <https://ftp.ncbi.nlm.nih.gov/genomes/all/GCF/000/691/975/GCF_000691975.1_ASM69197v1/> | SRR1286121 | 0.00253037 | 0.0064470 | 0.002408 | 0.0088553 | 48.64 | 0.76 | 98.32 |
| *Corvus brachyrhynchos* | 7 | <https://ftp.ncbi.nlm.nih.gov/genomes/all/GCF/000/691/975/GCF_000691975.1_ASM69197v1/> | SRR1286130 | 0.00206071 | 0.0756220 | 0.021912 | 0.0975338 | 78.51 | 1.97 | 98.11 |
| *Empidonax alnorum* | 7.7 | <https://ftp.ncbi.nlm.nih.gov/genomes/all/GCA/023/644/505/GCA_023644505.1_EAln_1.0/> | SRR14848283 | 0.01030155 | 0.0000000 | 0.000000 | 0.0000000 | 97.23 | 20.78 | 98.95 |
| *Empidonax alnorum* | 7.7 | https://ftp.ncbi.nlm.nih.gov/genomes/all/GCA/023/644/505/GCA_023644505.1_EAln_1.0/ | SRR14848272 | 0.01012335 | 0.0003589 | 0.000000 | 0.0003589 | 97.15 | 17.47 | 98.80 |
| *Empidonax alnorum* | 7.7 | https://ftp.ncbi.nlm.nih.gov/genomes/all/GCA/023/644/505/GCA_023644505.1_EAln_1.0/ | SRR14848258 | 0.01005614 | 0.0000000 | 0.000000 | 0.0000000 | 97.03 | 12.31 | 98.86 |
| *Empidonax alnorum* | 7.7 | https://ftp.ncbi.nlm.nih.gov/genomes/all/GCA/023/644/505/GCA_023644505.1_EAln_1.0/ | SRR14848240 | 0.01008902 | 0.0007126 | 0.000000 | 0.0007126 | 97.19 | 17.75 | 98.60 |
| *Empidonax alnorum* | 7.7 | https://ftp.ncbi.nlm.nih.gov/genomes/all/GCA/023/644/505/GCA_023644505.1_EAln_1.0/ | SRR14848327 | 0.00973907 | 0.0447570 | 0.000000 | 0.0447570 | 98.46 | 15.07 | 98.70 |
| *Empidonax alnorum* | 7.7 | https://ftp.ncbi.nlm.nih.gov/genomes/all/GCA/023/644/505/GCA_023644505.1_EAln_1.0/ | SRR14848265 | 0.00979975 | 0.0435044 | 0.000000 | 0.0435044 | 98.47 | 15.70 | 98.80 |
| *Empidonax alnorum* | 7.7 | https://ftp.ncbi.nlm.nih.gov/genomes/all/GCA/023/644/505/GCA_023644505.1_EAln_1.0/ | SRR14848262 | 0.01030055 | 0.0012072 | 0.000000 | 0.0012072 | 93.66 | 9.32 | 97.72 |
| *Empidonax alnorum* | 7.7 | https://ftp.ncbi.nlm.nih.gov/genomes/all/GCA/023/644/505/GCA_023644505.1_EAln_1.0/ | SRR14848244 | 0.01022428 | 0.0001030 | 0.000000 | 0.0001030 | 97.11 | 14.28 | 98.98 |
| *Empidonax alnorum* | 7.7 | https://ftp.ncbi.nlm.nih.gov/genomes/all/GCA/023/644/505/GCA_023644505.1_EAln_1.0/ | SRR14848339 | 0.00965056 | 0.0438727 | 0.000000 | 0.0438727 | 98.56 | 20.12 | 98.87 |
| *Empidonax alnorum* | 7.7 | https://ftp.ncbi.nlm.nih.gov/genomes/all/GCA/023/644/505/GCA_023644505.1_EAln_1.0/ | SRR14848242 | 0.01010417 | 0.0007933 | 0.000000 | 0.0007933 | 97.15 | 16.23 | 98.93 |
| *Empidonax alnorum* | 7.7 | https://ftp.ncbi.nlm.nih.gov/genomes/all/GCA/023/644/505/GCA_023644505.1_EAln_1.0/ | SRR14848260 | 0.01012887 | 0.0001675 | 0.000000 | 0.0001675 | 97.16 | 16.87 | 98.87 |
| *Empidonax alnorum* | 7.7 | https://ftp.ncbi.nlm.nih.gov/genomes/all/GCA/023/644/505/GCA_023644505.1_EAln_1.0/ | SRR14848261 | 0.01028303 | 0.0003109 | 0.000000 | 0.0003109 | 97.14 | 16.05 | 98.23 |
| *Empidonax alnorum* | 7.7 | https://ftp.ncbi.nlm.nih.gov/genomes/all/GCA/023/644/505/GCA_023644505.1_EAln_1.0/ | SRR14848259 | 0.01022204 | 0.0000000 | 0.000000 | 0.0000000 | 97.19 | 19.45 | 98.93 |
| *Empidonax alnorum* | 7.7 | https://ftp.ncbi.nlm.nih.gov/genomes/all/GCA/023/644/505/GCA_023644505.1_EAln_1.0/ | SRR14848246 | 0.01011676 | 0.0006641 | 0.000000 | 0.0006641 | 97.21 | 20.30 | 98.97 |
| *Empidonax alnorum* | 7.7 | https://ftp.ncbi.nlm.nih.gov/genomes/all/GCA/023/644/505/GCA_023644505.1_EAln_1.0/ | SRR14848245 | 0.01021940 | 0.0000000 | 0.000000 | 0.0000000 | 97.19 | 21.02 | 99.06 |
| *Empidonax alnorum* | 7.7 | https://ftp.ncbi.nlm.nih.gov/genomes/all/GCA/023/644/505/GCA_023644505.1_EAln_1.0/ | SRR14848340 | 0.01018211 | 0.0000996 | 0.000000 | 0.0000996 | 97.27 | 25.13 | 99.00 |
| *Empidonax alnorum* | 7.7 | https://ftp.ncbi.nlm.nih.gov/genomes/all/GCA/023/644/505/GCA_023644505.1_EAln_1.0/ | SRR14848251 | 0.01018580 | 0.0000000 | 0.000000 | 0.0000000 | 97.22 | 20.52 | 99.01 |
| *Empidonax alnorum* | 7.7 | https://ftp.ncbi.nlm.nih.gov/genomes/all/GCA/023/644/505/GCA_023644505.1_EAln_1.0/ | SRR14848243 | 0.01034293 | 0.0002568 | 0.000000 | 0.0002568 | 97.18 | 17.99 | 99.01 |
| *Empidonax alnorum* | 7.7 | https://ftp.ncbi.nlm.nih.gov/genomes/all/GCA/023/644/505/GCA_023644505.1_EAln_1.0/ | SRR14848247 | 0.01008769 | 0.0018286 | 0.000000 | 0.0018286 | 97.06 | 14.70 | 99.14 |
| *Empidonax traillii* | 0.895 | <https://ftp.ncbi.nlm.nih.gov/genomes/all/GCF/003/031/625/GCF_003031625.1_ASM303162v1/> | SRR24292063 | 0.00551319 | 0.0001240 | 0.000000 | 0.0001240 | 87.30 | 3.68 | 98.62 |
| *Empidonax traillii* | 0.895 | <https://ftp.ncbi.nlm.nih.gov/genomes/all/GCF/003/031/625/GCF_003031625.1_ASM303162v1/> | SRR24292062 | 0.00448891 | 0.0160690 | 0.000000 | 0.0160690 | 86.79 | 3.78 | 99.35 |
| *Empidonax traillii* | 0.895 | <https://ftp.ncbi.nlm.nih.gov/genomes/all/GCF/003/031/625/GCF_003031625.1_ASM303162v1/> | SRR24292093 | 0.00421916 | 0.0099230 | 0.000000 | 0.0099230 | 83.74 | 3.51 | 99.27 |
| *Empidonax traillii* | 0.895 | <https://ftp.ncbi.nlm.nih.gov/genomes/all/GCF/003/031/625/GCF_003031625.1_ASM303162v1/> | SRR24291998 | 0.00413762 | 0.0130140 | 0.000000 | 0.0130140 | 80.76 | 3.22 | 98.62 |
| *Empidonax traillii* | 0.895 | <https://ftp.ncbi.nlm.nih.gov/genomes/all/GCF/003/031/625/GCF_003031625.1_ASM303162v1/> | SRR24291955 | 0.00457786 | 0.0318040 | 0.001527 | 0.0333314 | 85.94 | 3.30 | 98.96 |
| *Empidonax traillii* | 0.895 | <https://ftp.ncbi.nlm.nih.gov/genomes/all/GCF/003/031/625/GCF_003031625.1_ASM303162v1/> | SRR24291944 | 0.00480659 | 0.0511920 | 0.000000 | 0.0511920 | 88.73 | 4.17 | 99.07 |
| *Empidonax traillii* | 0.895 | <https://ftp.ncbi.nlm.nih.gov/genomes/all/GCF/003/031/625/GCF_003031625.1_ASM303162v1/> | SRR24291933 | 0.00456688 | 0.0131580 | 0.000000 | 0.0131580 | 87.73 | 3.96 | 99.25 |
| *Empidonax traillii* | 0.895 | <https://ftp.ncbi.nlm.nih.gov/genomes/all/GCF/003/031/625/GCF_003031625.1_ASM303162v1/> | SRR24291890 | 0.00512231 | 0.0024480 | 0.000000 | 0.0024480 | 86.07 | 3.58 | 99.19 |
| *Empidonax traillii* | 0.895 | <https://ftp.ncbi.nlm.nih.gov/genomes/all/GCF/003/031/625/GCF_003031625.1_ASM303162v1/> | SRR24291879 | 0.00505105 | 0.0043960 | 0.000000 | 0.0043960 | 88.23 | 4.98 | 99.21 |
| *Empidonax traillii* | 0.895 | <https://ftp.ncbi.nlm.nih.gov/genomes/all/GCF/003/031/625/GCF_003031625.1_ASM303162v1/> | SRR24291868 | 0.00484354 | 0.0030180 | 0.000000 | 0.0030180 | 87.63 | 3.98 | 99.22 |
| *Empidonax traillii* | 0.895 | <https://ftp.ncbi.nlm.nih.gov/genomes/all/GCF/003/031/625/GCF_003031625.1_ASM303162v1/> | SRR24292061 | 0.00480121 | 0.0335650 | 0.000000 | 0.0335650 | 88.97 | 4.18 | 99.23 |
| *Empidonax traillii* | 0.895 | <https://ftp.ncbi.nlm.nih.gov/genomes/all/GCF/003/031/625/GCF_003031625.1_ASM303162v1/> | SRR24292050 | 0.00528951 | 0.0001910 | 0.000000 | 0.0001910 | 89.32 | 4.64 | 99.15 |
| *Empidonax traillii* | 0.895 | <https://ftp.ncbi.nlm.nih.gov/genomes/all/GCF/003/031/625/GCF_003031625.1_ASM303162v1/> | SRR24292039 | 0.00527403 | 0.0586890 | 0.006270 | 0.0649590 | 87.04 | 3.59 | 99.15 |
| *Empidonax traillii* | 0.895 | <https://ftp.ncbi.nlm.nih.gov/genomes/all/GCF/003/031/625/GCF_003031625.1_ASM303162v1/> | SRR24292028 | 0.00509808 | 0.0131250 | 0.000000 | 0.0131250 | 86.96 | 3.73 | 99.24 |
| *Empidonax traillii* | 0.895 | <https://ftp.ncbi.nlm.nih.gov/genomes/all/GCF/003/031/625/GCF_003031625.1_ASM303162v1/> | SRR24291985 | 0.00373895 | 0.1926430 | 0.000000 | 0.1926430 | 87.58 | 4.10 | 99.21 |
| *Empidonax traillii* | 0.895 | <https://ftp.ncbi.nlm.nih.gov/genomes/all/GCF/003/031/625/GCF_003031625.1_ASM303162v1/> | SRR24291974 | 0.00450181 | 0.0116740 | 0.000000 | 0.0116740 | 86.77 | 3.84 | 99.31 |
| *Empidonax traillii* | 0.895 | <https://ftp.ncbi.nlm.nih.gov/genomes/all/GCF/003/031/625/GCF_003031625.1_ASM303162v1/> | SRR24291931 | 0.00536245 | 0.0515280 | 0.000985 | 0.0525135 | 88.54 | 3.75 | 98.77 |
| *Empidonax traillii* | 0.895 | <https://ftp.ncbi.nlm.nih.gov/genomes/all/GCF/003/031/625/GCF_003031625.1_ASM303162v1/> | SRR24291920 | 0.00517208 | 0.0513680 | 0.001166 | 0.0525344 | 86.80 | 3.55 | 99.18 |
| *Empidonax traillii* | 0.895 | <https://ftp.ncbi.nlm.nih.gov/genomes/all/GCF/003/031/625/GCF_003031625.1_ASM303162v1/> | SRR24291909 | 0.00658500 | 0.0000000 | 0.000000 | 0.0000000 | 88.45 | 3.93 | 99.24 |
| *Empidonax traillii* | 0.895 | <https://ftp.ncbi.nlm.nih.gov/genomes/all/GCF/003/031/625/GCF_003031625.1_ASM303162v1/> | SRR24292104 | 0.00448122 | 0.0207160 | 0.000000 | 0.0207160 | 87.71 | 4.15 | 99.29 |
| *Empidonax traillii* | 0.895 | <https://ftp.ncbi.nlm.nih.gov/genomes/all/GCF/003/031/625/GCF_003031625.1_ASM303162v1/> | SRR24292092 | 0.00483473 | 0.0095290 | 0.000000 | 0.0095290 | 89.79 | 4.78 | 99.28 |
| *Empidonax traillii* | 0.895 | <https://ftp.ncbi.nlm.nih.gov/genomes/all/GCF/003/031/625/GCF_003031625.1_ASM303162v1/> | SRR24292081 | 0.00481906 | 0.0074430 | 0.000000 | 0.0074430 | 88.25 | 4.03 | 99.19 |
| *Empidonax traillii* | 0.895 | <https://ftp.ncbi.nlm.nih.gov/genomes/all/GCF/003/031/625/GCF_003031625.1_ASM303162v1/> | SRR24292024 | 0.00463528 | 0.0714620 | 0.000000 | 0.0714620 | 89.36 | 4.34 | 99.20 |
| *Empidonax traillii* | 0.895 | <https://ftp.ncbi.nlm.nih.gov/genomes/all/GCF/003/031/625/GCF_003031625.1_ASM303162v1/> | SRR24292013 | 0.00557493 | 0.0000000 | 0.000000 | 0.0000000 | 67.29 | 3.32 | 96.76 |
| *Empidonax traillii* | 0.895 | <https://ftp.ncbi.nlm.nih.gov/genomes/all/GCF/003/031/625/GCF_003031625.1_ASM303162v1/> | SRR24292004 | 0.00480204 | 0.0015630 | 0.000000 | 0.0015630 | 72.18 | 3.77 | 97.61 |
| *Empidonax traillii* | 0.895 | <https://ftp.ncbi.nlm.nih.gov/genomes/all/GCF/003/031/625/GCF_003031625.1_ASM303162v1/> | SRR24292003 | 0.00481974 | 0.0005570 | 0.000000 | 0.0005570 | 85.42 | 3.20 | 99.04 |
| *Empidonax traillii* | 0.895 | <https://ftp.ncbi.nlm.nih.gov/genomes/all/GCF/003/031/625/GCF_003031625.1_ASM303162v1/> | SRR24292002 | 0.00451934 | 0.0425460 | 0.000000 | 0.0425460 | 87.27 | 3.64 | 99.20 |
| *Empidonax traillii* | 0.895 | <https://ftp.ncbi.nlm.nih.gov/genomes/all/GCF/003/031/625/GCF_003031625.1_ASM303162v1/> | SRR24292001 | 0.00493941 | 0.0000934 | 0.000000 | 0.0000934 | 81.28 | 3.89 | 98.16 |
| *Empidonax traillii* | 0.895 | <https://ftp.ncbi.nlm.nih.gov/genomes/all/GCF/003/031/625/GCF_003031625.1_ASM303162v1/> | SRR24292000 | 0.00573627 | 0.0007561 | 0.000000 | 0.0007561 | 86.55 | 3.72 | 98.97 |
| *Empidonax traillii* | 0.895 | <https://ftp.ncbi.nlm.nih.gov/genomes/all/GCF/003/031/625/GCF_003031625.1_ASM303162v1/> | SRR24291999 | 0.00472994 | 0.0083348 | 0.000000 | 0.0083348 | 87.55 | 3.84 | 99.14 |
| *Formicarius rufipectus* | 0.836 | <https://ftp.ncbi.nlm.nih.gov/genomes/all/GCA/013/399/695/GCA_013399695.1_ASM1339969v1/> | SRR10019955 | 0.00161092 | 0.0000000 | 0.000000 | 0.0000000 | 98.86 | 9.31 | 95.14 |
| *Geothlypis trichas* | 72.5 | <https://ftp.ncbi.nlm.nih.gov/genomes/all/GCA/009/764/595/GCA_009764595.1_bGeoTri1.pri/> | SRR14793411 | 0.02311047 | 0.0000000 | 0.000000 | 0.0000000 | 97.50 | 43.75 | 97.34 |
| *Geothlypis trichas* | 72.5 | <https://ftp.ncbi.nlm.nih.gov/genomes/all/GCA/009/764/595/GCA_009764595.1_bGeoTri1.pri/> | SRR14793414 | 0.02253544 | 0.0000000 | 0.000000 | 0.0000000 | 97.50 | 43.30 | 97.40 |
| *Geothlypis trichas* | 72.5 | <https://ftp.ncbi.nlm.nih.gov/genomes/all/GCA/009/764/595/GCA_009764595.1_bGeoTri1.pri/> | SRR14793413 | 0.02252061 | 0.0000000 | 0.000000 | 0.0000000 | 97.49 | 42.45 | 97.47 |
| *Geothlypis trichas* | 72.5 | <https://ftp.ncbi.nlm.nih.gov/genomes/all/GCA/009/764/595/GCA_009764595.1_bGeoTri1.pri/> | SRR14793407 | 0.02308994 | 0.0000000 | 0.000000 | 0.0000000 | 97.52 | 50.83 | 97.38 |
| *Geothlypis trichas* | 72.5 | <https://ftp.ncbi.nlm.nih.gov/genomes/all/GCA/009/764/595/GCA_009764595.1_bGeoTri1.pri/> | SRR14793408 | 0.02270453 | 0.0000000 | 0.000000 | 0.0000000 | 97.48 | 41.24 | 97.40 |
| *Geothlypis trichas* | 72.5 | <https://ftp.ncbi.nlm.nih.gov/genomes/all/GCA/009/764/595/GCA_009764595.1_bGeoTri1.pri/> | SRR14793415 | 0.02250705 | 0.0000000 | 0.000000 | 0.0000000 | 97.51 | 44.87 | 97.56 |
| *Geothlypis trichas* | 72.5 | <https://ftp.ncbi.nlm.nih.gov/genomes/all/GCA/009/764/595/GCA_009764595.1_bGeoTri1.pri/> | SRR14793410 | 0.02265173 | 0.0000000 | 0.000000 | 0.0000000 | 97.79 | 46.21 | 97.46 |
| *Geothlypis trichas* | 72.5 | <https://ftp.ncbi.nlm.nih.gov/genomes/all/GCA/009/764/595/GCA_009764595.1_bGeoTri1.pri/> | SRR14793412 | 0.02275100 | 0.0000000 | 0.000000 | 0.0000000 | 97.50 | 43.19 | 97.38 |
| *Haemorhous mexicanus* | 77.2 | <https://ftp.ncbi.nlm.nih.gov/genomes/all/GCF/027/477/595/GCF_027477595.1_bHaeMex1.pri/> | SRR28065845 | 0.00777765 | 0.0030917 | 0.000000 | 0.0030917 | 97.51 | 19.01 | 99.35 |
| *Haemorhous mexicanus* | 77.2 | <https://ftp.ncbi.nlm.nih.gov/genomes/all/GCF/027/477/595/GCF_027477595.1_bHaeMex1.pri/> | SRR28065847 | 0.00710268 | 0.0518089 | 0.003156 | 0.0549646 | 96.34 | 18.40 | 99.29 |
| *Haemorhous mexicanus* | 77.2 | <https://ftp.ncbi.nlm.nih.gov/genomes/all/GCF/027/477/595/GCF_027477595.1_bHaeMex1.pri/> | SRR28065849 | 0.00770755 | 0.0016246 | 0.000000 | 0.0016246 | 97.53 | 20.13 | 99.42 |
| *Haemorhous mexicanus* | 77.2 | <https://ftp.ncbi.nlm.nih.gov/genomes/all/GCF/027/477/595/GCF_027477595.1_bHaeMex1.pri/> | SRR28065839 | 0.00744329 | 0.0353837 | 0.001983 | 0.0373668 | 98.23 | 24.45 | 99.34 |
| *Haemorhous mexicanus* | 77.2 | <https://ftp.ncbi.nlm.nih.gov/genomes/all/GCF/027/477/595/GCF_027477595.1_bHaeMex1.pri/> | SRR28065844 | 0.00718918 | 0.0546397 | 0.000000 | 0.0546397 | 98.08 | 20.46 | 99.33 |
| *Haemorhous mexicanus* | 77.2 | <https://ftp.ncbi.nlm.nih.gov/genomes/all/GCF/027/477/595/GCF_027477595.1_bHaeMex1.pri/> | SRR28065843 | 0.00749072 | 0.0231574 | 0.000000 | 0.0231574 | 98.15 | 22.53 | 99.35 |
| *Haemorhous mexicanus* | 77.2 | <https://ftp.ncbi.nlm.nih.gov/genomes/all/GCF/027/477/595/GCF_027477595.1_bHaeMex1.pri/> | SRR28065838 | 0.00781272 | 0.0004338 | 0.000000 | 0.0004338 | 97.72 | 30.00 | 99.41 |
| *Haemorhous mexicanus* | 77.2 | <https://ftp.ncbi.nlm.nih.gov/genomes/all/GCF/027/477/595/GCF_027477595.1_bHaeMex1.pri/> | SRR28065796 | 0.00764565 | 0.0020450 | 0.000000 | 0.0020450 | 97.34 | 18.54 | 99.44 |
| *Haemorhous mexicanus* | 77.2 | <https://ftp.ncbi.nlm.nih.gov/genomes/all/GCF/027/477/595/GCF_027477595.1_bHaeMex1.pri/> | SRR28065846 | 0.00811955 | 0.0020629 | 0.000000 | 0.0020629 | 97.73 | 27.53 | 99.45 |
| *Haemorhous mexicanus* | 77.2 | <https://ftp.ncbi.nlm.nih.gov/genomes/all/GCF/027/477/595/GCF_027477595.1_bHaeMex1.pri/> | SRR28065797 | 0.00778866 | 0.0020335 | 0.000000 | 0.0020335 | 97.72 | 27.20 | 99.40 |
| *Hirundo rustica* | 76.2 | <https://ftp.ncbi.nlm.nih.gov/genomes/all/GCF/015/227/805/GCF_015227805.2_bHirRus1.pri.v3/> | SRR9947063 | 0.00721078 | 0.0000000 | 0.000000 | 0.0000000 | 96.61 | 15.89 | 95.29 |
| *Hirundo rustica* | 76.2 | https://ftp.ncbi.nlm.nih.gov/genomes/all/GCF/015/227/805/GCF_015227805.2_bHirRus1.pri.v3/ | SRR9947064 | 0.00712890 | 0.0000000 | 0.000000 | 0.0000000 | 96.56 | 15.44 | 95.34 |
| *Hirundo rustica* | 76.2 | https://ftp.ncbi.nlm.nih.gov/genomes/all/GCF/015/227/805/GCF_015227805.2_bHirRus1.pri.v3/ | SRR9946690 | 0.00644909 | 0.0007475 | 0.000000 | 0.0007475 | 96.19 | 11.54 | 97.24 |
| *Hirundo rustica* | 76.2 | https://ftp.ncbi.nlm.nih.gov/genomes/all/GCF/015/227/805/GCF_015227805.2_bHirRus1.pri.v3/ | SRR9946691 | 0.00645207 | 0.0000000 | 0.000000 | 0.0000000 | 96.19 | 11.48 | 97.24 |
| *Junco hyemalis oreganus* | 71.3 | <https://ftp.ncbi.nlm.nih.gov/genomes/all/GCA/003/829/775/GCA_003829775.2_dark-eyed_junco_22Sep2020_assembly/> | SRR28065821 | 0.00605801 | 0.0020110 | 0.000000 | 0.0020110 | 96.45 | 19.68 | 97.00 |
| *Junco hyemalis oreganus* | 71.3 | <https://ftp.ncbi.nlm.nih.gov/genomes/all/GCA/003/829/775/GCA_003829775.2_dark-eyed_junco_22Sep2020_assembly/> | SRR28065817 | 0.00608295 | 0.0035180 | 0.000000 | 0.0035180 | 96.42 | 20.06 | 97.10 |
| *Junco hyemalis oreganus* | 71.3 | <https://ftp.ncbi.nlm.nih.gov/genomes/all/GCA/003/829/775/GCA_003829775.2_dark-eyed_junco_22Sep2020_assembly/> | SRR28065814 | 0.00599409 | 0.0090340 | 0.000000 | 0.0090340 | 96.47 | 20.25 | 97.00 |
| *Junco hyemalis oreganus* | 71.3 | <https://ftp.ncbi.nlm.nih.gov/genomes/all/GCA/003/829/775/GCA_003829775.2_dark-eyed_junco_22Sep2020_assembly/> | SRR28065813 | 0.00604990 | 0.0036260 | 0.000000 | 0.0036260 | 96.48 | 19.38 | 97.00 |
| *Junco hyemalis oreganus* | 71.3 | <https://ftp.ncbi.nlm.nih.gov/genomes/all/GCA/003/829/775/GCA_003829775.2_dark-eyed_junco_22Sep2020_assembly/> | SRR28065823 | 0.00608638 | 0.0009530 | 0.000000 | 0.0009530 | 96.46 | 19.57 | 97.20 |
| *Junco hyemalis oreganus* | 71.3 | <https://ftp.ncbi.nlm.nih.gov/genomes/all/GCA/003/829/775/GCA_003829775.2_dark-eyed_junco_22Sep2020_assembly/> | SRR28065816 | 0.00610984 | 0.0025260 | 0.000000 | 0.0025260 | 96.47 | 19.97 | 96.80 |
| *Junco hyemalis oreganus* | 71.3 | <https://ftp.ncbi.nlm.nih.gov/genomes/all/GCA/003/829/775/GCA_003829775.2_dark-eyed_junco_22Sep2020_assembly/> | SRR28065848 | 0.00609514 | 0.0022400 | 0.000000 | 0.0022400 | 96.47 | 19.42 | 97.00 |
| *Junco hyemalis oreganus* | 71.3 | <https://ftp.ncbi.nlm.nih.gov/genomes/all/GCA/003/829/775/GCA_003829775.2_dark-eyed_junco_22Sep2020_assembly/> | SRR28065812 | 0.00611315 | 0.0014460 | 0.000000 | 0.0014460 | 96.46 | 19.46 | 97.10 |
| *Junco hyemalis oreganus* | 71.3 | <https://ftp.ncbi.nlm.nih.gov/genomes/all/GCA/003/829/775/GCA_003829775.2_dark-eyed_junco_22Sep2020_assembly/> | SRR28065818 | 0.00614403 | 0.0012240 | 0.000000 | 0.0012240 | 96.48 | 25.36 | 97.00 |
| *Junco hyemalis oreganus* | 71.3 | <https://ftp.ncbi.nlm.nih.gov/genomes/all/GCA/003/829/775/GCA_003829775.2_dark-eyed_junco_22Sep2020_assembly/> | SRR28065822 | 0.00609976 | 0.0029130 | 0.000000 | 0.0029130 | 96.44 | 19.34 | 96.80 |
| *Junco hyemalis oreganus* | 71.3 | <https://ftp.ncbi.nlm.nih.gov/genomes/all/GCA/003/829/775/GCA_003829775.2_dark-eyed_junco_22Sep2020_assembly/> | SRR28065815 | 0.00614955 | 0.0010070 | 0.000000 | 0.0010070 | 96.47 | 19.91 | 97.10 |
| *Junco hyemalis oreganus* | 71.3 | <https://ftp.ncbi.nlm.nih.gov/genomes/all/GCA/003/829/775/GCA_003829775.2_dark-eyed_junco_22Sep2020_assembly/> | SRR28065819 | 0.01405080 | 0.0000000 | 0.000000 | 0.0000000 | 96.51 | 19.62 | 96.90 |
| *Lanius ludovicianus* | 0.685 | <https://ftp.ncbi.nlm.nih.gov/genomes/all/GCA/013/397/235/GCA_013397235.1_ASM1339723v1/> | SRR10019938 | 0.001124255 | 0.0000000 | 0.000000 | 0.0000000 | 99.05 | 13.31 | 98.15 |
| *Leiothrix lutea* | 0.297 | https://ftp.ncbi.nlm.nih.gov/genomes/all/GCA/013/400/445/GCA_013400445.1_ASM1340044v1/ | SRR9946582 | 0.00998733 | 0.0000000 | 0.000000 | 0.0000000 | 94.43 | 14.59 | 93.07 |
| *Lepidothrix coronata minuscula* | 5 | <https://ftp.ncbi.nlm.nih.gov/genomes/all/GCF/001/604/755/GCF_001604755.1_Lepidothrix_coronata-1.0/> | SRR14455245 | 0.00791536 | 0.0000000 | 0.000000 | 0.0000000 | 79.85 | 3.73 | 96.79 |
| *Leucosticte australis* | 72 | <https://ftp.ncbi.nlm.nih.gov/genomes/all/GCA/025/504/685/GCA_025504685.1_ASM2550468v1/> | SRR12540934 | 0.00269794 | 0.0045086 | 0.000000 | 0.0045086 | 89.44 | 4.56 | 99.56 |
| *Leucosticte australis* | 72 | https://ftp.ncbi.nlm.nih.gov/genomes/all/GCA/025/504/685/GCA_025504685.1_ASM2550468v1/ | SRR12540935 | 0.00282654 | 0.0037854 | 0.000000 | 0.0037854 | 91.88 | 5.37 | 99.63 |
| *Leucosticte australis* | 72 | https://ftp.ncbi.nlm.nih.gov/genomes/all/GCA/025/504/685/GCA_025504685.1_ASM2550468v1/ | SRR12540900 | 0.00302217 | 0.0004359 | 0.000000 | 0.0004359 | 95.42 | 8.84 | 99.65 |
| *Leucosticte australis* | 72 | https://ftp.ncbi.nlm.nih.gov/genomes/all/GCA/025/504/685/GCA_025504685.1_ASM2550468v1/ | SRR12540889 | 0.00299312 | 0.0050549 | 0.000000 | 0.0050549 | 95.79 | 8.31 | 99.49 |
| *Leucosticte australis* | 72 | https://ftp.ncbi.nlm.nih.gov/genomes/all/GCA/025/504/685/GCA_025504685.1_ASM2550468v1/ | SRR12540898 | 0.00315314 | 0.0004433 | 0.000000 | 0.0004433 | 95.64 | 8.63 | 99.57 |
| *Leucosticte australis* | 72 | https://ftp.ncbi.nlm.nih.gov/genomes/all/GCA/025/504/685/GCA_025504685.1_ASM2550468v1/ | SRR12540897 | 0.00294918 | 0.0105980 | 0.000000 | 0.0105980 | 95.76 | 8.64 | 99.50 |
| *Leucosticte australis* | 72 | https://ftp.ncbi.nlm.nih.gov/genomes/all/GCA/025/504/685/GCA_025504685.1_ASM2550468v1/ | SRR12540896 | 0.00306958 | 0.0019192 | 0.000000 | 0.0019192 | 95.84 | 8.95 | 99.23 |
| *Leucosticte australis* | 72 | https://ftp.ncbi.nlm.nih.gov/genomes/all/GCA/025/504/685/GCA_025504685.1_ASM2550468v1/ | SRR12540901 | 0.00279115 | 0.0010343 | 0.000000 | 0.0010343 | 86.91 | 3.88 | 99.46 |
| *Leucosticte australis* | 72 | https://ftp.ncbi.nlm.nih.gov/genomes/all/GCA/025/504/685/GCA_025504685.1_ASM2550468v1/ | SRR12540894 | 0.00319453 | 0.0009625 | 0.000000 | 0.0009625 | 96.89 | 11.26 | 99.59 |
| *Leucosticte australis* | 72 | https://ftp.ncbi.nlm.nih.gov/genomes/all/GCA/025/504/685/GCA_025504685.1_ASM2550468v1/ | SRR12540887 | 0.00304631 | 0.0023474 | 0.000000 | 0.0023474 | 95.55 | 8.40 | 99.61 |
| *Leucosticte australis* | 72 | https://ftp.ncbi.nlm.nih.gov/genomes/all/GCA/025/504/685/GCA_025504685.1_ASM2550468v1/ | SRR12540886 | 0.00285555 | 0.0013436 | 0.000000 | 0.0013436 | 86.87 | 3.84 | 99.52 |
| *Leucosticte australis* | 72 | https://ftp.ncbi.nlm.nih.gov/genomes/all/GCA/025/504/685/GCA_025504685.1_ASM2550468v1/ | SRR12540899 | 0.00331208 | 0.0056896 | 0.000000 | 0.0056896 | 97.06 | 12.09 | 98.90 |
| *Leucosticte australis* | 72 | https://ftp.ncbi.nlm.nih.gov/genomes/all/GCA/025/504/685/GCA_025504685.1_ASM2550468v1/ | SRR12540902 | 0.00331650 | 0.0002162 | 0.000000 | 0.0002162 | 97.19 | 11.90 | 99.14 |
| *Loxia curvirostra* | 3.1 | <https://ftp.ncbi.nlm.nih.gov/genomes/all/GCA/013/398/455/GCA_013398455.1_ASM1339845v1/> | SRR9853720 | 0.00322856 | 0.0000000 | 0.000000 | 0.0000000 | 99.03 | 36.95 | 95.20 |
| *Loxia leucoptera* | 3.1 | <https://ftp.ncbi.nlm.nih.gov/genomes/all/GCA/013/398/455/GCA_013398455.1_ASM1339845v1/> | SRR10019967 | 0.00457018 | 0.0000000 | 0.000000 | 0.0000000 | 96.94 | 10.04 | 99.21 |
| *Melospiza melodia* | 77.5 | <https://ftp.ncbi.nlm.nih.gov/genomes/all/GCF/035/770/615/GCF_035770615.1_bMelMel2.pri/> | SRR26156228 | 0.00492720 | 0.0165908 | 0.000937 | 0.0175282 | 94.33 | 58.39 | 99.54 |
| *Melospiza melodia* | 77.5 | https://ftp.ncbi.nlm.nih.gov/genomes/all/GCF/035/770/615/GCF_035770615.1_bMelMel2.pri/ | SRR26156235 | 0.00415941 | 0.0446222 | 0.000000 | 0.0446222 | 93.43 | 40.39 | 99.53 |
| *Melospiza melodia* | 77.5 | https://ftp.ncbi.nlm.nih.gov/genomes/all/GCF/035/770/615/GCF_035770615.1_bMelMel2.pri/ | SRR26156233 | 0.00495484 | 0.0111709 | 0.000000 | 0.0111709 | 93.85 | 42.27 | 99.57 |
| *Melospiza melodia* | 77.5 | https://ftp.ncbi.nlm.nih.gov/genomes/all/GCF/035/770/615/GCF_035770615.1_bMelMel2.pri/ | SRR26156236 | 0.00466836 | 0.0484534 | 0.000000 | 0.0484534 | 96.60 | 40.40 | 99.58 |
| *Melospiza melodia* | 77.5 | https://ftp.ncbi.nlm.nih.gov/genomes/all/GCF/035/770/615/GCF_035770615.1_bMelMel2.pri/ | SRR26156232 | 0.00476893 | 0.0181530 | 0.000000 | 0.0181530 | 93.71 | 44.03 | 99.50 |
| *Melospiza melodia* | 77.5 | https://ftp.ncbi.nlm.nih.gov/genomes/all/GCF/035/770/615/GCF_035770615.1_bMelMel2.pri/ | SRR26156234 | 0.00475692 | 0.0355121 | 0.001066 | 0.0365777 | 96.95 | 50.82 | 99.54 |
| *Melospiza melodia* | 77.5 | https://ftp.ncbi.nlm.nih.gov/genomes/all/GCF/035/770/615/GCF_035770615.1_bMelMel2.pri/ | SRR26156230 | 0.00481952 | 0.0197081 | 0.000000 | 0.0197081 | 93.96 | 43.27 | 99.57 |
| *Melospiza melodia* | 77.5 | https://ftp.ncbi.nlm.nih.gov/genomes/all/GCF/035/770/615/GCF_035770615.1_bMelMel2.pri/ | SRR26156229 | 0.00456987 | 0.0493038 | 0.002259 | 0.0515626 | 97.07 | 52.64 | 99.55 |
| *Melospiza melodia* | 77.5 | https://ftp.ncbi.nlm.nih.gov/genomes/all/GCF/035/770/615/GCF_035770615.1_bMelMel2.pri/ | SRR26156231 | 0.00478021 | 0.0265357 | 0.000845 | 0.0273803 | 94.38 | 49.04 | 99.52 |
| *Melospiza melodia* | 77.5 | https://ftp.ncbi.nlm.nih.gov/genomes/all/GCF/035/770/615/GCF_035770615.1_bMelMel2.pri/ | SRR26156237 | 0.00470961 | 0.0422155 | 0.000000 | 0.0422155 | 96.21 | 32.89 | 99.60 |
| *Melospiza melodia gouldii* | 77.5 | <https://ftp.ncbi.nlm.nih.gov/genomes/all/GCF/035/770/615/GCF_035770615.1_bMelMel2.pri/> | SRR26115124 | 0.00844545 | 0.0006411 | 0.000000 | 0.0006411 | 59.93 | 1.66 | 97.70 |
| *Melospiza melodia gouldii* | 77.5 | <https://ftp.ncbi.nlm.nih.gov/genomes/all/GCF/035/770/615/GCF_035770615.1_bMelMel2.pri/> | SRR26115100 | 0.00830295 | 0.0028422 | 0.000665 | 0.0035071 | 66.21 | 2.03 | 97.80 |
| *Melospiza melodia gouldii* | 77.5 | <https://ftp.ncbi.nlm.nih.gov/genomes/all/GCF/035/770/615/GCF_035770615.1_bMelMel2.pri/> | SRR26115113 | 0.00805363 | 0.0257288 | 0.019687 | 0.0454159 | 75.18 | 2.44 | 98.50 |
| *Melospiza melodia gouldii* | 77.5 | <https://ftp.ncbi.nlm.nih.gov/genomes/all/GCF/035/770/615/GCF_035770615.1_bMelMel2.pri/> | SRR26115099 | 0.00850656 | 0.0537502 | 0.044372 | 0.0981223 | 61.48 | 1.49 | 98.10 |
| *Melospiza melodia gouldii* | 77.5 | <https://ftp.ncbi.nlm.nih.gov/genomes/all/GCF/035/770/615/GCF_035770615.1_bMelMel2.pri/> | SRR26115136 | 0.00792201 | 0.0157141 | 0.003732 | 0.0194459 | 68.02 | 2.02 | 98.10 |
| *Melospiza melodia gouldii* | 77.5 | <https://ftp.ncbi.nlm.nih.gov/genomes/all/GCF/035/770/615/GCF_035770615.1_bMelMel2.pri/> | SRR26115102 | 0.00824293 | 0.0082442 | 0.001024 | 0.0092686 | 65.22 | 1.78 | 98.30 |
| *Melospiza melodia gouldii* | 77.5 | <https://ftp.ncbi.nlm.nih.gov/genomes/all/GCF/035/770/615/GCF_035770615.1_bMelMel2.pri/> | SRR26115135 | 0.00823629 | 0.0057753 | 0.000814 | 0.0065890 | 69.57 | 2.27 | 98.00 |
| *Melospiza melodia gouldii* | 77.5 | <https://ftp.ncbi.nlm.nih.gov/genomes/all/GCF/035/770/615/GCF_035770615.1_bMelMel2.pri/> | SRR26115103 | 0.00807792 | 0.0020356 | 0.000000 | 0.0020356 | 76.32 | 2.89 | 98.30 |
| *Melospiza melodia gouldii* | 77.5 | <https://ftp.ncbi.nlm.nih.gov/genomes/all/GCF/035/770/615/GCF_035770615.1_bMelMel2.pri/> | SRR26115098 | 0.00801981 | 0.0547686 | 0.045786 | 0.1005550 | 64.96 | 1.79 | 98.40 |
| *Melospiza melodia gouldii* | 77.5 | <https://ftp.ncbi.nlm.nih.gov/genomes/all/GCF/035/770/615/GCF_035770615.1_bMelMel2.pri/> | SRR26115101 | 0.00858771 | 0.0031550 | 0.000000 | 0.0031550 | 66.20 | 1.97 | 98.20 |
| *Melospiza melodia heermanni* | 77.5 | <https://ftp.ncbi.nlm.nih.gov/genomes/all/GCF/035/770/615/GCF_035770615.1_bMelMel2.pri/> | SRR26115129 | 0.00704378 | 0.0001077 | 0.000000 | 0.0001077 | 82.54 | 3.81 | 99.48 |
| *Melospiza melodia heermanni* | 77.5 | <https://ftp.ncbi.nlm.nih.gov/genomes/all/GCF/035/770/615/GCF_035770615.1_bMelMel2.pri/> | SRR26115133 | 0.00802713 | 0.0000000 | 0.000000 | 0.0000000 | 82.52 | 3.77 | 99.47 |
| *Melospiza melodia heermanni* | 77.5 | <https://ftp.ncbi.nlm.nih.gov/genomes/all/GCF/035/770/615/GCF_035770615.1_bMelMel2.pri/> | SRR26115132 | 0.00958891 | 0.0000000 | 0.000000 | 0.0000000 | 82.26 | 3.90 | 99.57 |
| *Melospiza melodia heermanni* | 77.5 | <https://ftp.ncbi.nlm.nih.gov/genomes/all/GCF/035/770/615/GCF_035770615.1_bMelMel2.pri/> | SRR26115127 | 0.00586904 | 0.0007379 | 0.000000 | 0.0007379 | 82.41 | 3.58 | 99.53 |
| *Melospiza melodia heermanni* | 77.5 | <https://ftp.ncbi.nlm.nih.gov/genomes/all/GCF/035/770/615/GCF_035770615.1_bMelMel2.pri/> | SRR26115128 | 0.00841596 | 0.0005542 | 0.000000 | 0.0005542 | 81.73 | 3.54 | 99.53 |
| *Melospiza melodia heermanni* | 77.5 | <https://ftp.ncbi.nlm.nih.gov/genomes/all/GCF/035/770/615/GCF_035770615.1_bMelMel2.pri/> | SRR26115130 | 0.00697749 | 0.0002853 | 0.000000 | 0.0002853 | 83.00 | 3.83 | 99.54 |
| *Melospiza melodia heermanni* | 77.5 | <https://ftp.ncbi.nlm.nih.gov/genomes/all/GCF/035/770/615/GCF_035770615.1_bMelMel2.pri/> | SRR26115131 | 0.00878923 | 0.0000000 | 0.000000 | 0.0000000 | 82.17 | 3.87 | 99.50 |
| *Melospiza melodia heermanni* | 77.5 | <https://ftp.ncbi.nlm.nih.gov/genomes/all/GCF/035/770/615/GCF_035770615.1_bMelMel2.pri/> | SRR26115134 | 0.01410976 | 0.0000000 | 0.000000 | 0.0000000 | 76.30 | 3.27 | 99.54 |
| *Melospiza melodia maxillaris* | 77.5 | <https://ftp.ncbi.nlm.nih.gov/genomes/all/GCF/035/770/615/GCF_035770615.1_bMelMel2.pri/> | SRR26115120 | 0.00594536 | 0.0000653 | 0.000000 | 0.0000653 | 81.80 | 3.94 | 99.53 |
| *Melospiza melodia maxillaris* | 77.5 | <https://ftp.ncbi.nlm.nih.gov/genomes/all/GCF/035/770/615/GCF_035770615.1_bMelMel2.pri/> | SRR26115123 | 0.00678149 | 0.0000000 | 0.000000 | 0.0000000 | 80.36 | 3.70 | 99.58 |
| *Melospiza melodia maxillaris* | 77.5 | <https://ftp.ncbi.nlm.nih.gov/genomes/all/GCF/035/770/615/GCF_035770615.1_bMelMel2.pri/> | SRR26115121 | 0.00493287 | 0.0016816 | 0.000000 | 0.0016816 | 81.36 | 3.51 | 99.58 |
| *Melospiza melodia maxillaris* | 77.5 | <https://ftp.ncbi.nlm.nih.gov/genomes/all/GCF/035/770/615/GCF_035770615.1_bMelMel2.pri/> | SRR26115126 | 0.00835171 | 0.0000000 | 0.000000 | 0.0000000 | 81.00 | 3.79 | 99.55 |
| *Melospiza melodia maxillaris* | 77.5 | <https://ftp.ncbi.nlm.nih.gov/genomes/all/GCF/035/770/615/GCF_035770615.1_bMelMel2.pri/> | SRR26115122 | 0.00827893 | 0.0000000 | 0.000000 | 0.0000000 | 80.65 | 3.49 | 99.52 |
| *Melospiza melodia maxillaris* | 77.5 | <https://ftp.ncbi.nlm.nih.gov/genomes/all/GCF/035/770/615/GCF_035770615.1_bMelMel2.pri/> | SRR26115125 | 0.00569423 | 0.0005059 | 0.000000 | 0.0005059 | 81.04 | 3.56 | 99.50 |
| *Melospiza melodia maxima* | 77.5 | <https://ftp.ncbi.nlm.nih.gov/genomes/all/GCF/035/770/615/GCF_035770615.1_bMelMel2.pri/> | SRR26031820 | 0.00143642 | 0.0814894 | 0.000000 | 0.0814894 | 78.34 | 3.20 | 99.23 |
| *Melospiza melodia maxima* | 77.5 | <https://ftp.ncbi.nlm.nih.gov/genomes/all/GCF/035/770/615/GCF_035770615.1_bMelMel2.pri/> | SRR26031811 | 0.00253444 | 0.0003986 | 0.000000 | 0.0003986 | 80.53 | 4.83 | 99.40 |
| *Melospiza melodia maxima* | 77.5 | <https://ftp.ncbi.nlm.nih.gov/genomes/all/GCF/035/770/615/GCF_035770615.1_bMelMel2.pri/> | SRR26031810 | 0.00252200 | 0.0003154 | 0.000000 | 0.0003154 | 84.05 | 5.93 | 99.25 |
| *Melospiza melodia maxima* | 77.5 | <https://ftp.ncbi.nlm.nih.gov/genomes/all/GCF/035/770/615/GCF_035770615.1_bMelMel2.pri/> | SRR26031815 | 0.00254557 | 0.0007942 | 0.000000 | 0.0007942 | 86.65 | 5.79 | 99.57 |
| *Melospiza melodia maxima* | 77.5 | <https://ftp.ncbi.nlm.nih.gov/genomes/all/GCF/035/770/615/GCF_035770615.1_bMelMel2.pri/> | SRR26031813 | 0.00235744 | 0.0010301 | 0.000000 | 0.0010301 | 80.24 | 4.32 | 99.64 |
| *Melospiza melodia maxima* | 77.5 | <https://ftp.ncbi.nlm.nih.gov/genomes/all/GCF/035/770/615/GCF_035770615.1_bMelMel2.pri/> | SRR26031818 | 0.00256505 | 0.0008323 | 0.000000 | 0.0008323 | 87.01 | 5.47 | 99.74 |
| *Melospiza melodia maxima* | 77.5 | <https://ftp.ncbi.nlm.nih.gov/genomes/all/GCF/035/770/615/GCF_035770615.1_bMelMel2.pri/> | SRR26031809 | 0.00243030 | 0.0008746 | 0.000000 | 0.0008746 | 83.41 | 4.84 | 99.62 |
| *Melospiza melodia maxima* | 77.5 | <https://ftp.ncbi.nlm.nih.gov/genomes/all/GCF/035/770/615/GCF_035770615.1_bMelMel2.pri/> | SRR26031819 | 0.00255629 | 0.0000000 | 0.000000 | 0.0000000 | 83.69 | 5.42 | 99.53 |
| *Melospiza melodia maxima* | 77.5 | <https://ftp.ncbi.nlm.nih.gov/genomes/all/GCF/035/770/615/GCF_035770615.1_bMelMel2.pri/> | SRR26031814 | 0.00236447 | 0.0027454 | 0.001647 | 0.0027454 | 81.55 | 4.36 | 99.48 |
| *Melospiza melodia maxima* | 77.5 | <https://ftp.ncbi.nlm.nih.gov/genomes/all/GCF/035/770/615/GCF_035770615.1_bMelMel2.pri/> | SRR26031821 | 0.00246835 | 0.0017710 | 0.001026 | 0.0017710 | 82.14 | 5.11 | 99.47 |
| *Melospiza melodia maxima* | 77.5 | <https://ftp.ncbi.nlm.nih.gov/genomes/all/GCF/035/770/615/GCF_035770615.1_bMelMel2.pri/> | SRR26031812 | 0.00248278 | 0.0023097 | 0.002073 | 0.0043832 | 82.55 | 5.01 | 99.32 |
| *Melospiza melodia maxima* | 77.5 | <https://ftp.ncbi.nlm.nih.gov/genomes/all/GCF/035/770/615/GCF_035770615.1_bMelMel2.pri/> | SRR26031816 | 0.00262510 | 0.0000000 | 0.000000 | 0.0000000 | 85.70 | 6.07 | 99.49 |
| *Melospiza melodia maxima* | 77.5 | <https://ftp.ncbi.nlm.nih.gov/genomes/all/GCF/035/770/615/GCF_035770615.1_bMelMel2.pri/> | SRR10491484 | 0.00241149 | 0.0002179 | 0.000000 | 0.0002179 | 82.02 | 4.69 | 98.81 |
| *Melospiza melodia merrilli* | 77.5 | <https://ftp.ncbi.nlm.nih.gov/genomes/all/GCF/035/770/615/GCF_035770615.1_bMelMel2.pri/> | SRR26031806 | 0.00744250 | 0.0014588 | 0.000000 | 0.0014588 | 87.25 | 5.74 | 99.00 |
| *Melospiza melodia merrilli* | 77.5 | <https://ftp.ncbi.nlm.nih.gov/genomes/all/GCF/035/770/615/GCF_035770615.1_bMelMel2.pri/> | SRR26031795 | 0.00749125 | 0.0009105 | 0.000000 | 0.0009105 | 81.67 | 4.78 | 98.30 |
| *Melospiza melodia merrilli* | 77.5 | <https://ftp.ncbi.nlm.nih.gov/genomes/all/GCF/035/770/615/GCF_035770615.1_bMelMel2.pri/> | SRR26031794 | 0.00638883 | 0.0003999 | 0.000000 | 0.0003999 | 89.25 | 8.99 | 99.00 |
| *Melospiza melodia merrilli* | 77.5 | <https://ftp.ncbi.nlm.nih.gov/genomes/all/GCF/035/770/615/GCF_035770615.1_bMelMel2.pri/> | SRR26031817 | 0.01236483 | 0.0000000 | 0.000000 | 0.0000000 | 90.44 | 12.04 | 97.80 |
| *Melospiza melodia merrilli* | 77.5 | <https://ftp.ncbi.nlm.nih.gov/genomes/all/GCF/035/770/615/GCF_035770615.1_bMelMel2.pri/> | SRR26031829 | 0.00549986 | 0.0007939 | 0.000000 | 0.0007939 | 90.62 | 13.57 | 99.17 |
| *Melospiza melodia merrilli* | 77.5 | <https://ftp.ncbi.nlm.nih.gov/genomes/all/GCF/035/770/615/GCF_035770615.1_bMelMel2.pri/> | SRR26031792 | 0.00631750 | 0.0015468 | 0.000000 | 0.0015468 | 87.07 | 7.87 | 98.90 |
| *Melospiza melodia merrilli* | 77.5 | <https://ftp.ncbi.nlm.nih.gov/genomes/all/GCF/035/770/615/GCF_035770615.1_bMelMel2.pri/> | SRR26031793 | 0.00872044 | 0.0024855 | 0.000000 | 0.0024855 | 82.47 | 4.53 | 98.60 |
| *Melospiza melodia merrilli* | 77.5 | <https://ftp.ncbi.nlm.nih.gov/genomes/all/GCF/035/770/615/GCF_035770615.1_bMelMel2.pri/> | SRR26031828 | 0.00560403 | 0.0003252 | 0.000000 | 0.0003252 | 87.33 | 6.29 | 99.20 |
| *Melospiza melodia pusillula* | 77.5 | <https://ftp.ncbi.nlm.nih.gov/genomes/all/GCF/035/770/615/GCF_035770615.1_bMelMel2.pri/> | SRR26115111 | 0.00825192 | 0.0773915 | 0.055019 | 0.1324102 | 63.26 | 1.60 | 98.20 |
| *Melospiza melodia pusillula* | 77.5 | <https://ftp.ncbi.nlm.nih.gov/genomes/all/GCF/035/770/615/GCF_035770615.1_bMelMel2.pri/> | SRR26115115 | 0.00802852 | 0.0093621 | 0.000000 | 0.0093621 | 79.12 | 3.46 | 98.50 |
| *Melospiza melodia pusillula* | 77.5 | <https://ftp.ncbi.nlm.nih.gov/genomes/all/GCF/035/770/615/GCF_035770615.1_bMelMel2.pri/> | SRR26115112 | 0.00789512 | 0.0109890 | 0.000682 | 0.0116709 | 75.02 | 2.80 | 98.60 |
| *Melospiza melodia pusillula* | 77.5 | <https://ftp.ncbi.nlm.nih.gov/genomes/all/GCF/035/770/615/GCF_035770615.1_bMelMel2.pri/> | SRR26115118 | 0.00869163 | 0.0190899 | 0.011314 | 0.0304035 | 54.09 | 1.52 | 97.70 |
| *Melospiza melodia pusillula* | 77.5 | <https://ftp.ncbi.nlm.nih.gov/genomes/all/GCF/035/770/615/GCF_035770615.1_bMelMel2.pri/> | SRR26115110 | 0.00780814 | 0.0388235 | 0.003472 | 0.0422960 | 73.89 | 2.73 | 98.50 |
| *Melospiza melodia pusillula* | 77.5 | <https://ftp.ncbi.nlm.nih.gov/genomes/all/GCF/035/770/615/GCF_035770615.1_bMelMel2.pri/> | SRR26115116 | 0.00803491 | 0.0095034 | 0.000000 | 0.0095034 | 68.49 | 2.08 | 98.70 |
| *Melospiza melodia pusillula* | 77.5 | <https://ftp.ncbi.nlm.nih.gov/genomes/all/GCF/035/770/615/GCF_035770615.1_bMelMel2.pri/> | SRR26115119 | 0.00813165 | 0.0646755 | 0.044104 | 0.1087797 | 65.46 | 1.86 | 98.40 |
| *Melospiza melodia pusillula* | 77.5 | <https://ftp.ncbi.nlm.nih.gov/genomes/all/GCF/035/770/615/GCF_035770615.1_bMelMel2.pri/> | SRR26115117 | 0.00865655 | 0.0765412 | 0.013049 | 0.0895903 | 68.17 | 2.09 | 98.20 |
| *Melospiza melodia pusillula* | 77.5 | <https://ftp.ncbi.nlm.nih.gov/genomes/all/GCF/035/770/615/GCF_035770615.1_bMelMel2.pri/> | SRR26115114 | 0.00809583 | 0.0089187 | 0.000000 | 0.0089187 | 66.96 | 1.97 | 98.00 |
| *Melospiza melodia samuelis* | 77.5 | <https://ftp.ncbi.nlm.nih.gov/genomes/all/GCF/035/770/615/GCF_035770615.1_bMelMel2.pri/> | SRR26115107 | 0.00943322 | 0.0002136 | 0.000000 | 0.0002136 | 79.38 | 3.33 | 99.60 |
| *Melospiza melodia samuelis* | 77.5 | <https://ftp.ncbi.nlm.nih.gov/genomes/all/GCF/035/770/615/GCF_035770615.1_bMelMel2.pri/> | SRR26115106 | 0.00577263 | 0.0211924 | 0.000000 | 0.0211924 | 83.08 | 4.01 | 99.60 |
| *Melospiza melodia samuelis* | 77.5 | <https://ftp.ncbi.nlm.nih.gov/genomes/all/GCF/035/770/615/GCF_035770615.1_bMelMel2.pri/> | SRR26115105 | 0.00608156 | 0.0006576 | 0.000000 | 0.0006576 | 84.01 | 4.16 | 99.50 |
| *Melospiza melodia samuelis* | 77.5 | <https://ftp.ncbi.nlm.nih.gov/genomes/all/GCF/035/770/615/GCF_035770615.1_bMelMel2.pri/> | SRR26115108 | 0.00584895 | 0.0033477 | 0.000000 | 0.0033477 | 84.34 | 4.27 | 99.50 |
| *Melospiza melodia samuelis* | 77.5 | <https://ftp.ncbi.nlm.nih.gov/genomes/all/GCF/035/770/615/GCF_035770615.1_bMelMel2.pri/> | SRR26115109 | 0.00779643 | 0.0369016 | 0.001690 | 0.0385914 | 84.02 | 3.56 | 99.60 |
| *Melospiza melodia samuelis* | 77.5 | <https://ftp.ncbi.nlm.nih.gov/genomes/all/GCF/035/770/615/GCF_035770615.1_bMelMel2.pri/> | SRR26115104 | 0.00573036 | 0.0077226 | 0.000000 | 0.0077226 | 81.50 | 3.68 | 99.60 |
| *Melospiza melodia sanaka* | 77.5 | <https://ftp.ncbi.nlm.nih.gov/genomes/all/GCF/035/770/615/GCF_035770615.1_bMelMel2.pri/> | SRR26031824 | 0.00530170 | 0.0306611 | 0.000000 | 0.0306611 | 82.25 | 4.66 | 98.80 |
| *Melospiza melodia sanaka* | 77.5 | <https://ftp.ncbi.nlm.nih.gov/genomes/all/GCF/035/770/615/GCF_035770615.1_bMelMel2.pri/> | SRR26031823 | 0.00318448 | 0.0160755 | 0.000000 | 0.0160755 | 86.77 | 8.28 | 98.90 |
| *Melospiza melodia sanaka* | 77.5 | <https://ftp.ncbi.nlm.nih.gov/genomes/all/GCF/035/770/615/GCF_035770615.1_bMelMel2.pri/> | SRR26031827 | 0.00317119 | 0.0716491 | 0.018015 | 0.0896645 | 86.54 | 6.81 | 99.00 |
| *Melospiza melodia sanaka* | 77.5 | <https://ftp.ncbi.nlm.nih.gov/genomes/all/GCF/035/770/615/GCF_035770615.1_bMelMel2.pri/> | SRR26031791 | 0.00333250 | 0.0186337 | 0.000000 | 0.0186337 | 86.38 | 9.07 | 98.80 |
| *Melospiza melodia sanaka* | 77.5 | <https://ftp.ncbi.nlm.nih.gov/genomes/all/GCF/035/770/615/GCF_035770615.1_bMelMel2.pri/> | SRR26031825 | 0.00309957 | 0.0194988 | 0.000801 | 0.0202993 | 89.40 | 12.82 | 99.10 |
| *Melospiza melodia sanaka* | 77.5 | <https://ftp.ncbi.nlm.nih.gov/genomes/all/GCF/035/770/615/GCF_035770615.1_bMelMel2.pri/> | SRR26031822 | 0.00330634 | 0.0251884 | 0.000795 | 0.0259833 | 86.39 | 7.05 | 99.10 |
| *Melospiza melodia sanaka* | 77.5 | <https://ftp.ncbi.nlm.nih.gov/genomes/all/GCF/035/770/615/GCF_035770615.1_bMelMel2.pri/> | SRR26031790 | 0.00590949 | 0.0189337 | 0.000000 | 0.0189337 | 80.69 | 3.57 | 98.90 |
| *Melospiza melodia sanaka* | 77.5 | <https://ftp.ncbi.nlm.nih.gov/genomes/all/GCF/035/770/615/GCF_035770615.1_bMelMel2.pri/> | SRR26031826 | 0.00359541 | 0.0244197 | 0.000000 | 0.0244197 | 87.84 | 8.12 | 98.90 |
| *Molothrus ater* | 70.1 | <https://ftp.ncbi.nlm.nih.gov/genomes/all/GCF/012/460/135/GCF_012460135.2_BPBGC_Mater_1.1/> | SRR9853699 | 0.00522994 | 0.0000000 | 0.000000 | 0.0000000 | 99.84 | 63.37 | 99.70 |
| *Onychorhynchus coronatus* | 3 | <https://ftp.ncbi.nlm.nih.gov/genomes/all/GCA/013/397/825/GCA_013397825.1_ASM1339782v1/> | SRR9946526 | 0.00188584 | 0.0000000 | 0.000000 | 0.0000000 | 98.66 | 14.70 | 96.04 |
| *Passerculus sandwichensis* | 64.4 | <https://ftp.ncbi.nlm.nih.gov/genomes/all/GCA/031/885/435/GCA_031885435.1_bPasSan2.pri/> | SRR15110334 | 0.00916970 | 0.0004639 | 0.000000 | 0.0004639 | 88.87 | 3.51 | 96.70 |
| *Passerculus sandwichensis* | 64.4 | <https://ftp.ncbi.nlm.nih.gov/genomes/all/GCA/031/885/435/GCA_031885435.1_bPasSan2.pri/> | SRR15110340 | 0.00901730 | 0.0020971 | 0.000000 | 0.0020971 | 86.40 | 2.97 | 98.00 |
| *Passerculus sandwichensis* | 64.4 | <https://ftp.ncbi.nlm.nih.gov/genomes/all/GCA/031/885/435/GCA_031885435.1_bPasSan2.pri/> | SRR15110337 | 0.00908629 | 0.0024130 | 0.000000 | 0.0024130 | 87.60 | 3.22 | 97.10 |
| *Passerculus sandwichensis* | 64.4 | <https://ftp.ncbi.nlm.nih.gov/genomes/all/GCA/031/885/435/GCA_031885435.1_bPasSan2.pri/> | SRR15110339 | 0.00898922 | 0.0046053 | 0.000000 | 0.0046053 | 84.51 | 2.75 | 97.40 |
| *Passerculus sandwichensis* | 64.4 | <https://ftp.ncbi.nlm.nih.gov/genomes/all/GCA/031/885/435/GCA_031885435.1_bPasSan2.pri/> | SRR15110335 | 0.00902211 | 0.0051863 | 0.000000 | 0.0051863 | 87.81 | 3.21 | 98.70 |
| *Passerculus sandwichensis* | 64.4 | <https://ftp.ncbi.nlm.nih.gov/genomes/all/GCA/031/885/435/GCA_031885435.1_bPasSan2.pri/> | SRR15110338 | 0.00891448 | 0.0169466 | 0.004502 | 0.0214487 | 88.11 | 3.34 | 97.40 |
| *Peucedramus taeniatus* | 0.183 | <https://ftp.ncbi.nlm.nih.gov/genomes/all/GCA/013/400/575/GCA_013400575.1_ASM1340057v1/> | SRR9947362 | 0.00528301 | 0.0000000 | 0.000000 | 0.0000000 | 98.85 | 15.24 | 97.30 |
| *Phainopepla nitens* | 0.542 | <https://ftp.ncbi.nlm.nih.gov/genomes/all/GCA/013/400/175/GCA_013400175.1_ASM1340017v1/> | SRR9947246 | 0.00348080 | 0.0000000 | 0.000000 | 0.0000000 | 98.52 | 11.32 | 94.68 |
| *Poecile atricapillus* | 107.9 | <https://ftp.ncbi.nlm.nih.gov/genomes/all/GCF/030/490/865/GCF_030490865.1_bPoeAtr1.hap1/> | SRR9853755 | 0.00616945 | 0.0000000 | 0.000000 | 0.0000000 | 95.93 | 34.47 | 97.70 |
| *Polioptila caerulea* | 0.602 | <https://ftp.ncbi.nlm.nih.gov/genomes/all/GCA/013/397/295/GCA_013397295.1_ASM1339729v1/> | SRR10019935 | 0.00998137 | 0.0000000 | 0.000000 | 0.0000000 | 97.78 | 9.02 | 96.23 |
| *Progne subis subis* | 6.1 | <https://ftp.ncbi.nlm.nih.gov/genomes/all/GCA/022/316/685/GCA_022316685.1_UM_PSub_1/> | SRR16505100 | 0.001798708 | 0.0244025 | 0.000000 | 0.0244025 | 71.71 | 6.92 | 64.90 |
| *Progne subis subis* | 6.1 | <https://ftp.ncbi.nlm.nih.gov/genomes/all/GCA/022/316/685/GCA_022316685.1_UM_PSub_1/> | SRR16505109 | 0.002518381 | 0.0905834 | 0.003397 | 0.0939801 | 85.87 | 2.97 | 95.20 |
| *Progne subis subis* | 6.1 | <https://ftp.ncbi.nlm.nih.gov/genomes/all/GCA/022/316/685/GCA_022316685.1_UM_PSub_1/> | SRR16505162 | 0.002430901 | 0.0733972 | 0.003020 | 0.0764170 | 86.45 | 2.99 | 95.40 |
| *Progne subis subis* | 6.1 | <https://ftp.ncbi.nlm.nih.gov/genomes/all/GCA/022/316/685/GCA_022316685.1_UM_PSub_1/> | SRR1289976 | 0.002334424 | 0.1267303 | 0.002293 | 0.1290228 | 83.59 | 2.90 | 95.50 |
| *Pycnonotus jocosus* | 0.218 | <https://ftp.ncbi.nlm.nih.gov/genomes/all/GCA/013/400/435/GCA_013400435.1_ASM1340043v1/> | SRR9946585 | 0.00701212 | 0.0000000 | 0.000000 | 0.0000000 | 98.56 | 9.46 | 97.05 |
| *Quiscalus mexicanus* | 0.0932 | <https://ftp.ncbi.nlm.nih.gov/genomes/all/GCA/013/399/035/GCA_013399035.1_ASM1339903v1/> | SRR9946430 | 0.00018422 | 0.0000000 | 0.000000 | 0.0000000 | 25.45 | 11.47 | 91.35 |
| *Regulus satrapa* | 0.362 | <https://ftp.ncbi.nlm.nih.gov/genomes/all/GCA/013/396/955/GCA_013396955.1_ASM1339695v1/> | SRR9947111 | 0.00652648 | 0.0000000 | 0.000000 | 0.0000000 | 96.61 | 8.54 | 96.90 |
| *Rhodinocichla rosea* | 2.5 | <https://ftp.ncbi.nlm.nih.gov/genomes/all/GCA/013/396/575/GCA_013396575.1_ASM1339657v1/> | SRR9946575 | 0.00253345 | 0.0000000 | 0.000000 | 0.0000000 | 94.31 | 5.95 | 86.38 |
| *Sapayoa aenigma* | 0.363 | <https://ftp.ncbi.nlm.nih.gov/genomes/all/GCA/013/398/745/GCA_013398745.1_ASM1339874v1/> | SRR9946418 | 0.00200977 | 0.0000000 | 0.000000 | 0.0000000 | 96.66 | 12.90 | 92.40 |
| *Setophaga coronata coronata* | 71.4 | <https://ftp.ncbi.nlm.nih.gov/genomes/all/GCA/001/746/935/GCA_001746935.2_mywa_2.1/> | SRR4027560 | 0.00799636 | 0.0000000 | 0.000000 | 0.0000000 | 93.40 | 21.20 | 80.81 |
| *Setophaga kirtlandii* | 71.1 | <https://ftp.ncbi.nlm.nih.gov/genomes/all/GCA/025/769/955/GCA_025769955.1_ASM2576995v1/> | SRR28115982 | 0.00498669 | 0.0216404 | 0.000000 | 0.0216404 | 97.38 | 25.02 | 99.40 |
| *Setophaga kirtlandii* | 71.1 | <https://ftp.ncbi.nlm.nih.gov/genomes/all/GCA/025/769/955/GCA_025769955.1_ASM2576995v1/> | SRR9947156 | 0.00537218 | 0.0119886 | 0.000000 | 0.0119886 | 97.33 | 21.71 | 99.50 |
| *Setophaga kirtlandii* | 71.1 | <https://ftp.ncbi.nlm.nih.gov/genomes/all/GCA/025/769/955/GCA_025769955.1_ASM2576995v1/> | SRR28115983 | 0.00467912 | 0.0722814 | 0.004990 | 0.0772714 | 96.93 | 12.80 | 98.90 |
| *Sialia sialis* | 32.3 | <https://ftp.ncbi.nlm.nih.gov/genomes/all/GCA/009/812/075/GCA_009812075.1_Ss_LA_1.0/> | SRR9891081 | 0.00589427 | 0.0000000 | 0.000000 | 0.0000000 | 95.34 | 7.61 | 95.90 |
| *Sialia sialis* | 32.3 | <https://ftp.ncbi.nlm.nih.gov/genomes/all/GCA/009/812/075/GCA_009812075.1_Ss_LA_1.0/> | SRR27729428 | 0.00588779 | 0.0000000 | 0.000000 | 0.0000000 | 95.55 | 7.98 | 94.70 |
| *Sialia sialis* | 32.3 | <https://ftp.ncbi.nlm.nih.gov/genomes/all/GCA/009/812/075/GCA_009812075.1_Ss_LA_1.0/> | SRR27729429 | 0.00581342 | 0.0000000 | 0.000000 | 0.0000000 | 95.47 | 7.61 | 94.60 |
| *Sialia sialis* | 32.3 | <https://ftp.ncbi.nlm.nih.gov/genomes/all/GCA/009/812/075/GCA_009812075.1_Ss_LA_1.0/> | SRR27729431 | 0.00589934 | 0.0000000 | 0.000000 | 0.0000000 | 95.15 | 7.57 | 94.30 |
| *Sialia sialis* | 32.3 | <https://ftp.ncbi.nlm.nih.gov/genomes/all/GCA/009/812/075/GCA_009812075.1_Ss_LA_1.0/> | SRR27729430 | 0.00588246 | 0.0000000 | 0.000000 | 0.0000000 | 95.39 | 8.16 | 96.20 |
| *Sialia sialis* | 32.3 | <https://ftp.ncbi.nlm.nih.gov/genomes/all/GCA/009/812/075/GCA_009812075.1_Ss_LA_1.0/> | SRR27729427 | 0.00585378 | 0.0001114 | 0.000000 | 0.0001114 | 94.90 | 6.60 | 95.60 |
| *Sialia sialis* | 32.3 | <https://ftp.ncbi.nlm.nih.gov/genomes/all/GCA/009/812/075/GCA_009812075.1_Ss_LA_1.0/> | SRR27729432 | 0.00551997 | 0.0000000 | 0.000000 | 0.0000000 | 96.49 | 109.83 | 93.80 |
| *Spizella passerina* | 0.013 | <https://ftp.ncbi.nlm.nih.gov/genomes/all/GCA/013/401/375/GCA_013401375.1_ASM1340137v1/> | SRR9946504 | 0.00696816 | 0.0000000 | 0.000000 | 0.0000000 | 88.95 | 14.17 | 88.33 |
| *Sturnus vulgaris* | 3.4 | <https://ftp.ncbi.nlm.nih.gov/genomes/all/GCF/001/447/265/GCF_001447265.1_Sturnus_vulgaris-1.0/> | SRR23619989 | 0.00554524 | 0.0013641 | 0.000000 | 0.0013641 | 97.59 | 26.05 | 97.91 |
| *Sturnus vulgaris* | 3.4 | https://ftp.ncbi.nlm.nih.gov/genomes/all/GCF/001/447/265/GCF_001447265.1_Sturnus_vulgaris-1.0/ | SRR23620010 | 0.00550424 | 0.0014402 | 0.000000 | 0.0014402 | 97.57 | 18.38 | 97.70 |
| *Sturnus vulgaris* | 3.4 | https://ftp.ncbi.nlm.nih.gov/genomes/all/GCF/001/447/265/GCF_001447265.1_Sturnus_vulgaris-1.0/ | SRR23620006 | 0.00554088 | 0.0003397 | 0.000000 | 0.0003397 | 97.58 | 22.69 | 97.85 |
| *Sturnus vulgaris* | 3.4 | https://ftp.ncbi.nlm.nih.gov/genomes/all/GCF/001/447/265/GCF_001447265.1_Sturnus_vulgaris-1.0/ | SRR23620005 | 0.00550786 | 0.0012354 | 0.000000 | 0.0012354 | 97.59 | 23.58 | 97.90 |
| *Sturnus vulgaris* | 3.4 | https://ftp.ncbi.nlm.nih.gov/genomes/all/GCF/001/447/265/GCF_001447265.1_Sturnus_vulgaris-1.0/ | SRR23620009 | 0.00555881 | 0.0005361 | 0.000000 | 0.0005361 | 97.58 | 21.71 | 97.90 |
| *Sturnus vulgaris* | 3.4 | https://ftp.ncbi.nlm.nih.gov/genomes/all/GCF/001/447/265/GCF_001447265.1_Sturnus_vulgaris-1.0/ | SRR23620007 | 0.00555448 | 0.0000000 | 0.000000 | 0.0000000 | 97.58 | 20.65 | 97.71 |
| *Sturnus vulgaris* | 3.4 | https://ftp.ncbi.nlm.nih.gov/genomes/all/GCF/001/447/265/GCF_001447265.1_Sturnus_vulgaris-1.0/ | SRR23620008 | 0.00531140 | 0.0104983 | 0.000000 | 0.0104983 | 97.58 | 21.71 | 97.73 |
| *Sturnus vulgaris* | 3.4 | <https://ftp.ncbi.nlm.nih.gov/genomes/all/GCF/001/447/265/GCF_001447265.1_Sturnus_vulgaris-1.0/> | SRR23619990 | 0.00552057 | 0.0010389 | 0.000000 | 0.0010389 | 97.58 | 24.45 | 97.87 |
| *Taeniopygia guttata* | 71 | <https://ftp.ncbi.nlm.nih.gov/genomes/all/GCF/003/957/565/GCF_003957565.2_bTaeGut1.4.pri/> | SRR27431530 | 0.01204316 | 0.0007158 | 0.000000 | 0.0007158 | 98.72 | 10.73 | 98.50 |
| *Taeniopygia guttata* | 71 | <https://ftp.ncbi.nlm.nih.gov/genomes/all/GCF/003/957/565/GCF_003957565.2_bTaeGut1.4.pri/> | SRR25741330 | 0.01052240 | 0.0004073 | 0.000000 | 0.0004073 | 97.24 | 29.59 | 99.27 |
| *Thryothorus ludovicianus* | 0.495 | <https://ftp.ncbi.nlm.nih.gov/genomes/all/GCA/013/397/245/GCA_013397245.1_ASM1339724v1/> | SRR9946695 | 0.00830476 | 0.0000000 | 0.000000 | 0.0000000 | 98.12 | 15.27 | 89.61 |
| *Toxostoma redivivum* | 0.747 | <https://ftp.ncbi.nlm.nih.gov/genomes/all/GCA/013/397/375/GCA_013397375.1_ASM1339737v1/> | SRR9946642 | 0.00096905 | 0.0000000 | 0.000000 | 0.0000000 | 98.78 | 14.58 | 98.26 |
| *Vireo altiloquus* | 0.668 | <https://ftp.ncbi.nlm.nih.gov/genomes/all/GCA/013/396/875/GCA_013396875.1_ASM1339687v1/> | SRR9946742 | 0.00610731 | 0.0000000 | 0.000000 | 0.0000000 | 98.80 | 16.15 | 93.00 |
| *Zonotrichia albicollis* | 4.9 | <https://ftp.ncbi.nlm.nih.gov/genomes/all/GCF/000/385/455/GCF_000385455.1_Zonotrichia_albicollis-1.0.1/> | SRR2016663 | 0.01165065 | 0.0000000 | 0.000000 | 0.0000000 | 95.57 | 30.00 | 95.70 |
| *Zonotrichia albicollis* | 4.9 | <https://ftp.ncbi.nlm.nih.gov/genomes/all/GCF/000/385/455/GCF_000385455.1_Zonotrichia_albicollis-1.0.1/> | SRR2016662 | 0.00680653 | 0.0000000 | 0.000000 | 0.0000000 | 95.30 | 10.88 | 96.10 |
| *Zonotrichia albicollis* | 4.9 | <https://ftp.ncbi.nlm.nih.gov/genomes/all/GCF/000/385/455/GCF_000385455.1_Zonotrichia_albicollis-1.0.1/> | SRR2012824 | 0.00717540 | 0.0002325 | 0.000000 | 0.0002325 | 95.27 | 10.04 | 96.30 |
| *Zonotrichia leucophrys* | 72 | <https://ftp.ncbi.nlm.nih.gov/genomes/all/GCF/028/769/735/GCF_028769735.1_RI_Zleu_2.0/> | SRR25788565 | 0.00641041 | 0.0000000 | 0.000000 | 0.0000000 | 98.48 | 64.01 | 99.47 |
| *Melozone crissalis* | 22 | <https://ftp.ncbi.nlm.nih.gov/genomes/all/GCF/028/551/555/GCF_028551555.1_PUWL_Pcris_2/> | C_1 | 0.001748182 | 0.111665502 | 0.013098133 | 0.124763634 | 95.5036 | 7.1281 | 99.61 |
| *Melozone crissalis* | 22 | <https://ftp.ncbi.nlm.nih.gov/genomes/all/GCF/028/551/555/GCF_028551555.1_PUWL_Pcris_2/> | C_10 | 0.001680738 | 0.095349832 | 0.012555949 | 0.107905781 | 93.686 | 5.8549 | 99.59 |
| *Melozone crissalis* | 22 | <https://ftp.ncbi.nlm.nih.gov/genomes/all/GCF/028/551/555/GCF_028551555.1_PUWL_Pcris_2/> | C_11 | 0.001801201 | 0.086459423 | 0.009119925 | 0.095579348 | 95.0131 | 6.45092 | 99.63 |
| *Melozone crissalis* | 22 | <https://ftp.ncbi.nlm.nih.gov/genomes/all/GCF/028/551/555/GCF_028551555.1_PUWL_Pcris_2/> | C_12 | 0.001692744 | 0.111188619 | 0.014313034 | 0.125501653 | 94.8426 | 6.13516 | 99.6 |
| *Melozone crissalis* | 22 | <https://ftp.ncbi.nlm.nih.gov/genomes/all/GCF/028/551/555/GCF_028551555.1_PUWL_Pcris_2/> | C_13 | 0.001705709 | 0.126989098 | 0.014476341 | 0.141465439 | 95.081 | 6.91459 | 99.58 |
| *Melozone crissalis* | 22 | <https://ftp.ncbi.nlm.nih.gov/genomes/all/GCF/028/551/555/GCF_028551555.1_PUWL_Pcris_2/> | C_14 | 0.001717276 | 0.111358354 | 0.012632219 | 0.123990573 | 94.3282 | 6.49028 | 99.59 |
| *Melozone crissalis* | 22 | <https://ftp.ncbi.nlm.nih.gov/genomes/all/GCF/028/551/555/GCF_028551555.1_PUWL_Pcris_2/> | C_15 | 0.002554755 | 0.013501011 | 0.005175653 | 0.018676664 | 94.8455 | 6.1691 | 99.55 |
| *Melozone crissalis* | 22 | <https://ftp.ncbi.nlm.nih.gov/genomes/all/GCF/028/551/555/GCF_028551555.1_PUWL_Pcris_2/> | C_16 | 0.002434264 | 0.060892176 | 0.028519522 | 0.089411699 | 95.7315 | 6.04795 | 99.5 |
| *Melozone crissalis* | 22 | <https://ftp.ncbi.nlm.nih.gov/genomes/all/GCF/028/551/555/GCF_028551555.1_PUWL_Pcris_2/> | C_17 | 0.002363713 | 0.021551716 | 0.009249808 | 0.030801524 | 93.4002 | 5.74424 | 99.64 |
| *Melozone crissalis* | 22 | <https://ftp.ncbi.nlm.nih.gov/genomes/all/GCF/028/551/555/GCF_028551555.1_PUWL_Pcris_2/> | C_18 | 0.002449061 | 0.062331943 | 0.027294447 | 0.08962639 | 95.97 | 6.34158 | 99.57 |
| *Melozone crissalis* | 22 | <https://ftp.ncbi.nlm.nih.gov/genomes/all/GCF/028/551/555/GCF_028551555.1_PUWL_Pcris_2/> | C_19 | 0.0024867 | 0.020816322 | 0.007316878 | 0.0281332 | 95.1305 | 6.40935 | 99.58 |
| *Melozone crissalis* | 22 | <https://ftp.ncbi.nlm.nih.gov/genomes/all/GCF/028/551/555/GCF_028551555.1_PUWL_Pcris_2/> | C_2 | 0.001638254 | 0.146388021 | 0.025644238 | 0.172032258 | 93.9591 | 6.50239 | 99.57 |
| *Melozone crissalis* | 22 | <https://ftp.ncbi.nlm.nih.gov/genomes/all/GCF/028/551/555/GCF_028551555.1_PUWL_Pcris_2/> | C_20 | 0.002552411 | 0.015222928 | 0.004642653 | 0.019865581 | 95.137 | 6.58177 | 99.62 |
| *Melozone crissalis* | 22 | <https://ftp.ncbi.nlm.nih.gov/genomes/all/GCF/028/551/555/GCF_028551555.1_PUWL_Pcris_2/> | C_21 | 0.002494078 | 0.068990341 | 0.022391705 | 0.091382046 | 96.4553 | 7.29229 | 99.65 |
| *Melozone crissalis* | 22 | <https://ftp.ncbi.nlm.nih.gov/genomes/all/GCF/028/551/555/GCF_028551555.1_PUWL_Pcris_2/> | C_22 | 0.002481536 | 0.017105485 | 0.00161324 | 0.018718725 | 93.6556 | 6.18388 | 99.64 |
| *Melozone crissalis* | 22 | <https://ftp.ncbi.nlm.nih.gov/genomes/all/GCF/028/551/555/GCF_028551555.1_PUWL_Pcris_2/> | C_23 | 0.002512787 | 0.01483177 | 0.006952807 | 0.021784577 | 95.3791 | 6.80575 | 99.59 |
| *Melozone crissalis* | 22 | <https://ftp.ncbi.nlm.nih.gov/genomes/all/GCF/028/551/555/GCF_028551555.1_PUWL_Pcris_2/> | C_24 | 0.002340241 | 0.067587065 | 0.024418065 | 0.092005131 | 95.518 | 5.88137 | 99.63 |
| *Melozone crissalis* | 22 | <https://ftp.ncbi.nlm.nih.gov/genomes/all/GCF/028/551/555/GCF_028551555.1_PUWL_Pcris_2/> | C_25 | 0.002491065 | 0.014286415 | 0.003498812 | 0.017785227 | 94.3551 | 6.56091 | 99.63 |
| *Melozone crissalis* | 22 | <https://ftp.ncbi.nlm.nih.gov/genomes/all/GCF/028/551/555/GCF_028551555.1_PUWL_Pcris_2/> | C_26 | 0.002371333 | 0.060509058 | 0.024381156 | 0.084890214 | 96.249 | 5.82238 | 99.63 |
| *Melozone crissalis* | 22 | <https://ftp.ncbi.nlm.nih.gov/genomes/all/GCF/028/551/555/GCF_028551555.1_PUWL_Pcris_2/> | C_27 | 0.002525685 | 0.026864664 | 0.005668602 | 0.032533266 | 95.5683 | 7.45151 | 99.63 |
| *Melozone crissalis* | 22 | <https://ftp.ncbi.nlm.nih.gov/genomes/all/GCF/028/551/555/GCF_028551555.1_PUWL_Pcris_2/> | C_28 | 0.002502169 | 0.059588993 | 0.02590171 | 0.085490703 | 97.1538 | 7.18524 | 99.62 |
| *Melozone crissalis* | 22 | <https://ftp.ncbi.nlm.nih.gov/genomes/all/GCF/028/551/555/GCF_028551555.1_PUWL_Pcris_2/> | C_29 | 0.002418341 | 0.07071573 | 0.025911756 | 0.096627486 | 95.879 | 6.26741 | 99.59 |
| *Melozone crissalis* | 22 | <https://ftp.ncbi.nlm.nih.gov/genomes/all/GCF/028/551/555/GCF_028551555.1_PUWL_Pcris_2/> | C_3 | 0.001593591 | 0.108368319 | 0.009504543 | 0.117872862 | 94.285 | 5.5879 | 99.61 |
| *Melozone crissalis* | 22 | <https://ftp.ncbi.nlm.nih.gov/genomes/all/GCF/028/551/555/GCF_028551555.1_PUWL_Pcris_2/> | C_30 | 0.002495758 | 0.011411769 | 0.001941446 | 0.013353216 | 95.2178 | 6.68051 | 99.63 |
| *Melozone crissalis* | 22 | <https://ftp.ncbi.nlm.nih.gov/genomes/all/GCF/028/551/555/GCF_028551555.1_PUWL_Pcris_2/> | C_31 | 0.002415622 | 0.01741139 | 0.000811799 | 0.018223189 | 93.6392 | 5.99382 | 99.65 |
| *Melozone crissalis* | 22 | <https://ftp.ncbi.nlm.nih.gov/genomes/all/GCF/028/551/555/GCF_028551555.1_PUWL_Pcris_2/> | C_32 | 0.002407393 | 0.019137817 | 0.007414204 | 0.026552021 | 94.174 | 5.47749 | 99.65 |
| *Melozone crissalis* | 22 | <https://ftp.ncbi.nlm.nih.gov/genomes/all/GCF/028/551/555/GCF_028551555.1_PUWL_Pcris_2/> | C_33 | 0.00247017 | 0.060266707 | 0.027155654 | 0.087422361 | 97.0297 | 6.76194 | 99.62 |
| *Melozone crissalis* | 22 | <https://ftp.ncbi.nlm.nih.gov/genomes/all/GCF/028/551/555/GCF_028551555.1_PUWL_Pcris_2/> | C_34 | 0.002461792 | 0.00993584 | 0.002968083 | 0.012903923 | 94.4055 | 6.08873 | 99.57 |
| *Melozone crissalis* | 22 | <https://ftp.ncbi.nlm.nih.gov/genomes/all/GCF/028/551/555/GCF_028551555.1_PUWL_Pcris_2/> | C_35 | 0.002461289 | 0.035084912 | 0.012468838 | 0.04755375 | 95.4774 | 6.90502 | 99.6 |
| *Melozone crissalis* | 22 | <https://ftp.ncbi.nlm.nih.gov/genomes/all/GCF/028/551/555/GCF_028551555.1_PUWL_Pcris_2/> | C_36 | 0.002430272 | 0.01814261 | 0.006961544 | 0.025104154 | 93.8573 | 5.99037 | 99.66 |
| *Melozone crissalis* | 22 | <https://ftp.ncbi.nlm.nih.gov/genomes/all/GCF/028/551/555/GCF_028551555.1_PUWL_Pcris_2/> | C_37 | 0.002497657 | 0.062009561 | 0.023211951 | 0.085221512 | 97.0514 | 6.93504 | 99.61 |
| *Melozone crissalis* | 22 | <https://ftp.ncbi.nlm.nih.gov/genomes/all/GCF/028/551/555/GCF_028551555.1_PUWL_Pcris_2/> | C_38 | 0.002031075 | 0.073086493 | 0.019200975 | 0.092287468 | 95.7795 | 6.58573 | 99.56 |
| *Melozone crissalis* | 22 | <https://ftp.ncbi.nlm.nih.gov/genomes/all/GCF/028/551/555/GCF_028551555.1_PUWL_Pcris_2/> | C_39 | 0.001989433 | 0.080603099 | 0.024447362 | 0.105050461 | 95.6049 | 6.2542 | 99.6 |
| *Melozone crissalis* | 22 | <https://ftp.ncbi.nlm.nih.gov/genomes/all/GCF/028/551/555/GCF_028551555.1_PUWL_Pcris_2/> | C_4 | 0.001708978 | 0.09847749 | 0.015326848 | 0.113804337 | 94.6924 | 6.30324 | 99.67 |
| *Melozone crissalis* | 22 | <https://ftp.ncbi.nlm.nih.gov/genomes/all/GCF/028/551/555/GCF_028551555.1_PUWL_Pcris_2/> | C_40 | 0.001938238 | 0.040715141 | 0.011406163 | 0.052121304 | 93.4252 | 5.58359 | 99.55 |
| *Melozone crissalis* | 22 | <https://ftp.ncbi.nlm.nih.gov/genomes/all/GCF/028/551/555/GCF_028551555.1_PUWL_Pcris_2/> | C_41 | 0.002002695 | 0.046848462 | 0.0160391 | 0.062887562 | 93.0194 | 5.55288 | 99.65 |
| *Melozone crissalis* | 22 | <https://ftp.ncbi.nlm.nih.gov/genomes/all/GCF/028/551/555/GCF_028551555.1_PUWL_Pcris_2/> | C_42 | 0.001950914 | 0.032218428 | 0.008880259 | 0.041098687 | 92.5897 | 4.99344 | 99.65 |
| *Melozone crissalis* | 22 | <https://ftp.ncbi.nlm.nih.gov/genomes/all/GCF/028/551/555/GCF_028551555.1_PUWL_Pcris_2/> | C_43 | 0.001983263 | 0.090045798 | 0.029858711 | 0.119904509 | 91.9003 | 5.02359 | 99.49 |
| *Melozone crissalis* | 22 | <https://ftp.ncbi.nlm.nih.gov/genomes/all/GCF/028/551/555/GCF_028551555.1_PUWL_Pcris_2/> | C_44 | 0.001675416 | 0.137731535 | 0.036213325 | 0.17394486 | 94.5598 | 5.91181 | 99.64 |
| *Melozone crissalis* | 22 | <https://ftp.ncbi.nlm.nih.gov/genomes/all/GCF/028/551/555/GCF_028551555.1_PUWL_Pcris_2/> | C_45 | 0.002031197 | 0.055056955 | 0.006701387 | 0.061758342 | 94.1102 | 6.72881 | 99.62 |
| *Melozone crissalis* | 22 | <https://ftp.ncbi.nlm.nih.gov/genomes/all/GCF/028/551/555/GCF_028551555.1_PUWL_Pcris_2/> | C_46 | 0.001815753 | 0.069660957 | 0.018593854 | 0.088254811 | 93.161 | 4.88889 | 99.64 |
| *Melozone crissalis* | 22 | <https://ftp.ncbi.nlm.nih.gov/genomes/all/GCF/028/551/555/GCF_028551555.1_PUWL_Pcris_2/> | C_47 | 0.001805018 | 0.074573136 | 0.021585704 | 0.09615884 | 93.0795 | 5.0979 | 99.64 |
| *Melozone crissalis* | 22 | <https://ftp.ncbi.nlm.nih.gov/genomes/all/GCF/028/551/555/GCF_028551555.1_PUWL_Pcris_2/> | C_48 | 0.001830664 | 0.063593716 | 0.016253344 | 0.07984706 | 93.318 | 5.24967 | 99.61 |
| *Melozone crissalis* | 22 | <https://ftp.ncbi.nlm.nih.gov/genomes/all/GCF/028/551/555/GCF_028551555.1_PUWL_Pcris_2/> | C_49 | 0.001847945 | 0.0703524 | 0.016840455 | 0.087192855 | 94.0793 | 5.61112 | 99.61 |
| *Melozone crissalis* | 22 | <https://ftp.ncbi.nlm.nih.gov/genomes/all/GCF/028/551/555/GCF_028551555.1_PUWL_Pcris_2/> | C_5 | 0.001741851 | 0.098201912 | 0.008159853 | 0.106361765 | 94.8162 | 6.73242 | 99.6 |
| *Melozone crissalis* | 22 | <https://ftp.ncbi.nlm.nih.gov/genomes/all/GCF/028/551/555/GCF_028551555.1_PUWL_Pcris_2/> | C_50 | 0.002105775 | 0.019729738 | 0.006864238 | 0.026593976 | 93.7963 | 5.41383 | 99.63 |
| *Melozone crissalis* | 22 | <https://ftp.ncbi.nlm.nih.gov/genomes/all/GCF/028/551/555/GCF_028551555.1_PUWL_Pcris_2/> | C_51 | 0.002161945 | 0.064843175 | 0.030513722 | 0.095356896 | 97.0156 | 6.47922 | 99.61 |
| *Melozone crissalis* | 22 | <https://ftp.ncbi.nlm.nih.gov/genomes/all/GCF/028/551/555/GCF_028551555.1_PUWL_Pcris_2/> | C_52 | 0.002029223 | 0.063248507 | 0.033368102 | 0.096616609 | 94.575 | 5.27528 | 99.55 |
| *Melozone crissalis* | 22 | <https://ftp.ncbi.nlm.nih.gov/genomes/all/GCF/028/551/555/GCF_028551555.1_PUWL_Pcris_2/> | C_53 | 0.002021121 | 0.015230269 | 0.009452264 | 0.024682533 | 93.7978 | 5.03451 | 99.54 |
| *Melozone crissalis* | 22 | <https://ftp.ncbi.nlm.nih.gov/genomes/all/GCF/028/551/555/GCF_028551555.1_PUWL_Pcris_2/> | C_54 | 0.002025175 | 0.09340769 | 0.030917939 | 0.124325629 | 95.9067 | 5.80233 | 99.58 |
| *Melozone crissalis* | 22 | <https://ftp.ncbi.nlm.nih.gov/genomes/all/GCF/028/551/555/GCF_028551555.1_PUWL_Pcris_2/> | C_55 | 0.002106941 | 0.06087153 | 0.024661322 | 0.085532852 | 95.9112 | 5.82951 | 99.59 |
| *Melozone crissalis* | 22 | <https://ftp.ncbi.nlm.nih.gov/genomes/all/GCF/028/551/555/GCF_028551555.1_PUWL_Pcris_2/> | C_56 | 0.002059632 | 0.020028622 | 0.011204765 | 0.031233387 | 94.0246 | 5.51267 | 99.65 |
| *Melozone crissalis* | 22 | <https://ftp.ncbi.nlm.nih.gov/genomes/all/GCF/028/551/555/GCF_028551555.1_PUWL_Pcris_2/> | C_57 | 0.002337212 | 0.019942021 | 0.007325091 | 0.027267112 | 96.2446 | 8.76483 | 99.6 |
| *Melozone crissalis* | 22 | <https://ftp.ncbi.nlm.nih.gov/genomes/all/GCF/028/551/555/GCF_028551555.1_PUWL_Pcris_2/> | C_58 | 0.002052865 | 0.015342003 | 0.006005157 | 0.02134716 | 94.5786 | 5.57082 | 99.62 |
| *Melozone crissalis* | 22 | <https://ftp.ncbi.nlm.nih.gov/genomes/all/GCF/028/551/555/GCF_028551555.1_PUWL_Pcris_2/> | C_59 | 0.002117033 | 0.02069168 | 0.008145078 | 0.028836758 | 94.5589 | 5.72852 | 99.63 |
| *Melozone crissalis* | 22 | <https://ftp.ncbi.nlm.nih.gov/genomes/all/GCF/028/551/555/GCF_028551555.1_PUWL_Pcris_2/> | C_6 | 0.001616399 | 0.132140745 | 0.011632745 | 0.14377349 | 93.7098 | 6.01009 | 99.65 |
| *Melozone crissalis* | 22 | <https://ftp.ncbi.nlm.nih.gov/genomes/all/GCF/028/551/555/GCF_028551555.1_PUWL_Pcris_2/> | C_60 | 0.002074786 | 0.059476845 | 0.029142322 | 0.088619167 | 95.6659 | 5.58466 | 99.55 |
| *Melozone crissalis* | 22 | <https://ftp.ncbi.nlm.nih.gov/genomes/all/GCF/028/551/555/GCF_028551555.1_PUWL_Pcris_2/> | C_61 | 0.002002249 | 0.060779047 | 0.024958674 | 0.085737721 | 94.956 | 4.95999 | 99.59 |
| *Melozone crissalis* | 22 | <https://ftp.ncbi.nlm.nih.gov/genomes/all/GCF/028/551/555/GCF_028551555.1_PUWL_Pcris_2/> | C_62 | 0.002055672 | 0.070334968 | 0.029395354 | 0.099730322 | 96.3715 | 5.97504 | 99.49 |
| *Melozone crissalis* | 22 | <https://ftp.ncbi.nlm.nih.gov/genomes/all/GCF/028/551/555/GCF_028551555.1_PUWL_Pcris_2/> | C_63 | 0.002056123 | 0.067499383 | 0.031801821 | 0.099301204 | 95.9306 | 5.8753 | 99.18 |
| *Melozone crissalis* | 22 | <https://ftp.ncbi.nlm.nih.gov/genomes/all/GCF/028/551/555/GCF_028551555.1_PUWL_Pcris_2/> | C_64 | 0.002180084 | 0.019473948 | 0.00661354 | 0.026087488 | 94.3938 | 6.32825 | 99.54 |
| *Melozone crissalis* | 22 | <https://ftp.ncbi.nlm.nih.gov/genomes/all/GCF/028/551/555/GCF_028551555.1_PUWL_Pcris_2/> | C_65 | 0.001992975 | 0.065206727 | 0.027777952 | 0.092984679 | 95.5237 | 5.77556 | 99.57 |
| *Melozone crissalis* | 22 | <https://ftp.ncbi.nlm.nih.gov/genomes/all/GCF/028/551/555/GCF_028551555.1_PUWL_Pcris_2/> | C_66 | 0.002019152 | 0.063605909 | 0.016590094 | 0.080196003 | 96.0549 | 5.37583 | 99.31 |
| *Melozone crissalis* | 22 | <https://ftp.ncbi.nlm.nih.gov/genomes/all/GCF/028/551/555/GCF_028551555.1_PUWL_Pcris_2/> | C_67 | 0.002026722 | 0.023182813 | 0.010433128 | 0.033615941 | 94.4695 | 5.90083 | 99.54 |
| *Melozone crissalis* | 22 | <https://ftp.ncbi.nlm.nih.gov/genomes/all/GCF/028/551/555/GCF_028551555.1_PUWL_Pcris_2/> | C_7 | 0.001765871 | 0.104881443 | 0.013918741 | 0.118800185 | 95.113 | 6.81779 | 99.59 |
| *Melozone crissalis* | 22 | <https://ftp.ncbi.nlm.nih.gov/genomes/all/GCF/028/551/555/GCF_028551555.1_PUWL_Pcris_2/> | C_8 | 0.001597839 | 0.139707066 | 0.033449284 | 0.17315635 | 95.0227 | 5.41094 | 99.64 |
| *Melozone crissalis* | 22 | <https://ftp.ncbi.nlm.nih.gov/genomes/all/GCF/028/551/555/GCF_028551555.1_PUWL_Pcris_2/> | C_9 | 0.001708446 | 0.093161864 | 0.01596484 | 0.109126704 | 94.6113 | 5.97657 | 99.6 |
| *Melozone crissalis* | 22 | <https://ftp.ncbi.nlm.nih.gov/genomes/all/GCF/028/551/555/GCF_028551555.1_PUWL_Pcris_2/> | I_1 | 0.00185258 | 0.095745606 | 0.024245881 | 0.119991487 | 94.3557 | 5.57488 | 99.62 |
| *Melozone crissalis* | 22 | <https://ftp.ncbi.nlm.nih.gov/genomes/all/GCF/028/551/555/GCF_028551555.1_PUWL_Pcris_2/> | I_10 | 0.00185509 | 0.107760165 | 0.034990491 | 0.142750656 | 95.7567 | 5.41599 | 99.64 |
| *Melozone crissalis* | 22 | <https://ftp.ncbi.nlm.nih.gov/genomes/all/GCF/028/551/555/GCF_028551555.1_PUWL_Pcris_2/> | I_11 | 0.001741518 | 0.10334951 | 0.026821606 | 0.130171115 | 94.8335 | 5.44268 | 99.67 |
| *Melozone crissalis* | 22 | <https://ftp.ncbi.nlm.nih.gov/genomes/all/GCF/028/551/555/GCF_028551555.1_PUWL_Pcris_2/> | I_12 | 0.001879885 | 0.08945819 | 0.026617222 | 0.116075412 | 95.2628 | 6.05066 | 99.64 |
| *Melozone crissalis* | 22 | <https://ftp.ncbi.nlm.nih.gov/genomes/all/GCF/028/551/555/GCF_028551555.1_PUWL_Pcris_2/> | I_13 | 0.001784773 | 0.191783274 | 0.072463632 | 0.264246906 | 97.231 | 7.50475 | 99.57 |
| *Melozone crissalis* | 22 | <https://ftp.ncbi.nlm.nih.gov/genomes/all/GCF/028/551/555/GCF_028551555.1_PUWL_Pcris_2/> | I_14 | 0.002069989 | 0.074743022 | 0.014559767 | 0.08930279 | 95.3991 | 7.39216 | 99.55 |
| *Melozone crissalis* | 22 | <https://ftp.ncbi.nlm.nih.gov/genomes/all/GCF/028/551/555/GCF_028551555.1_PUWL_Pcris_2/> | I_2 | 0.001860445 | 0.095779767 | 0.035697974 | 0.131477741 | 94.5738 | 6.1155 | 99.65 |
| *Melozone crissalis* | 22 | <https://ftp.ncbi.nlm.nih.gov/genomes/all/GCF/028/551/555/GCF_028551555.1_PUWL_Pcris_2/> | I_3 | 0.001262391 | 0.310428943 | 0.112297969 | 0.422726912 | 94.9241 | 5.92699 | 99.61 |
| *Melozone crissalis* | 22 | <https://ftp.ncbi.nlm.nih.gov/genomes/all/GCF/028/551/555/GCF_028551555.1_PUWL_Pcris_2/> | I_4 | 0.001887536 | 0.137085266 | 0.037661801 | 0.174747067 | 97.4333 | 6.96472 | 99.61 |
| *Melozone crissalis* | 22 | <https://ftp.ncbi.nlm.nih.gov/genomes/all/GCF/028/551/555/GCF_028551555.1_PUWL_Pcris_2/> | I_5 | 0.001753165 | 0.136491467 | 0.041003864 | 0.177495331 | 96.1175 | 5.46432 | 99.57 |
| *Melozone crissalis* | 22 | <https://ftp.ncbi.nlm.nih.gov/genomes/all/GCF/028/551/555/GCF_028551555.1_PUWL_Pcris_2/> | I_6 | 0.001706436 | 0.142871154 | 0.058513431 | 0.201384585 | 94.4656 | 4.84513 | 99.6 |
| *Melozone crissalis* | 22 | <https://ftp.ncbi.nlm.nih.gov/genomes/all/GCF/028/551/555/GCF_028551555.1_PUWL_Pcris_2/> | I_7 | 0.001924404 | 0.104419291 | 0.027739045 | 0.132158336 | 95.8211 | 6.33015 | 99.55 |
| *Melozone crissalis* | 22 | <https://ftp.ncbi.nlm.nih.gov/genomes/all/GCF/028/551/555/GCF_028551555.1_PUWL_Pcris_2/> | I_8 | 0.00164598 | 0.208709937 | 0.130378728 | 0.339088665 | 98.3555 | 5.88086 | 99.59 |
| *Melozone crissalis* | 22 | <https://ftp.ncbi.nlm.nih.gov/genomes/all/GCF/028/551/555/GCF_028551555.1_PUWL_Pcris_2/> | I_9 | 0.001934642 | 0.084576371 | 0.023357983 | 0.107934355 | 95.4638 | 6.13388 | 99.65 |

**Table S5.** Bonferroni corrected p-values obtained from pairwise Wilcoxon sign rank tests for differences in individual heterozygosity between California towhee regions. Bold cells are statistically significant (p-value<0.05).

|  | CCAL | INYO | Non-Threatened | OREG | SCAL |
| --- | --- | --- | --- | --- | --- |
| INYO | **0.0030** | - | - | - | - |
| Non-Threatened | **4.2e-15** | **3.9e-08** | - | - | - |
| OREG | **7.5e-06** | **0.0939** | **2.7e-08** | - | - |
| SCAL | **9.5e-09** | **7.5e-06** | **2.5e-09** | **7.5e-06** | - |
| Threatened | 0.4642 | **0.0012** | **1.2e-15** | **6.3e-05** | 0.7398 |

**Table S6.** Bonferroni corrected p-values obtained from pairwise Wilcoxon sign rank tests for differences in the proportion of Runs of Homozygosity (fROH) for lengths 100kb-1Mb. Bold cells are statistically significant (p-value<0.05).

|  | CCAL | INYO | Non-Threatened | OREG | SCAL |
| --- | --- | --- | --- | --- | --- |
| INYO | **1.8e-05** |  | - | - | - |
| Non-Threatened | **2.0e-12** | **1.2e-08** | - | - | - |
| OREG | **1.4e-05** | 1.00000 | **9.7e-09** | - | - |
| SCAL | **0.07891** | **7.5e-06** | **1.3e-07** | **7.5e-06** | - |
| Threatened | 1.00000 | **0.00015** | **2.5e-10** | **7.1e-05** | 1.00000 |

**Table S7.** Bonferroni corrected p-values obtained from pairwise Wilcoxon sign rank tests for differences in the proportion of Runs of Homozygosity (fROH) for lengths > 1 Mb. Bold cells are statistically significant (p-value<0.05).

|  | CCAL | INYO | Non-Threatened | OREG | SCAL |
| --- | --- | --- | --- | --- | --- |
| INYO | **0.00796** | - | - | - | - |
| Non-Threatened | **< 2e-16** | **2.3e-12** | - | - | - |
| OREG | 1.00000 | **0.00106** | **4.0e-11** | - | - |
| SCAL | **0.27419** | **0.00066** | **1.2e-14** | 1.00000 | - |
| Threatened | **1.6e-05** | **6.1e-07** | **4.4e-09** | **0.02719** | 0.05331 |

**Table S8** Bonferroni corrected p-values obtained from pairwise Wilcoxon sign rank tests for differences in the proportion of Total Runs of Homozygosity (fROH). Bold cells are statistically significant (p-value<0.05).

|  | CCAL | INYO | Non-Threatened | OREG | SCAL |
| --- | --- | --- | --- | --- | --- |
| INYO | **3.9e-05** | - | - | - | - |
| Non-Threatened | **6.5e-13** | **2.0e-08** | - | - | - |
| OREG | **0.00015** | 0.84814 | **2.4e-08** | - | - |
| SCAL | 0.09307 | **1.7e-05** | **4.1e-08** | **8.8e-06** |  |
| Threatened | 0.75974 | **1.4e-05** | **8.7e-10** | **0.00011** | 1.00000 |

**Table S9.** *Fst Outlier results.* California towhee genes identified within outlier 50 kb windows. Note, only genes with known orthologs were used for gene ontology enrichment analysis (in bold).

| Contig | Start | Stop | Gene | Biotype | Description |
| --- | --- | --- | --- | --- | --- |
| NW_026535783.1 | **52900001** | **52933451** | **JMY** | **protein_coding** | **junction mediating and regulatory protein, p53 cofactor** |
| NW_026535783.1 | **52875001** | **52925000** | **JMY** | **protein_coding** | **junction mediating and regulatory protein, p53 cofactor** |
| NW_026535783.1 | **52943633** | **52950000** | **HOMER1** | **protein_coding** | **homer scaffold protein 1** |
| NW_026535784.1 | **17775001** | **17825000** | **APBB2** | **protein_coding** | **amyloid beta precursor protein binding family B member 2** |
| NW_026535787.1 | **15652678** | **15680138** | **MOGAT2** | **protein_coding** | **monoacylglycerol O-acyltransferase 2** |
| NW_026535787.1 | **15698741** | **15700000** | **DGAT2** | **protein_coding** | **diacylglycerol O-acyltransferase 2** |
| NW_026535788.1 | 816024 | 825834 | LOC128933538 | lncRNA | uncharacterized LOC128933538 |
| NW_026535788.1 | 816024 | 825000 | LOC128933538 | lncRNA | uncharacterized LOC128933538 |
| NW_026535791.1 | **45000001** | **45050000** | **AFF3** | **protein_coding** | **ALF transcription elongation factor 3** |
| NW_026535791.1 | **44975001** | **45025000** | **AFF3** | **protein_coding** | **ALF transcription elongation factor 3** |
| NW_026535791.1 | **45025001** | **45075000** | **AFF3** | **protein_coding** | **ALF transcription elongation factor 3** |
| NW_026535792.1 | 21160156 | 21161061 | LOC128935095 | protein_coding | olfactory receptor 14C36-like |
| NW_026535792.1 | 21160156 | 21161061 | LOC128935095 | protein_coding | olfactory receptor 14C36-like |
| NW_026535794.1 | **57600001** | **57629533** | **TNS3** | **protein_coding** | **tensin 3** |
| NW_026535794.1 | **57625001** | **57629533** | **TNS3** | **protein_coding** | **tensin 3** |
| NW_026535794.1 | **57760711** | **57769599** | **HUS1** | **protein_coding** | **HUS1 checkpoint clamp component** |
| NW_026535794.1 | **57785466** | **57800000** | **ICE1** | **protein_coding** | **interactor of little elongation complex ELL subunit 1** |
| NW_026535794.1 | **57800001** | **57825432** | **ICE1** | **protein_coding** | **interactor of little elongation complex ELL subunit 1** |
| NW_026535794.1 | **57785466** | **57825000** | **ICE1** | **protein_coding** | **interactor of little elongation complex ELL subunit 1** |
| NW_026535794.1 | 57825653 | 57850000 | LOC128935334 | lncRNA | uncharacterized LOC128935334 |
| NW_026535794.1 | **57900001** | **57950000** | **NFX1** | **protein_coding** | **nuclear transcription factor, X-box binding 1** |
| NW_026535794.1 | **57925001** | **57974939** | **NFX1** | **protein_coding** | **nuclear transcription factor, X-box binding 1** |
| NW_026535795.1 | 14125001 | 14154738 | LOC128935828 | protein_coding | ubiquinol-cytochrome-c reductase complex assembly factor 1 |
| NW_026535795.1 | **14161638** | **14175000** | **FAM83C** | **protein_coding** | **family with sequence similarity 83 member C** |
| NW_026535797.1 | **76421** | **80929** | **NAA20** | **protein_coding** | **N-alpha-acetyltransferase 20, NatB catalytic subunit** |
| NW_026535797.1 | **83514** | **100000** | **MSH4** | **protein_coding** | **mutS homolog 4** |
| NW_026535799.1 | 2392904 | 2393056 | LOC128937718 | pseudo | 5.8S ribosomal RNA |
| NW_026535799.1 | 4488022 | 4488174 | LOC128937715 | pseudo | 5.8S ribosomal RNA |
| NW_026535799.1 | 4489062 | 4497065 | LOC128937695 | pseudo | 28S ribosomal RNA |
| NW_026535803.1 | 227808 | 233525 | LOC128938372 | lncRNA | uncharacterized LOC128938372 |
| NW_026535803.1 | 233521 | 238036 | LOC128938374 | lncRNA | uncharacterized LOC128938374 |
| NW_026535816.1 | **8975001** | **9025000** | **LGR4** | **protein_coding** | **leucine rich repeat containing G protein-coupled receptor 4** |
| NW_026535816.1 | **9000001** | **9046352** | **LGR4** | **protein_coding** | **leucine rich repeat containing G protein-coupled receptor 4** |
| NW_026535816.1 | **9025001** | **9046352** | **LGR4** | **protein_coding** | **leucine rich repeat containing G protein-coupled receptor 4** |
| NW_026535816.1 | **9052596** | **9072502** | **CCDC34** | **protein_coding** | **coiled-coil domain containing 34** |
| NW_026535824.1 | 7651625 | 7653100 | LOC128943435 | lncRNA | uncharacterized LOC128943435 |
| NW_026535824.1 | 9000001 | 9050000 | LOC128943524 | protein_coding | zinc finger protein 665-like |
| NW_026535824.1 | 9025001 | 9075000 | LOC128943524 | protein_coding | zinc finger protein 665-like |
| NW_026535831.1 | 2454034 | 2470698 | LOC128943673 | protein_coding | synaptonemal complex protein 1-like |
| NW_026535831.1 | 4134217 | 4135693 | LOC128943666 | lncRNA | uncharacterized LOC128943666 |
| NW_026535846.1 | 1738869 | 1739825 | LOC128945871 | pseudo | olfactory receptor 14J1-like |
| NW_026535846.1 | 1766428 | 1767360 | LOC128945956 | protein_coding | olfactory receptor 14J1-like |
| NW_026535846.1 | 3275001 | 3284802 | LOC128945595 | lncRNA | uncharacterized LOC128945595 |
| NW_026535846.1 | 3300001 | 3326670 | LOC128945590 | protein_coding | protein fantom-like |
| NW_026535846.1 | 3325001 | 3326670 | LOC128945590 | protein_coding | protein fantom-like |
| NW_026535846.1 | 3284814 | 3325000 | LOC128945590 | protein_coding | protein fantom-like |
| NW_026535846.1 | 3368027 | 3372136 | LOC128945593 | protein_coding | 40S ribosomal protein S4 |
| NW_026535846.1 | **3375886** | **3391745** | **HDAC8** | **protein_coding** | **histone deacetylase 8** |
| NW_026535846.1 | **3393654** | **3400000** | **PHKA1** | **protein_coding** | **phosphorylase kinase regulatory subunit alpha 1** |
| NW_026535846.1 | **3393654** | **3413178** | **PHKA1** | **protein_coding** | **phosphorylase kinase regulatory subunit alpha 1** |
| NW_026535846.1 | **3400001** | **3413178** | **PHKA1** | **protein_coding** | **phosphorylase kinase regulatory subunit alpha 1** |
| NW_026535846.1 | **3418094** | **3419667** | **BMP15** | **protein_coding** | **bone morphotic protein 15** |
| NW_026535846.1 | **3423272** | **3425000** | **SHROOM4** | **protein_coding** | **shroom family member 4** |
| NW_026535846.1 | **3423272** | **3434448** | **SHROOM4** | **protein_coding** | **shroom family member 4** |
| NW_026535846.1 | **3438642** | **3450000** | **DGKK** | **protein_coding** | **diacylglycerol kinase kappa** |
| NW_026535846.1 | 3441207 | 3441303 | LOC128946012 | snoRNA | small nucleolar RNA SNORA17 |
| NW_026535863.1 | 83069 | 97654 | LOC128946758 | protein_coding | copine-5-like |
| NW_026535863.1 | **98944** | **100000** | **PPIL1** | **protein_coding** | **peptidylprolyl isomerase like 1** |
| NW_026535863.1 | **98944** | **101788** | **PPIL1** | **protein_coding** | **peptidylprolyl isomerase like 1** |
| NW_026535863.1 | **102140** | **105129** | **PSRC1** | **protein_coding** | **proline and serine rich coiled-coil 1** |
| NW_026535863.1 | **105228** | **125000** | **CELSR2** | **protein_coding** | **cadherin EGF LAG seven-pass G-type receptor 2** |
| NW_026535863.1 | **125001** | **136271** | **CELSR2** | **protein_coding** | **cadherin EGF LAG seven-pass G-type receptor 2** |
| NW_026535863.1 | **137625** | **145042** | **SARS1** | **protein_coding** | **seryl-tRNA synthetase 1** |
| NW_026535863.1 | **145772** | **158523** | **ELAPOR1** | **protein_coding** | **endosome-lysosome associated apoptosis and autophagy regulator 1** |
| NW_026535863.1 | 160773 | 161072 | LOC128946855 | ncRNA | small Cajal body-specific RNA 2 |
| NW_026535863.1 | **161596** | **163397** | **TMEM167B** | **protein_coding** | **transmembrane protein 167B** |
| NW_026535863.1 | **168758** | **175000** | **INAVA** | **protein_coding** | **innate immunity activator** |
| NW_026535863.1 | **2415484** | **2425000** | **PNPLA1** | **protein_coding** | **patatin like phospholipase domain containing 1** |
| NW_026535873.1 | 1121324 | 1125000 | LOC128947317 | protein_coding | olfactory receptor 14J1-like |
| NW_026535873.1 | 1482392 | 1500000 | LOC128947315 | protein_coding | uncharacterized LOC128947315 |
| NW_026535873.1 | 1498215 | 1500000 | LOC128947313 | protein_coding | toll-like receptor 13 |
| NW_026535896.1 | 6567738 | 6568670 | LOC128948302 | protein_coding | olfactory receptor 14J1-like |
| NW_026535896.1 | 6599871 | 6600000 | LOC128948304 | protein_coding | olfactory receptor 14J1-like |
| NW_026535957.1 | 895744 | 900000 | LOC128949032 | protein_coding | protein ecdysoneless homolog |
| NW_026535957.1 | 2122736 | 2123668 | LOC128949029 | lncRNA | uncharacterized LOC128949029 |
| NW_026535993.1 | 975001 | 1003783 | LOC128949387 | protein_coding | olfactory receptor 14J1-like |
| NW_026535993.1 | 1013375 | 1014319 | LOC128949438 | protein_coding | olfactory receptor 6M1-like |
| NW_026535993.1 | 1015374 | 1019792 | LOC128949388 | pseudo | olfactory receptor 14J1-like |
| NW_026535993.1 | 2011284 | 2012222 | LOC128949451 | protein_coding | olfactory receptor 14J1-like |
| NW_026535993.1 | 2045295 | 2049877 | LOC128949408 | protein_coding | olfactory receptor 14J1-like |
| NW_026535993.1 | 2059483 | 2060427 | LOC128949431 | protein_coding | olfactory receptor 49-like |
| NW_026535993.1 | 2853617 | 2858664 | LOC128949413 | protein_coding | serine/threonine-protein kinase pim-1-like |
| NW_026535993.1 | 2862391 | 2895705 | LOC128949415 | pseudo | cell division cycle protein 20 homolog |
| NW_026536003.1 | 575001 | 587411 | LOC128949488 | pseudo | zinc finger protein 883-like |
